# Design of New Daunorubicin Derivatives with High Cytotoxic Potential

**DOI:** 10.3390/ijms26031270

**Published:** 2025-01-31

**Authors:** Aleksandra A. Kalashnikova, Altynkul B. Toibazarova, Oleg I. Artyushin, Lada V. Anikina, Anastasiya A. Globa, Zinaida S. Klemenkova, Maxim V. Andreev, Eugene V. Radchenko, Vladimir A. Palyulin, Yulia R. Aleksandrova, Marat I. Syzdykbayev, Nurbol O. Appazov, Vladimir N. Chubarev, Margarita E. Neganova, Valery K. Brel

**Affiliations:** 1 Nesmeyanov Institute of Organoelement Compounds, Russian Academy of Sciences, Vavilova St. 28, Bld. 1, Moscow 119991, Russia; ma@ineos.ac.ru (A.A.K.); oleg.artuyshin@gmail.com (O.I.A.); zklem@ineos.ac.ru (Z.S.K.); hcc.andreev@gmail.com (M.V.A.); yulia.aleks.97@mail.ru (Y.R.A.); 2 Laboratory of Engineering Profile, Korkyt Ata Kyzylorda University, Ayteke bi Str., 29A, Kyzylorda 120014, Kazakhstan; toibazarovaaltynkul@gmail.com; 3 Institute of Physiologically Active Compounds of the FSBIS of the Federal Research Center for Problems of Chemical Physics and Medicinal Chemistry of the RAS, 1 Severnyi Proezd, Chernogolovka 142432, Russia; anikina1970@gmail.com (L.V.A.); globa271194@mail.ru (A.A.G.); 4 Department of Chemistry, Lomonosov Moscow State University, Leninskie Gory, Moscow 119991, Russia; genie@qsar.chem.msu.ru (E.V.R.); vap@qsar.chem.msu.ru (V.A.P.); 5 Department of Biology, Geography and Chemistry, Laboratory of Engineering Profile, Korkyt Ata Kyzylorda University, Ayteke bi Str., 29A, Kyzylorda 120014, Kazakhstan; syzdykbayev_m@korkyt.kz; 6 Laboratory of Engineering Profile, Department of Engineering Technology, Korkyt Ata Kyzylorda University, Ayteke bi Str., 29A, Kyzylorda 120014, Kazakhstan; nurasar.82@korkyt.kz; 7 “CNEC” LLP, Dariger Ali Lane, 2, Kyzylorda 120001, Kazakhstan; 8 Department of Pharmacology, The Institute of Pharmacy Named after A.P. Nelyubin, Sechenov University, Trubetskaya St., 8-2, Moscow 119991, Russia; chubarev_v_n@staff.sechenov.ru

**Keywords:** anthracycline antibiotics, chemotherapy, daunorubicin, cytotoxicity, cell cycle, glycolysis

## Abstract

Chemotherapy with anthracycline antibiotics is a common method of treating tumors of various etiologies. To create more highly effective cytostatics based on daunorubicin, we used the method of reductive amination using polyalkoxybenzaldehydes. The obtained derivatives of the anthracycline structure have much greater cytotoxicity compared to daunorubicin due to increased affinity for DNA, the ability to disrupt the cell cycle, and their inhibition of the glycolysis process, which is confirmed by data from extensive biological studies and the results of molecular modeling.

## 1. Introduction

**Figure 1:**
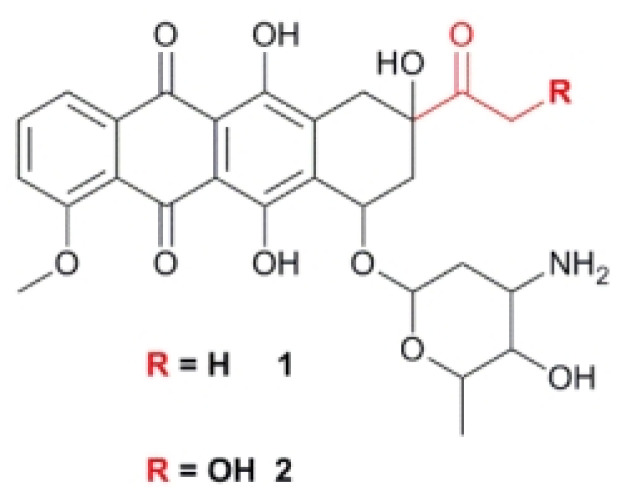
Daunorubicin **1**; doxorubicin **2**.

In the treatment of cancer patients, chemotherapy with cytostatics of natural origin [1], in particular anthracycline antibiotics (e.g., doxorubicin, daunorubicin, epirubicin, and valrubicin), is widely used in clinical practice. Daunorubicin **1** (Figure 1) is effective against both acute and chronic leukemias, as well as solid tumors [2,3]. Doxorubicin **2** is routinely used for the therapy of various cancers, such as those located in the stomach, lungs, ovaries, breast, etc. However, despite the high efficiency of anthracyclines, their use is limited by high toxicity levels [4] and extremely negative impacts on the cardiovascular and reproductive systems of patients, as well as on liver cells, the lungs, and bone marrow [5]. To reduce the extensive range of anthracycline antibiotics’ side effects on patients, various techniques are considered, such as a multimodal therapy or their use in combination with cardioprotective drugs [6,7,8,9]. The most effective approach is the structural modification of chemotherapeutic drugs, which leads to a significant reduction in the overall toxicity of cytostatics. [10]. A literature review from 2000 onwards has revealed over 3500 scientific articles about daunorubicin and doxorubicin functionalization [11] to reduce their side effects.

**Figure 2:**
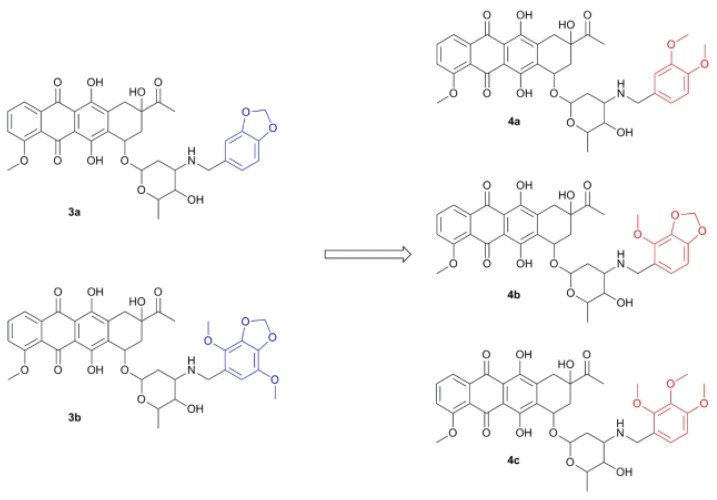
Extended daunorubicin derivatives.

Methods of modifying the amino sugar moiety, namely N- and O-functionalization of amine and hydroxyl groups, have been described in sufficient detail. Reactions involving the hydroxyl group yield tetrahydropyranyl derivatives of anthracyclines [12], some disaccharide analogues [13,14], and cyclophosphamide compounds [15]. The transformation of the anthracycline amino group plays a crucial role in altering cytotoxic activity. Entire libraries of cytostatics can easily be prepared by N-functionalization of daunorubicin or doxorubicin. Among the anthracycline derivatives known in the literature are diamine, triamine [16,17], amide [18,19], and carbamate [20] compounds, as well as molecules with various functional groups containing small [21] and medium [22] cycles, iodine and fluorine [23], selenium and sulfur [24], and phosphorus [15,25]. Mono- and oligosaccharides [26], peptides [27], hybrid molecules [28], and even bis-anthracyclines [29] are also of interest as potential dual-action drugs. Our previous works have shown that in terms of biological response, it is advisable to obtain secondary and tertiary amines with an anthracycline structure [30,31]. Compounds **3a**,**b**, which we obtained by the reductive amination method, turned out to be extremely active cytostatics against four tumor cell lines with a lower (compared to daunorubicin) acute toxicity rate [32]. Therefore, in this work, we decided to prepare different polymethoxy anthracycline derivatives **4a**–**c**, etc., which are structurally similar to piperonal-substituted anthracyclines, which we previously obtained [30,32] (Figure 2).

**Figure 3:**
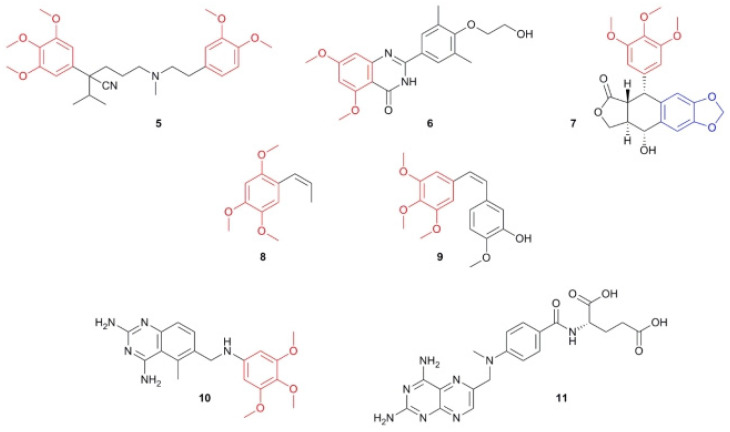
Biologically active molecules with polymethoxybenzyl pharmacophore groups: gallopamil **5** [33], apabetalone **6** [34], deoxypodophyllotoxin **7** [35], β-asarone **8** [36], combretastatin A-4 **9** [37], trimetrexate **10**, and methotrexate **11** [38].

For this purpose, we developed a method for the functionalization of daunorubicin by introducing a polymethoxybenzyl fragment, which is found in various natural compounds (Figure 3) and is also widely used as an effective pharmacophore in drugs’ structure [33,34,35,36], including those with cytotoxic activity [37,38], in the amino group of the carbohydrate part.

When studying the biological activity of new daunorubicin derivatives, we focused not only on the analysis of the cytotoxic profile but also on determining the mechanisms of cytotoxicity. For this purpose, we studied the effect of the compounds on the cell cycle and their ability to modulate the main metabolic pathway of tumor cell glycolysis. Moreover, using molecular modeling, we predicted the ability of substances to interact with DNA. In addition, we calculated ADME parameters and determined the level of acute toxicity of the leaders in in vivo experiments.

## 2. Results and Discussion

### 2.1. Chemistry

A reliable method of reductive amination allows the target daunorubicin derivatives to be obtained from a series of aromatic aldehydes [39]. We slightly improved the procedure for these purposes and used additionally purified [40] sodium cyanoborohydride (at least 98% based on the main substance). Substituted aromatic aldehydes, which appear to be the most reactive [30,32] compared to aliphatic ones [16], were used as starting materials in this transformation.

**Scheme 1:**
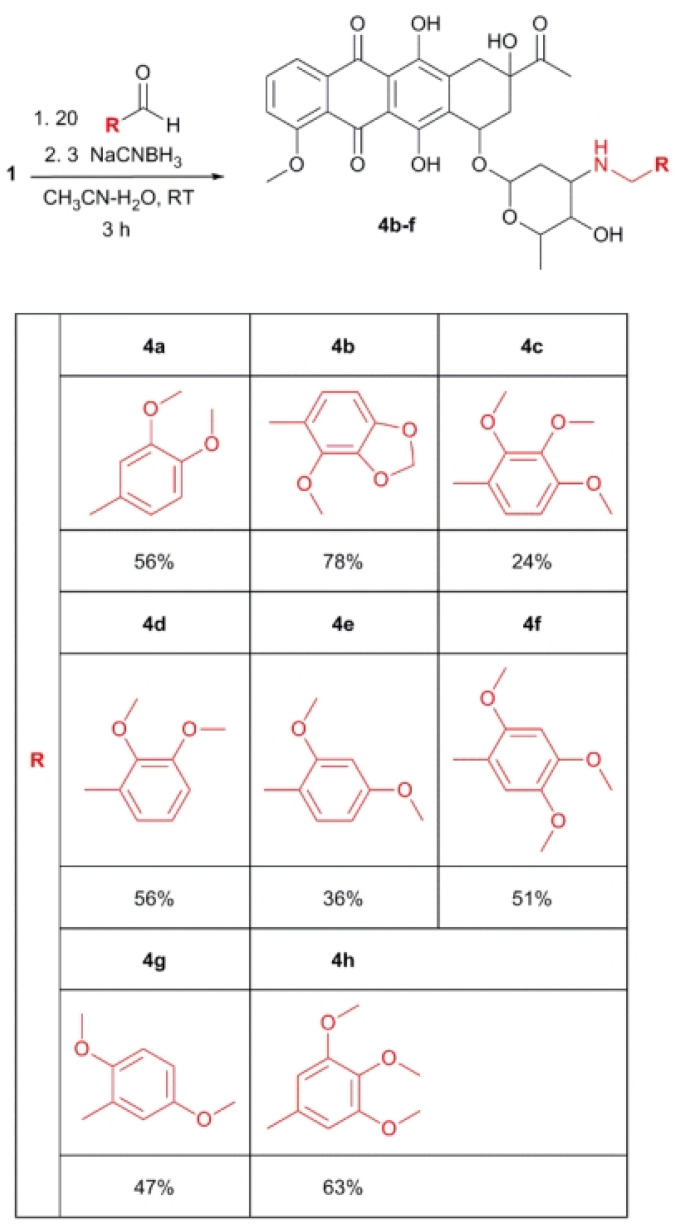
Reductive amination using aromatic aldehydes.

The reaction was carried out under standard conditions (Scheme 1). The use of a 20-fold excess of aldehyde instead of, for example, a 4-fold excess, leads to a significant increase in the yield of the product and facilitation of subsequent chromatographic purification. The composition of the N-alkylated daunorubicin derivatives **4a**–**h**, which are crystalline substances of different shades of red, was elucidated by mass spectrometry and elemental analysis, and the structure was confirmed by NMR and IR spectroscopy. The spectral data of the products were very similar to those of the daunorubicin, except for the additional signals in the ranges of 6.0–7.5 and 3.5–4.0 ppm, which indicated the presence of aromatic fragments and methoxy groups. Yields varied from 24 to 78%.

**Figure 4:**
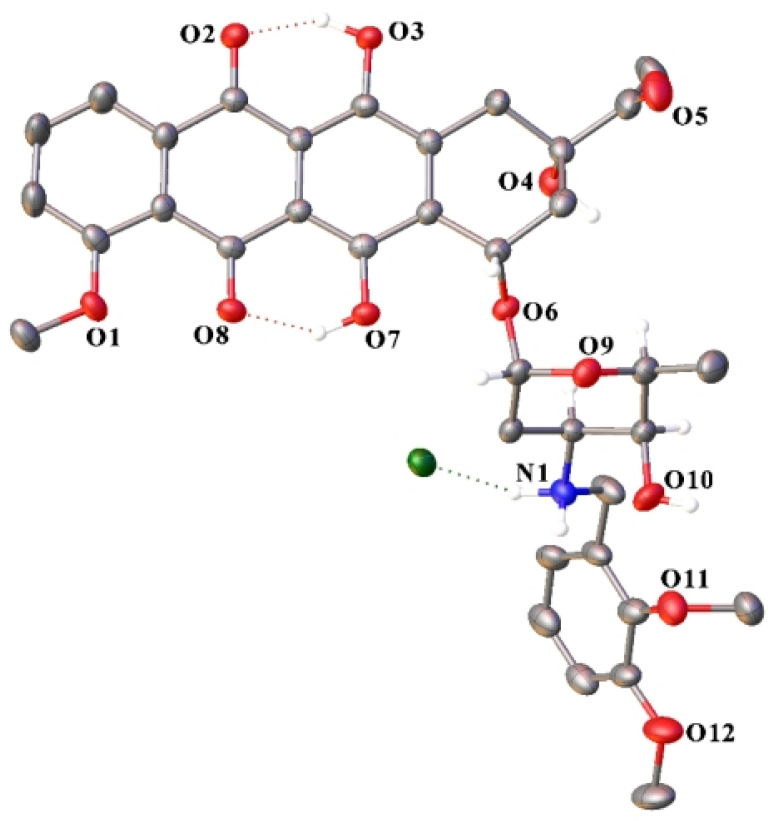
Crystal structure of compound **4d**, showing the atomic numbering of selected heteroatoms. Hydrogen atoms with the exception of those at heteroatoms and at chiral centers, solvent molecules, and second symmetry-independent species were omitted for clarity. Non-hydrogen atoms are shown as thermal ellipsoids (p = 50%).

The structure of compound **4d** was confirmed by X-ray diffraction data (Figure 4).

The compound **4d** crystallizes in the chiral orthorhombic P212121 space group with two protonated cations, two chloride anions, three disordered and partially occupied solvent molecules of acetone, and two partially occupied water molecules in the asymmetric unit. The cations consist of an aglycone, containing three approximately planar fused rings (the root mean square deviations of the six rings in the asymmetric unit are between 0.004 and 0.015 Å), an attached sugar moiety in a chair conformation, and an aromatic functional group with two methoxy substituents in ortho- and para-positions, respectively. Two aglycone fragments and one aromatic functional group form a stack-like association due to the parallel-displaced stacking interactions between two aglycone fragments with inter-centroid and shift distances of 3.599(2) Å and 1.164(6) Å, respectively, and an angle of 1.86(13)°, and the stacking interaction between aglycone and phenyl group fragments with inter-centroid and shift distances of 3.569(6) and 1.314(8) Å, respectively, and an angle of 15.5(3)°. These stacks form zigzag-like three-dimensional framework stabilized by the intermolecular interactions between chloride anions and cations (O…Cl 3.100(3)–3.115(3) Å, OHCl 148.59(18)–156.24(17)° and N…Cl 3.037(4)–3.149(4) Å, NHCl 132.8(2)–165.4(2)°), and intermolecular O…O and N…O hydrogen bonds between cations and solvent molecules (O…O 2.687(8)–2.823(4) Å, OHO 152.7(5)–159.4(2)° and N…O 2.761(5)–2.885(8) Å, NHO 163.6(2)–174.5(3)°).

### 2.2. Biological Research

#### 2.2.1. Cytotoxic Profile

A number of the compounds obtained showed activity in the in vitro MTT assay. The results of cytotoxicity against the human tumor cell line are summarized in Table 1. Of the eight compounds, four showed cytotoxicity equivalent to that of daunorubicin (**4a**, **4b**, **4g**, **4h**), and two showed cytotoxicity greater than daunorubicin (**4e** and **4f**).

We found that the pseudo-normal human embryonic kidney (HEK293) cells were the least sensitive to daunorubicin and its derivatives. However, the calculated selectivity indices of most of the newly synthesized molecules exceeded those for the original anthracycline (in some cases by more than 50 units, for example, for **4a** and **4h** in relation to the HCT116 cell line and **4e** and **4f** in relation to the A549 cell line), which indicates the promise of the chemical modification of daunorubicin in obtaining molecules with a selective effect specifically on cells of tumor origin.

The HEK293 cell line was the least sensitive to the synthesized daunorubicin-based compounds; differences were also observed with respect to other tumor cell lines. Firstly, MCF7 cells were more sensitive to the tested substances than to daunorubicin itself. Secondly, six derivatives were more cytotoxic than daunorubicin with respect to A549 cells.

It was shown that changes in the mutual arrangement of methoxy groups in the aryl radical strongly affect the survival of tumor cells. Cytotoxicity practically disappears in the case of compound **4c** and is 400 times higher than that of daunorubicin in the case of compound **4e**, although these compounds differ only in the presence or absence of a methoxy group in the meta position of the substituent. Highly active and less active compounds were found among both di- and trimethoxy derivatives. The combination of ortho- and para-positions of methoxy substituents had a positive effect on the development of cytotoxicity; this is proven by the data obtained for the two most active compounds **4e** and **4f**, which have such a combination of substituents. Thus, the cytotoxicity exhibited by the substances does not depend on the number of methoxy groups, but rather on where and how they are located in the polyalkoxybenzene substituent.

The cytotoxicity of compounds **4e** и **4f** exceeded that of daunorubicin in all the cultures studied, and this difference was 10–20 times for the MCF7 culture and 460 times for the A549 culture. This phenomenon was of particular interest to us, and we attempted to clarify its nature and determine the possible mechanisms of the cytotoxic action of the synthesized derivatives. To do this, we analyzed the effect of the substances on the cell cycle and glycolysis and also performed molecular modeling to determine the ability of the compounds to bind to DNA.

#### 2.2.2. Cell Cycle Analysis

Disruptions in the cell cycle control system are one of the main causes of malignant tumor formation. Mutations in genes encoding the regulatory proteins of the cell cycle lead to uncontrolled cell division, further accumulation of mutations in cells, and the appearance and spread of aneuploid cells with abnormal chromosomal content, etc. In turn, the action of many anticancer drugs is accompanied by a slowdown or blockade of the cell cycle [41]. By measuring DNA content, it is possible to study populations of cells at different stages of the cell cycle and analyze DNA ploidy. In this population, cells are distributed across three main phases of the cell cycle: the G0/G1 phase (one set of paired chromosomes per cell), the S phase (variable DNA synthesis), and the G2/M phase (two sets of paired chromosomes per cell before cell division).

**Figure 5:**
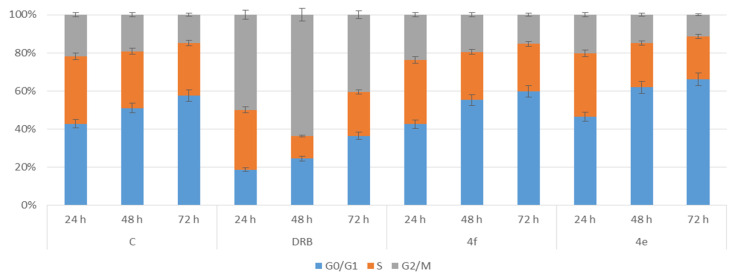
Histograms of the Jurkat cell line showing the distribution of DNA content when exposed to compounds **4e** and **4f** in comparison with daunorubicin (DRB) after 24, 48 and 72 h.

In an experiment, the effect of the studied compounds on the cell cycle of the Jurkat line was evaluated. Histograms of Jurkat cells stained with FxCycle™ showing DNA content distribution are presented in Figure 5.

Anthracyclines cause DNA damage, including single-strand and double-strand breaks, which occur as a result of interaction with reactive oxygen species formed during the redox cycle, as well as inhibition of topoisomerase II [42]. DNA damage leads to activation of cell cycle checkpoint signaling pathways, causing cells to arrest in the G1 or G2/M phases for DNA repair [43]. In previous studies, Czyż et al. showed that K562 cells treated with anthracyclines arrested in the G2/M phase [44]. In another experiment, Denel-Bobrowska et al. found that the greatest shift in the cell cycle towards the G2/M phase was observed in cells treated with doxorubicin and daunorubicin after 48 h [45].

During our experiment, it was found that daunorubicin induced cell cycle arrest in the G2/M phase throughout the entire study period, with a peak at 48 h after exposure; 63% of cells were in this phase compared to 19% in the control group of intact cells. The G2 checkpoint prevents cells from entering mitosis when DNA damage is present, thus allowing for repair and preventing proliferation of damaged cells [46]. Prolonged delay of mitosis in the G2/M phase leads to apoptosis, thereby realizing the antiproliferative effect of daunorubicin.

Daunorubicin derivatives have a different effect on the cell cycle than daunorubicin itself. They contribute to an increase in the proportion of cells in the G0/G1 phase and a decrease in the number of cells in the synthetic and premitotic phases. In the case of the more cytotoxic compound **4e**, this effect was stronger; under the influence of compound **4e**, 61% of the cells were in the G0/G1 phase, while under the influence of compound **4f**, this figure was 55%, compared to 50% intact cells after 48 h of incubation (see Supplementary Materials).

#### 2.2.3. Glycolysis

For more than two decades, metabolic reprogramming has been widely recognized as a hallmark of tumor cells and a contributor to the development of malignant neoplasms [46,47,48]. It is now clear that tumor initiation and progression rely heavily on aberrant cellular metabolism to meet the high demands for ATP generation and macromolecular biosynthesis, even under normoxic conditions [48].

As shown in Table 2, when assessing the metabolic changes in A549 tumor cells under the action of compounds at a concentration of 10 μM, molecules **4a**, **4b***,* **4e**, **4f**, **4g** and **4h** as well as the original daunorubicin reliably reduced the rate of extracellular acidification of the medium by cells, thereby reducing their glycolytic activity. It should be noted that the most promising glycolysis inhibition profile was demonstrated for conjugates **4a**, **4e**, **4f** and **4g**, which exhibited the most pronounced cytotoxic effect in the series of experiments described above. Thus, these compounds reduced glucose-dependent glycolysis by more than 3 times, glycolytic capacity by 2 times and glycolytic reserve by 1.5 times, initiating a number of fatal events in tumor cells.

**Figure 6:**
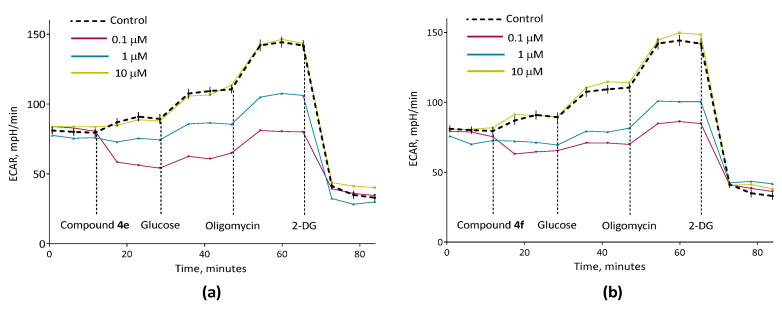
Extracellular acidification rate (ECAR) of A549 cells in a glycolysis stress test using a Seahorse XFe96 Cell Metabolism Analyzer (**a**,**b**). The results are presented as kinetic curves of changes in the rate of extracellular acidification of the cells after sequential administration of the test compounds (0.1, 1, and 10 μM), glucose (10 mM), oligomycin (1 μM), and 2-deoxyglucose (2-DG) (50 μM). Data are presented as mean ± SEM, n = 3.

It is interesting that during subsequent assessment of cell viability after ~80 min of exposure of cells to the studied substances, it was found that the synthesized compounds did not lead to cell death, while in the samples treated with daunorubicin, cell viability was reduced by 32%. This may indicate that the decrease in the rate of extracellular acidification of the medium in the samples with daunorubicin is most likely due to their acute cytotoxicity, rather than glycolysis-inhibiting action. In addition, in order to identify the concentration-dependent effect of the most effective compounds **4a, 4e, 4f** and **4g**, we studied their effect on the glycolysis process in a concentration range from 0.1 to 10 μM. Thus, Figure 6 shows representative images of kinetic curves showing the change in the rate of acidification of the external cell medium by A549 cells. The graphs clearly show a clear dependence of the inhibitory effect of the molecules on the concentration.

Thus, based on the noted features, the obtained results allow us to assume the contribution of polyalkoxybenzaldehydes to the emergence of glycolysis-inhibiting properties.

To clarify the possible mechanism of glycolysis-inhibiting action of the most active molecules **4e** and **4f**, we assessed the effect of the compounds on the activity of recombinant enzymes that catalyze irreversible reactions in the process of glycolysis–hexokinase, phosphofructokinase and pyruvate kinase.

**Figure 7:**
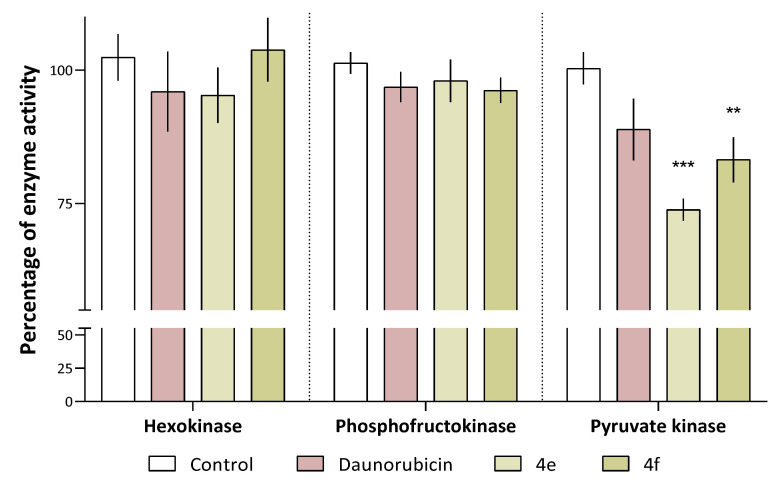
Effect of daunorubicin derivatives on the activity of rate-limiting enzymes of glycolysis–hexokinase, phosphofructokinase, and pyruvate kinase and the concentration of the studied compounds (1 μM). Data are presented as mean ± SEM, n = 3. ** and ***—*p* < 0.01 and *p* < 0.001, respectively, compared with the control (by one-way ANOVA with Dunnett’s test).

In accordance with the results presented in Figure 7, we demonstrated the pronounced ability of **4e** and **4f** to significantly inhibit the activity of pyruvate kinase, which catalyzes the final reaction of converting phosphoenolpyruvate into pyruvate. Thus, the activity of this enzyme was reduced by up to 36% under the influence of **4e**.

In this case, the fact that the targeting of pyruvate kinase by molecules can be due to the modification of the original daunorubicin molecule by polyalkoxybenzaldehydes is also confirmed, since for daunorubicin itself, although the ability to reduce enzyme activity was revealed, it was not reliable.

#### 2.2.4. Acute Toxicity In Vivo

Acute toxicity values were also determined for the most active compounds **4e** and **4f**. The LD_50_ of compound **4f** was 59.0 mg/kg; therefore, according to the classification of K.K. Sidorov [49], which is used for parenterally administered agents, this substance belongs to the class of moderately toxic substances (class 3; 41–85 mg/kg). Compound **4e** had an LD_50_ of 112.0 mg/kg, so according to the same classification, this compound can be categorized as minimally toxic (98–127 mg/kg). At the same time, the LD_50_ of daunorubicin when administered intraperitoneally to mice was 1.8 mg/kg [50], which makes it a highly toxic substance (class 2) [49].

### 2.3. Molecular Modeling

To determine the probable mechanism of action of modified daunorubicin analogues, the potential interactions of the compounds with DNA duplex were analyzed using molecular docking and molecular dynamics modeling.

An analysis of the literature reveals that the basic binding features are conserved in all the studied DNA complexes of daunorubicin, doxorubicin, and their modified analogs; the anthracycline system intercalates between the CG–GC base pairs, while the sugar residue and the groups linked to it (if any) are bound in the minor groove, preferably near the neighboring TA base pair (see, e.g., PDB: 1AL9, 288D, 2DES). Taking this into account, the self-complementary duplex (ACGTACGT)_2_ based on the structure of its bis-daunorubicin complex (PDB: 1AL9) was used in the modeling. Upon removal of the bound ligand, the unmodified daunorubicin molecule was docked into one of the symmetrical binding sites with the sugar residue oriented outwards the double helix. This allowed us to stabilize the complex structure and to avoid potential artifacts caused by the interaction between the ligands bound in the two sites. The test compounds (daunorubicin DNR, **4a**, **4e**, **4d**, **4f**, **4c**) were then automatically or (if necessary) manually docked into the second binding site in the approximately correct pose.

Afterwards, the molecular dynamics simulation of the system was performed for 200 ns (for the detailed modeling protocol, see Materials and Methods). The RMSD plots of the DNA and ligand molecules (see Supplementary Materials) as well as the visual analysis of the trajectories revealed that the stability of the system and the basic binding mode of the ligands were retained throughout the simulation. On the other hand, for some of the ligands, the orientation of the sugar residue and the polymethoxyphenyl group may have undergone significant adjustments, but for all of them, the binding mode was stabilized by 160 ns; then, only minor thermal oscillations were observed. Thus, the most frequent complex structures were identified by clustering the trajectory frames in the 160–200 ns interval.

**Figure 8:**
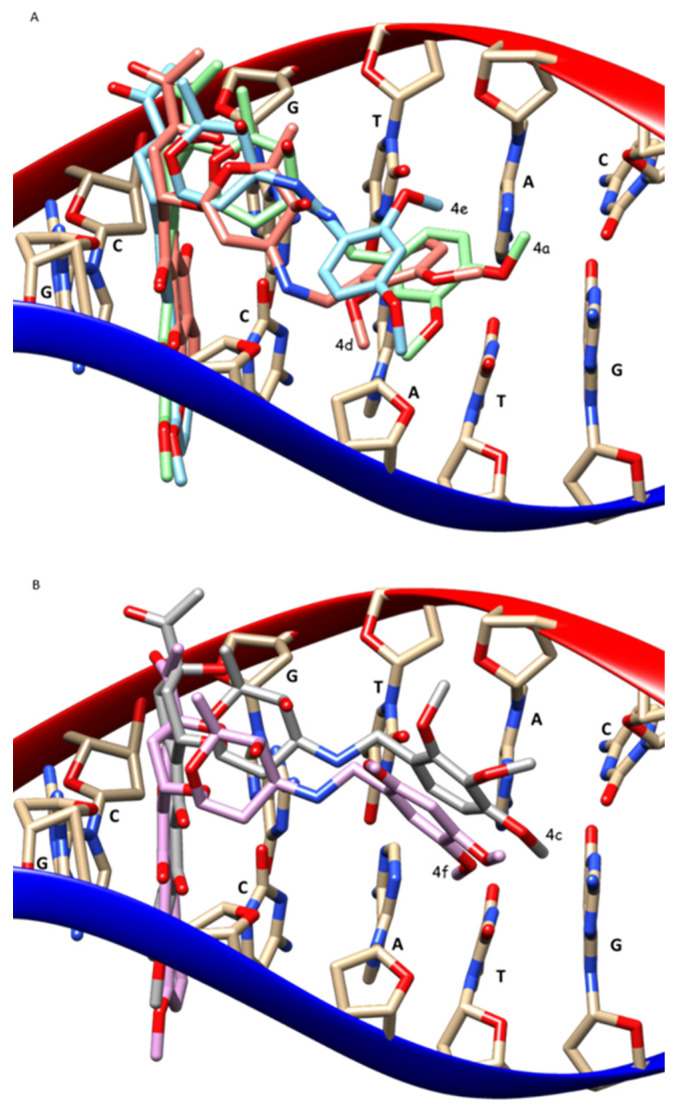
Binding modes of the compounds in the DNA duplex refined using molecular dynamics simulations. The DNA molecules are shown as beige stick models and backbone ribbons. The compounds with phenyl group parallel (**A**) or perpendicular (**B**) to the groove surface are shown as stick models: **4a** green, **4e** cyan, **4d** salmon, **4f** lavender, **4c** grey.

Interestingly, two distinct binding modes were found for these compounds, which differed in the orientation of the polymethoxyphenyl group. For the dimethoxy derivatives (**4a**, **4e**, **4d**), the phenyl group was approximately parallel to the minor groove surface (Figure 8A), while for the trimethoxy derivatives (**4f**, **4c**), it was approximately perpendicular to the surface (Figure 8B).

It can be noted that the most potent compounds (**4e** and **4f**) tend to adopt a conformation in which some of the methoxy groups are oriented towards the bases in the groove, while other methoxy groups are exposed to the solvent. One can hypothesize that such an arrangement may be advantageous for the interactions involved in the anti-topoisomerase activity of the compounds. On the other hand, in the compounds with low (**4g**) or moderate activity, all the methoxy groups are oriented towards the solvent. 

### 2.4. Drug–Likeness Analysis of Compounds

**Figure 9:**
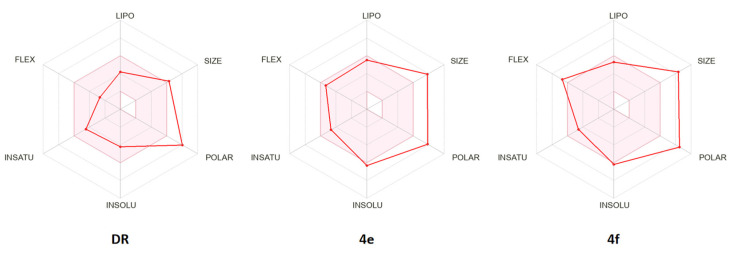
SwissADME bioavailability radar of daunorubicin, **4e** and **4f**. The pink areas represent each property (lipophilicity, molecular weight, solubility, and flexibility).

The drug-likeness of the most promising molecules **4e** and **4f** was analyzed to check whether the molecules had favorable ADME (absorption, distribution, metabolism, and excretion) properties. As evidenced by the SwissADME bioavailability radars shown in Figure 9, compound **4e** is preferred in terms of three parameters, namely lipophilicity, insaturation, and flexibility, with a slight bias towards insolubility and size and polarity. For compound **4f**, a bias was also observed in the flexibility parameter.

Although the obtained results cannot indicate the complete compliance of the synthesized molecules as “drug-likeness”, which is associated with the presence of deviations according to the Lipinski rule (Table 3), this does not leave an irreversible negative imprint on their pharmacological prospects due to the presence of similar deviations in the original daunorubicin, which has not prevented it from being included in the list of first-line chemotherapeutic drugs for many years; moreover, the drug arbidol, which does not obey the parameters of drug similarity and has poor solubility, has been successfully presented on the world pharmaceutical market and is widely used in the treatment of viral diseases.

Thus, the most pronounced profile of antitumor activity in the synthesized compounds that we discovered is of genuine interest, in particular in conducting further optimization of the lead compounds in order to obtain both more effective molecules and compounds with an improved pharmacokinetic profile.

## 3. Materials and Methods

### 3.1. Synthesis and General Procedures

Multinuclear ^1^H and ^13^C NMR spectra were recorded on Bruker Avance 400 (Bruker, MA, USA) and Inova 400 spectrometers (Bruker, MA, USA) (at 400.13 and 100.61 MHz operating frequencies, respectively), and a Bruker Avance 300 spectrometer (at a 300.13 MHz operating frequency) in CDCl_3_ solutions using residual proton signals of the deuterated solvent (^1^H, ^13^C) as internal references. ^13^C NMR spectra were recorded mostly in ^13^C{^1^H} jmod mode, as the signals of carbon atoms with even and odd numbers of protons have opposite polarity. The signals of daunorubicin derivatives were assigned in accordance with data from the literature [51].

IR spectra were obtained for sample pellets in KBr on an FT-IR spectrometer (InfraRed Bruker Tensor 37, Waltham, MA, USA) in a 400–4000 cm^−1^ range with 2 cm^−1^ resolution and 32 scans.

Liquid chromatography–mass spectrometry (LC–MS) measurements were performed on a Shimadzu LCMS-2020 (Tokyo, Japan) instrument using electrospray ionization (ESI) and a single quadrupole mass detector. Data were collected between *m/z* 50 and *m/z* 2000 in a positive ion mode. The potential difference between the spray capillary and the injection capillary was 4.5 kV. The value of scan speed was set to 5000 u/sec. The following settings were used: nebulizing gas (99.999% nitrogen) flow 1.5 L/min, drying gas (99.999% nitrogen) flow 15 L/min, interface temperature 350 °C, desolvation line temperature 250 °C, heat block temperature 400 °C, pump LC-20ADXR flow 0.4 mL/min, pump pressure 7.5 MPa. Acetonitrile, used as a mobile phase, was of HPLC grade.

HPLC-MS analysis was performed on a Shimadzu LCMS-2020 (Japan) instrument using electrospray ionization (ESI) or DUIS mode ionization (ESI and atmospheric pressure chemical ionization (APCI) together) and a single quadrupole mass detector. Data were collected between *m/z* 100 and *m/z* 1500 in a positive ion mode. The potential difference between the spray capillary and the injection capillary was 4.5 kV. The scan speed was set to 1500 u/sec. The above settings of the main blocks’ temperature and gas flow were used. The oven temperature was 40 °C. A Shim-pack GIST 3 μm C18 3 × 150 mm column was used as a stationary phase. The gradient elution parameters were changed from the 1st minute to the 9th minute (from 30 to 80 vol % of acetonitrile (99.9%) and from 70 to 20 vol % of formic acid (0.1 vol %) solution in water (Milli-Q), respectively), and the total flow rate was 0.8–1.0 mL/min.

High-resolution mass spectra (HRMS) were measured on a Bruker micrOTOF II instrument using electrospray ionization (ESI) [52]. The measurements were performed in a positive ion mode (interface capillary voltage—4500 V) or in a negative ion mode (3200 V) and with a mass range from *m*/*z* 50 to *m*/*z* 3000; external or internal calibration was performed with ESI Tuning Mix Agilent. A syringe injection was used for solutions in acetonitrile, methanol, or water (flow rate 3 μL/min). Nitrogen was applied as a dry gas; the interface temperature was set at 180 °C.

Sample preparation for all MS methods consisted of dissolving 1 mg of each compound in 1 mL of acetonitrile, from which 0.1–5 μL was injected into the system. 

X-ray diffraction data were collected at 100 K with a Bruker D8 Quest CMOS diffractometer using graphite monochromated Mo-Kα radiation (λ = 0.71073 Å, ω-scans).

Reactions were monitored by TLC on alumina TLC plates w/UV254. The chromatographic purification of the compounds was carried out on a Macherey–Nagel silica gel (MN Kieselgel 60, 70–230 mesh) using the solvent system CHCl_3_:MeOH from 100:0.1 to 100:10. Daunorubicin hydrochloride was purchased from Aldrich. Commercially available starting materials were used without further purification. All obtained daunorubicin derivatives decomposed when heated above 200 °C. All products formed strong complexes with CHCl_3_, which did not decompose even after prolonged exposure to P_2_O_5_ in an oil pump vacuum.

### 3.2. General Procedure of Synthesis of Amines ***4a**–**h***

A mixture of the corresponding carboxaldehyde (8.0 mmol) and daunorubicin hydrochloride (0.23 g, 0.4 mmol) in a CH_3_CN–H_2_O mixture (3:1, 9 mL) was stirred for 0.5 h at ambient temperature in the dark. Next, NaCNBH_3_ (0.08 g, 1.2 mmol) was added, the resultant reaction mixture was stirred for 0.5 h, 5 mL of water was added, and the mixture was extracted with CHCl_3_ (3 × 10 mL). The organic layer was washed with H_2_O (15 mL) and the aqueous layer was extracted with CHCl_3_ (15 mL). The combined organic extracts were dried with Na_2_SO_4_ and filtered, and the solvent was removed under reduced pressure (10 mm Hg). The residue was purified by column chromatography on silica gel.

The obtained (*8S*,*10S*)-8-Acetyl-6,8,11-trihydroxy-10-([(*2R*,*4S*,*5S*,*6S*)-5-hydroxy-6-methyl-4-((3,4-dimethoxybenzyl)amino)tetrahydro-*2H*-pyran-2-yl]oxy)-1-methoxy-7,8,9,10-tetrahydrotetracene-5,12-dione is detailed below (**4a**). The daunorubicin derivative **4a** was obtained previously using the same method [53]. 
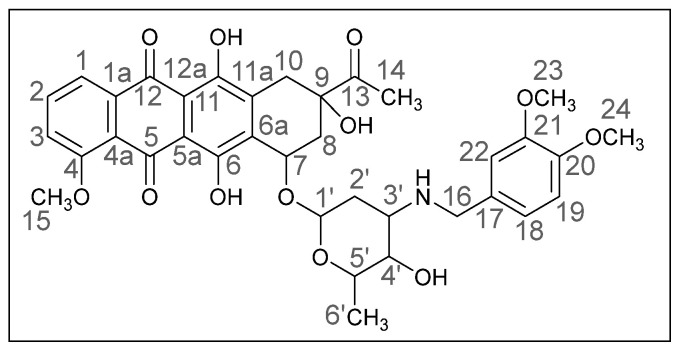


Dark red powder [3] (0.17 g, 56%); mp: (decomp.) > 200 °C; IR (KBr, cm^−1^): 3469brm (OH), 2968m, 2935m and 2837m (three CH), 2251vw, 1717m (C=O), 1617s and 1578s (both C=C), 1516s, 1446s and 1415s (both OH), 1378m, 1352m, 1286vs, 1263s, 1234s, 1209s (C–O), 1124s, 1083m, 1069m, 1032s, 988vs (C=C), 948m, 875m, 813m, 764m, 729w, 465w; ^1^H NMR (400 MHz; CDCl_3_; Me_4_Si): δ 14.02 (s, 1H, C^6^OH), 13.35 (s, 1H, C^11^OH), 8.07 (d, 1H, *^3^J_HH_* = 7.8, C^1^H), 7.82 (t, 1H, *^3^J_HH_* = 8.0, C^2^H), 7.42 (d, 1H, *^3^J_HH_* = 8.0, C^3^H), 6.81 (br. s, 3H, C^22^H, C^18^H, C^19^H), 5.55 (br. s, 1H, OH), 5.34 (br. s, 1H, C^1’^H), 4.73 (br. s, 1H, OH), 4.11 (br. s, 5H, C^15^H_3_, C^5’^H, C^7^H), 3.86 (br. s, 6H, C^23^H_3_, C^24^H_3_), 3.78 and 3.64 (d, both 1H, *^2^J_HH_* = 12.4, C^16^H_2_), 3.69 (br. s, 1H, C^4’^H), 3.27 and 3.02 (d, both 1H, *^2^J_HH_* = 19.1, C^10^H_2_), 3.02–2.98 (br. s, 1H, C^3’^H), 2.45 (s, 3H, C^14^H_3_), 2.40 and 2.13 (d, both 1H, *^2^J_HH_* = 15.2, C^8^H_2_), 1.83 (dt, 1H, *^2^J_HH_* = 13.1, *^3^J_HH_* = 4.2, C^2′^H_eq_), 1.70 (dd, 1H, *^2^J_HH_* = 13.3, *^3^J_HH_* = 4.8, C^2′^H_ax_), 1.41 (d, 3H, *^3^J_HH_* = 5.6, C^6’^H_3_) ppm; ^13^C{^1^H}NMR (100 MHz; CDCl_3_; Me_4_Si): δ 211.67 (C^13^), 186.81, 186.43 (C^5^, C^12^), 160.85 (C^4^), 156.27, 155.67 (C^6^, C^11^), 148.84, 148.06 (C^20^, C^21^), 135.57 (C^2^), 135.29 (C^1a^), 134.26, 134.12 (C^6a^, C^11a^), 132.02 (C^17^), 120.66 (C^4a^), 120.62, 120.06 (C^1^, C^3^), 118.24 (C^18^), 111.19, 111.13 (C^5a^, C^12a^), 111.04, 110.94 (C^19^, C^22^), 100.83 (C^1’^), 76.70 (C^9^), 69.72 (C^7^), 66.72, 66.47 (C^4’^, C^5’^), 56.51 (C^15^), 55.74, 55.66 (C^23^, C^24^), 52.39 (C^3’^), 50.14 (C^16^), 34.73, 33.15, 30.17 (C^8^, C^10^, C^2’^), 24.65 (C^14^), 16.97 (C^6’^) ppm; HPLC-MS (DUIS) *m/z* 678.50 ([M + H]^+^, 94%), 1355.80 ([2M + H]^+^, 7%); Retention time = 4.98.

(*8S*,*10S*)-8-Acetyl-6,8,11-trihydroxy-10-([(*2R*,*4S*,*5S*,*6S*)-5-hydroxy-6-methyl-4-(((4-methoxybenzo[*d*][1,3]dioxol-5-yl)methyl)amino)tetrahydro-*2H*-pyran-2-yl]oxy)-1-methoxy-7,8,9,10-tetrahydrotetracene-5,12-dione (**4b**).
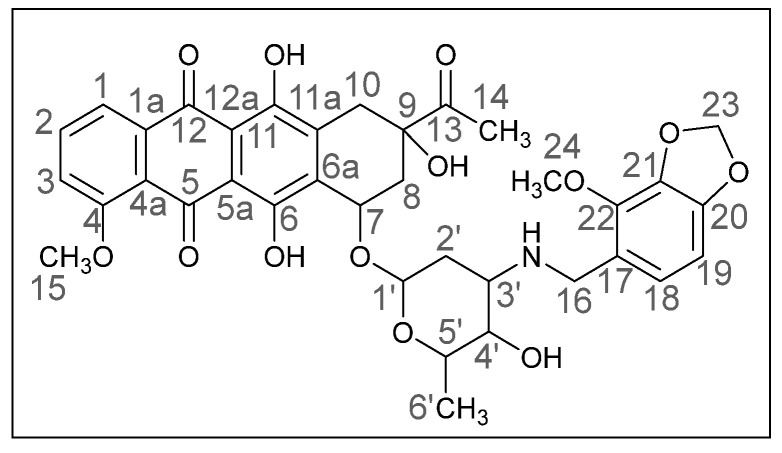


Dark red powder (0.19 g, 78%); mp: (decomp.) > 200 °C; Found: C, 56.28; H, 5.17; N, 2.21. Calc. for C_36_H_37_NO_13_·0.8CHCl_3_: C, 56.15; H, 4.84; N, 1.78%; IR (KBr, cm^−1^): 3476brm (OH), 2972shm, 2935m and 2845shw (three CH), 1716s (C=O), 1618s and 1579s (both C=C), 1469s, 1446s and 1414s (both OH), 1379m, 1352m, 1286vs, 1260s, 1232s and 1210s (both C–O), 1122s, 1068shm and 1036vs, 984s (C=C), 875w, 793m, 764w, 730w, 465vw; ^1^H NMR (400 MHz; CDCl_3_; Me_4_Si): δ 13.96 (br. s, 1H, C^6^OH), 13.21 (br. s, 1H, C^11^OH), 8.05 (d, 1H, *^3^J_HH_* = 7.3, C^1^H), 7.81 (t, 1H, *^3^J_HH_* = 8.0, C^2^H), 7.42 (d, 1H, *^3^J_HH_* = 8.3, C^3^H), 6.66 (d, 1H, *^3^J_HH_* = 8.0, C^19^H), 6.44 (d, 1H, *^3^J_HH_* = 7.8, C^18^H), 5.90 (s, 2H, C^23^H_2_), 5.54 (br. s, 1H, OH), 5.32 (br. s, 1H, C^1′^H), 4.73 (br. s, 1H, OH), 4.11 (br. s, 4H, C^35^H_3_, C^5′^H), 4.00 (s, 3H, C^24^H_3_), 3.92 (br. s, 1H, C^7^H), 3.75–3.72 (m, 2H, C^4′^H, C^16^H_eq_), 3.61 (d, 1H, *^2^J_HH_* = 11.9, C^16^H_ax_), 3.25 and 2.99 (d, both 1H, *^2^J_HH_* = 19.9, C^10^H_2_), 2.98–2.95 (m, 1H, C^3′^H), 2.46 (s, 3H, C^14^H_3_), 2.40 (d, 1H, *^2^J_HH_* = 15.7, C^8^H_eq_), 2.14–2.10 (m, 1H, C^8^H_ax_), 1.83 (dt, 1H, *^2^J_HH_* = 13.0, *^3^J_HH_* = 4.0, C^2′^H_ex_), 1.64 (dd, 1H, *^2^J_HH_* = 13.0, *^3^J_HH_* = 4.8, C^2′^H_ax_), 1.42 (d, 3H, *^3^J_HH_* = 6.5, C^6′^H_3_) ppm; ^13^C{^1^H}NMR (100 MHz; CDCl_3_; Me_4_Si): δ 211.76 (C^13^), 186.39, 186.04 (C^5^, C^12^), 160.64 (C^4^), 156.09, 155.37 (C^6^, C^11^), 148.66, 141.46 (C^20^, C^21^), 135.73 (C^22^), 135.42 (C^2^), 135.98 (C^1a^), 134.09, 134.01 (C^6a^, C^11a^), 123.87 (C^17^), 122.44 (C^18^), 120.33 (C^4a^), 119.43, 118.13 (C^1^, C^3^), 110.90, 110.75 (C^5a^, C^12a^), 101.92 (C^19^), 100.83 (C^1′^), 100.62 (C^23^), 76.58 (C^9^), 69.62, 66.31, 66.17 (C^7^, C^4′^, C^5′^), 59.29 (C^24^), 56.37 (C^15^), 51.31 (C^3′^), 44.98 (C^16^), 34.62, 32.89, 29.99 (C^8^, C^10^, C^2′^), 24.61 (C^14^), 16.96 (C^6′^) ppm; HPLC-MS (DUIS) *m/z* 692.45 ([M + H]^+^, 55%), 858.55 (13%), 856.55 (24%); Retention time = 4.54, 5.75, 6.33; HRMS (ESI) *m/z*calc’d C_36_H_37_NO_13_ [M + H]^+^ 692.2338, found 692.2337.

(*8S*,*10S*)-8-Acetyl-6,8,11-trihydroxy-10-([(*2R*,*4S*,*5S*,*6S*)-5-hydroxy-6-methyl-4-(((2,3,4-trimethoxybenzyl)amino)tetrahydro-*2H*-pyran-2-yl]oxy)-1-methoxy-7,8,9,10-tetrahydrotetracene-5,12-dione (**4c**).
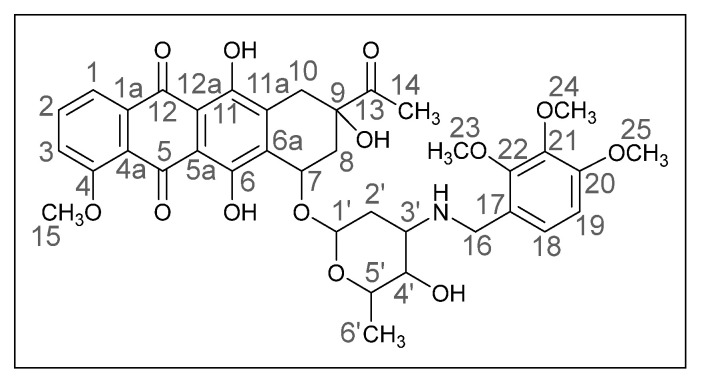


Red powder (0.06 g, 24%); mp: (decomp.) > 200 °C; Found: C, 58.24; H, 5.61; N, 1.81. Calc. for C_37_H_41_NO_13_·0.5CHCl_3_: C, 58.69; H, 5.45; N, 1.83%; IR (KBr, cm^−1^): 3489brm (OH), 2939m and 2839shw (both CH), 1751s and 1719shs (both C=O), 1618s and 1579s (both C=C), 1496s, 1470s, 1445s and 1415s (both OH), 1382m, 1352m, 1286vs, 1261s, 1233s and 1209s (both C–O), 1153w, 1122shm, 1098vs and 1069shw, 1016brs (C=C), 949w, 912w, 842shw, 792m, 761w, 698vw, 614vw; ^1^H NMR (400 MHz; CDCl_3_; Me_4_Si): δ 13.88 (br. s, 1H, C^6^OH), 13.18 (br. s, 1H, C^11^OH), 7.97 (d, 1H, *^3^J_HH_* = 7.6, C^1^H), 7.77 (t, 1H, *^3^J_HH_* = 8.2, C^2^H), 7.39 (d, 1H, *^3^J_HH_* = 8.4, C^3^H), 7.04 and 6.66 (d, both 1H, *^3^J_HH_* = 8.6, C^18^H, C^19^H), 5.41–5.38 (m, 1H, OH), 5.23 (br. s, 1H, C^1′^H), 4.69 (br. s, 1H, OH), 4.61 (br. s, 1H, C^7^H), 4.35 (dd, 1H, *^2^J_HH_* = 11.4, *^4^J_HH_* = 1.7, C^16^H_eq_), 4.15–4.11 (m, 1H, C^16^H_ax_), 4.07 (br. s, 4H, C^15^H_3_, C^5′^H), 3.95 (br. s, 1H, C^4′^H), 3.91, 3.86, 3.84 (s, all three 3H, C^23^H_3_, C^24^H_3_, C^25^H_3_), 3.14 (dd, 1H, *^2^J_HH_* = 19.0, *^3^J_HH_* = 2.1, C^10^H_eq_), 2.84 (d, 1H, *^2^J_HH_* = 18.7, C^10^H_ax_), 2.47 (d, 1H, *^2^J_HH_* = 15.2, C^8^H_eq_), 2.40 (s, 3H, C^14^H_3_), 2.37–2.35 (m, 1H, C^3′^H), 2.07 (dd, 1H, *^2^J_HH_* = 14.8, *^3^J_HH_* = 4.1, C^8^H_ax_), 1.55 (ddd, 2H, *^2^J_HH_* = 15.9, *^3^J_HH_* = 8.6, *^3^J_HH_* = 8.3, C^2′^H_2_), 1.37 (d, 3H, *^3^J_HH_* = 6.4, C^6′^H_3_) ppm; ^13^C{^1^H}NMR (100 MHz; CDCl_3_; Me_4_Si): δ 211.90 (C^13^), 186.65, 186.32 (C^5^, C^12^), 160.76 (C^4^), 156.12, 155.55 (C^6^, C^11^), 153.73 (C^20^), 151.85 (C^22^), 141.68 (C^21^), 135.56 (C^2^), 135.17 (C^1a^), 134.45, 133.60 (C^6a^, C^11a^), 124.80 (C^18^), 120.76 (C^17^), 120.53 (C^4a^), 119.56, 118.25 (C^1^, C^3^), 111.10, 110.97 (C^5a^, C^12a^), 107.22 (C^19^), 98.70 (C^1′^), 76.59 (C^9^), 73.07, 68.95, 64.42 (C^7^, C^4′^, C^5′^), 61.03, 60.61, 56.45, 55.81 (C^15^, C^23^–C^25^), 49.97 (C^3′^), 40.47 (C^16^), 35.07, 32.86, 26.17 (C^8^, C^10^, C^2′^), 24.58 (C^14^), 15.36 (C^6′^) ppm; HPLC-MS (DUIS) *m/z* 399.20 (4%), 708.45 ([M + H]^+^, 9%), 756.45 ([M + CH_3_CN]^+^, 18%); Retention time = 4.02, 7.93; HRMS (ESI) *m/z* calc’d C_37_H_41_NO_13_ [M + H]^+^ 708.2651, found 708.2664.

(*8S*,*10S*)-8-Acetyl-6,8,11-trihydroxy-10-([(*2R*,*4S*,*5S*,*6S*)-5-hydroxy-6-methyl-4-(((2,3-dimethoxybenzyl)amino)tetrahydro-*2H*-pyran-2-yl]oxy)-1-methoxy-7,8,9,10-tetrahydrotetracene-5,12-dione (**4d**).
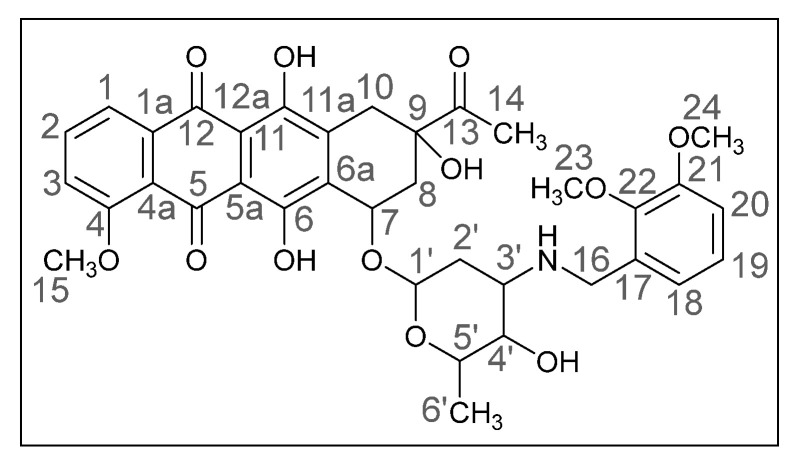


Red powder (0.06 g, 56%); mp: (decomp.) > 200 °C; Found: C, 60.18; H, 5.62; N, 2.13. Calc. for C_36_H_39_NO_12_·0.3CHCl_3_: C, 60.82; H, 5.53; N, 1.95%; IR (KBr, cm^−1^): 3476brm (OH), 2969shw, 2935m and 2838shw (three *ν*_CH_), 1717m (C=O), 1618s and 1585s (both C=C), 1474s, 1445s and 1414s (both OH), 1379m, 1352m, 1285vs, 1231s and 1209s (both C–O), 1124m, 1082m, 1034m, 1006shs and 987vs (C=C), 875m, 818m, 791m, 763m, 696vw, 467w; ^1^H NMR (400 MHz; CDCl_3_; Me_4_Si): δ 13.87 (br. s, 2H, C^6^OH, C^11^OH), 7.95 (d, 1H, *^3^J_HH_* = 7.4, C^1^H), 7.74 (t, 1H, *^3^J_HH_* = 8.1, C^2^H), 7.35 (d, 1H, *^3^J_HH_* = 8.4, C^3^H), 6.94 (t, 1H, *^3^J_HH_* = 7.9, C^19^H), 6.80–6.76 (m, 2H, C^18^H, C^20^H), 5.48 (br. s, 1H, OH), 5.22 (br. s, 1H, C^1′^H), 4.67 (br. s, 2H, OH, C^7^H), 4.05 (br. s, 4H, C^15^H_3_, C^5′^H), 3.78 and 3.77 (s, both 3H, C^23^H_3_, C^24^H_3_), 3.74–3.65 (m, 3H, C^4′^H, C^16^H_2_), 3.12 (dd, 1H, *^2^J_HH_* = 18.9, *^4^J_HH_* = 2.0, C^10^H_eq_), 2.82 (d, 1H, *^2^J_HH_* = 18.8, C^10^H_ax_), 2.94–2.90 (m, 1H, C^3′^H), 2.41 (s, 3H, C^14^H_3_), 2.34 (dd, 1H, *^2^J_HH_* = 14.8, *^3^J_HH_* = 2.6, C^8^H_eq_), 2.09–2.03 (m, 1H, C^8^H_ax_), 1.78 (dt, 1H, *^2^J_HH_* = 12.9, *^3^J_HH_* = 4.0, C^2′^H_eq_), 1.63 (dd, 1H, *^2^J_HH_* = 13.2, *^3^J_HH_* = 4.8, C^2′^H_ax_), 1.39 (d, 3H, *^3^J_HH_* = 6.5, C^6′^H_3_) ppm; ^13^C{^1^H} NMR (100 MHz; CDCl_3_; Me_4_Si): δ211.96 (C^13^), 186.77, 186.44 (C^5^, C^12^), 160.91 (C^4^), 156.36, 155.71 (C^6^, C^11^), 152.56, 147.15 (C^22^, C^21^), 135.64 (C^2^), 135.31 (C^1a^), 134.34, 134.27 (C^6a^, C^11a^), 132.98 (C^17^), 124.04 (C^18^), 121.56 (C^19^), 120.67 (C^4a^), 119.68, 118.35 (C^1^, C^3^), 111.70, 111.22, 111.07 (C^5a^, C^12a^, C^20^), 100.98 (C^1′^), 77.28 (C^9^), 69.77 (C^7^), 66.52, 66.44 (C^4′^, C^5′^), 60.82 (C^23^), 56.62, 55.68 (C^15^, C^24^), 52.07 (C^3′^), 45.02 (C^16^), 34.85, 33.19, 30.22 (C^8^, C^10^, C^2′^), 24.86 (C^14^), 17.18 (C^6′^) ppm; HPLC-MS (DUIS) *m/z* 678.45 ([M + H]^+^, 95%), 1355.85 ([2M + H]^+^, 5%); Retention time = 4.49; HRMS (ESI) *m/z* calc’d C_36_H_39_NO_12_ [M + H]^+^ 678.2545, found 678.2551.

(*8S*,*10S*)-8-Acetyl-6,8,11-trihydroxy-10-([(*2R*,*4S*,*5S*,*6S*)-5-hydroxy-6-methyl-4-(((2,4-dimethoxybenzyl)amino)tetrahydro-*2H*-pyran-2-yl]oxy)-1-methoxy-7,8,9,10-tetrahydrotetracene-5,12-dione (**4e**).
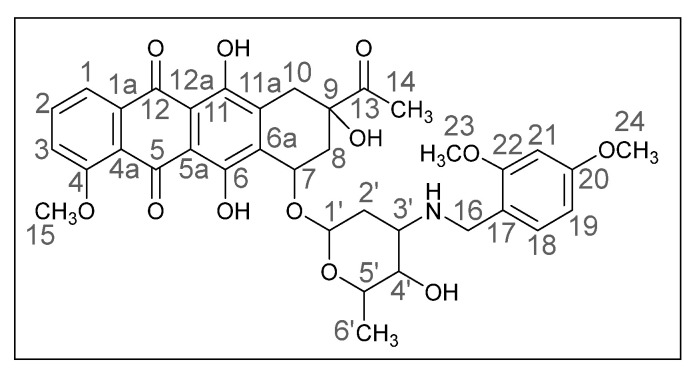


Dark red powder (0.04 g, 36%); mp: (decomp.) > 200 °C; Found: C, 60.29; H, 5.63; N, 2.14. Calc. for C_36_H_39_NO_12_·0.3CHCl_3_: C, 60.82; H, 5.53; N, 1.95%; IR (KBr, cm^−1^): 3475brm (OH), 2936m and 2837shw (both CH), 1718m (C=O), 1615m and 1587s (both C=C), 1507s, 1438shs and 1414s (both OH), 1379m, 1352m, 1288vs, 1262s, 1232s and 1209vs (both C–O), 1157m, 1127m, 1068w, 1035s and 1015shs, 988s (C=C), 830w, 793m, 764w, 464vw; ^1^H NMR (400 MHz; CDCl_3_; Me_4_Si): δ 13.88 (br. s, 1H, C^6^OH), 13.22 (br. s, 1H, C^11^OH), 7.98 (d, 1H, *^3^J_HH_* = 8.0, C^1^H), 7.75 (t, 1H, *^3^J_HH_* = 8.0, C^2^H), 7.37 (d, 1H, *^3^J_HH_* = 8.0, C^3^H), 7.01 (d, 1H, *^3^J_HH_* = 7.8, C^18^H), 6.35 (s, 1H, C^21^H), 6.32 (d, 1H, *^3^J_HH_* = 7.8, C^19^H), 5.48 (br. s, 1H, OH), 5.25 (br. s, 1H, C^1′^H), 4.80 (br. s, 1H, OH), 4.07 (br. s, 4H, C^15^H_3_, C^5′^H), 3.81–3.78 (br. s, 2H, C^7^H, C^4′^H), 3.74 (br. s, 6H, C^23^H_3_, C^24^H_3_), 3.66–3.58 (m, 2H, C^16^H_2_), 3.17 and 2.88 (d, both 1H, *^2^J_HH_* = 20.7, C^10^H_2_), 2.92 (br. s, 1H, C^3′^H), 2.43 (s, 3H, C^14^H_3_), 2.36 (d, 1H, *^2^J_HH_* = 14.0, C^8^H_eq_), 2.07 (dd, 1H, *^2^J_HH_* = 14.0, *^3^J_HH_* = 4.0, C^8^H_ax_), 1.92 (dt, 1H, *^2^J_HH_* = 13.1, *^3^J_HH_* = 3.8, C^2′^H_eq_), 1.75 (dd, 1H, *^2^J_HH_* = 12.9, *^3^J_HH_* = 4.5, C^2′^H_ax_), 1.40 (d, 3H, *^3^J_HH_* = 6.4, C^6′^H_3_) ppm; ^13^C{^1^H}NMR (100 MHz; CDCl_3_; Me_4_Si): δ 211.97 (C^13^), 186.64, 186.38 (C^5^, C^12^), 160.79 (C^4^), 159.57 (C^20^), 158.07 (C^21^), 156.30, 155.65 (C^6^, C^11^), 135.45 (C^2^), 135.26 (C^1a^), 134.35, 134.25 (C^6a^, C^11a^), 130.93 (C^18^), 120.66 (C^4a^), 120.16 (C^17^), 119.52, 118.19 (C^1^, C^3^), 111.09, 110.96 (C^5a^, C^12a^), 103.54 (C^19^), 101.36 (C^21^), 98.21 (C^1′^), 76.71 (C^9^), 69.49 (C^7^), 68.02, 66.87 (C^4′^, C^5′^), 56.59, 56.46 (C^23^, C^24^), 55.10 (C^15^), 54.99 (C^3′^), 48.10 (C^16^), 34.67, 33.15, 27.81 (C^8^, C^10^, C^2′^), 24.69 (C^14^), 17.18 (C^6′^) ppm; HPLC-MS (DUIS) *m/z*828.55 ([M + *R* + H]^+^, 42%); Retention time = 6.36; HRMS (ESI) *m/z* calc’d C_45_H_50_NO_14_ [M + *R* + H]^+^ 828.3231, found 828.3302 (see Figures S35 and S36).

(*8S*,*10S*)-8-Acetyl-6,8,11-trihydroxy-10-([(*2R*,*4S*,*5S*,*6S*)-5-hydroxy-6-methyl-4-(((2,4,5-trimethoxybenzyl)amino)tetrahydro-*2H*-pyran-2-yl]oxy)-1-methoxy-7,8,9,10-tetrahydrotetracene-5,12-dione (**4f**).
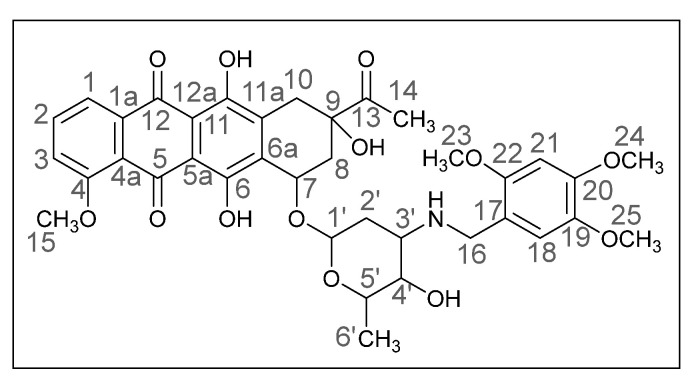


Dark red powder (0.09 g, 51%); mp: (decomp.) > 200 °C. Found: C, 58.21; H, 5.64; N, 1.84. Calc. for C_37_H_41_NO_13_·0.5CHCl_3_: C, 58.69; H, 5.45; N, 1.83%; IR (KBr, cm^−1^): 3475brm (OH), 2934m and 2839shw (both CH), 1717m (C=O), 1617m and 1578m (both C=C), 1513s, 1445s and 1413s (both OH), 1378m, 1352m, 1287vs, 1231s and 1207s (both C–O), 1124m, 1069m, 1034s, 986s (C=C), 873w, 818w, 763w, 764vw, 464w; ^1^H NMR (400 MHz; CDCl_3_; Me_4_Si): δ 13.82 (br. s, 2H, C^6^OH, C^11^OH), 7.97 (d, 1H, *^3^J_HH_* = 8.0, C^1^H), 7.75 (t, 1H, *^3^J_HH_* = 8.0, C^2^H), 7.37 (d, 1H, *^3^J_HH_* = 8.0, C^3^H), 6.71 and 6.44 (s, both 1H, C^18^H, C^21^H), 5.49 (br. s, 1H, OH), 5.23 (br. s, 1H, C^1′^H), 4.69 (br. s, 1H, OH), 4.07 (br. s, 4H, C^15^H_3_, C^5′^H), 3.84, 3.76, 3.75 (s, all three 3H, C^23^H_3_, C^24^H_3_, C^25^H_3_), 3.74–3.67 (m, 3H, C^7^H, C^4′^H, C^16^H_eq_), 3.61 (d, 1H, *^2^J_HH_* = 14.4, C^16^H_ax_), 3.13 and 2.84 (d, both 1H, *^2^J_HH_* = 18.8, C^10^H_2_), 2.95–2.90 (m, 1H, C^3′^H), 2.42 (s, 3H, C^14^H_3_), 2.37 (d, 1H, *^2^J_HH_* = 15.0, C^8^H_eq_), 2.09 (dd, 1H, *^2^J_HH_* = 14.6, *^3^J_HH_* = 4.0, C^8^H_ax_), 1.80 (dt, 1H, *^2^J_HH_* = 12.7, *^3^J_HH_* = 3.8, C^2′^H_eq_), 1.63 (dd, 1H, *^2^J_HH_* = 13.1, *^3^J_HH_* = 4.8, C^2′^H_ax_), 1.41 (d, 3H, *^3^J_HH_* = 6.4, C^6′^H_3_) ppm; ^13^C{^1^H}NMR (100 MHz; CDCl_3_; Me_4_Si): δ 211.68 (C^13^), 186.62, 186.24 (C^5^, C^12^), 160.74 (C^4^), 156.18, 155.54 (C^6^, C^11^), 151.56 (C^22^), 148.65 (C^19^), 142.51 (C^20^), 135.47 (C^2^), 135.15 (C^1a^), 134.09 (C^6a^, C^11a^), 120.49 (C^4a^), 119.51 (C^17^), 118.51, 118.17 (C^1^, C^3^), 113.87 (C^18^), 111.04, 110.89 (C^5a^, C^12a^), 100.90 (C^21^), 97.06 (C^1′^), 76.64 (C^9^), 69.70, 68.01, 66.34 (C^7^, C^4′^, C^5′^), 55.79, 55.93, 56.35 (C^15^, C^23^–C^25^), 51.53 (C^3′^), 44.71 (C^16^), 34.66, 33.01, 30.07 (C^8^, C^10^, C^2′^), 24.61 (C^14^), 16.98 (C^6′^) ppm; HPLC-MS (DUIS) *m/z* 708.45 ([M + H]^+^, 100%), 1416.80 ([2M + 2H]^+^, 6%); Retention time = 4.34; HRMS (ESI) *m/z* calc’d C_37_H_41_NO_13_ [M + H]^+^ 708.2651, found 708.2632.

(*8S*,*10S*)-8-Acetyl-6,8,11-trihydroxy-10-([(*2R*,*4S*,*5S*,*6S*)-5-hydroxy-6-methyl-4-((2,5-dimethoxybenzyl)amino)tetrahydro-*2H*-pyran-2-yl]oxy)-1-methoxy-7,8,9,10-tetrahydrotetracene-5,12-dione (**4g**). The daunorubicin derivative **4g** was obtained previously using the same method [53].
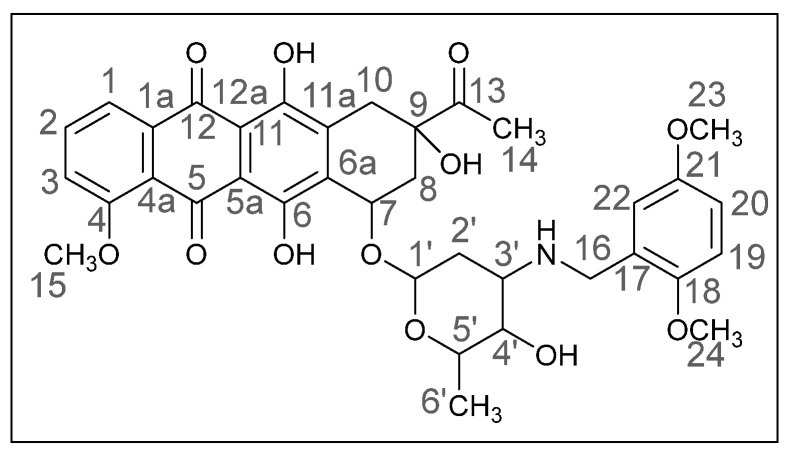


Dark red powder [3] (0.15 g, 47%); mp: (decomp.) > 200 °C; IR (KBr, cm^−1^): 3475brm (OH), 2935m and 2836m (both CH), 1717m (C=O), 1618s and 1579s (both C=C), 1500s, 1445s and 1414s (both OH), 1379m, 1352m, 1286vs, 1230s, 1210s (C–O), 1122s, 1084m, 1069m, 1034s, 987vw (C=C), 874m, 796m, 763w, 464w; ^1^H NMR (400 MHz; CDCl_3_; Me_4_Si): δ 13.95 (br. s, 2H, C^6^OH, C^11^OH), 8.01 (d, 1H, *^3^J_HH_* = 7.8, C^1^H), 7.89 (t, 1H, *^3^J_HH_* = 8.0, C^2^H), 7.40 (d, 1H, *^3^J_HH_* = 8.0, C^3^H), 6.80–6.72 (m, 3H, C^19^H, C^20^H, C^22^H), 5.52 (br. s, 1H, OH), 5.27 (br. s, 1H, C^1’^H), 4.69 (br. s, 1H, OH), 4.09 (br. s, 5H, C^15^H_3_, C^5’^H, C^7^H), 3.80–3.67 (m, 3H, C^16^H_2_, C^4’^H), 3.74 and 3.71 (s, both 3H, C^23^H_3_, C^24^H_3_), 3.20 and 2.92 (d, both 1H, *^2^J_HH_* = 19.0, C^10^H_2_), 2.99 (br. s, 1H, C^3’^H), 2.44 (s, 3H, C^14^H_3_), 2.37 and 2.14 (d, both 1H, *^2^J_HH_* = 15.0, C^8^H_2_), 1.88–1.80 and 1.69–1.66 (m, both 1H, C^2’^H_2_), 1.41 (d, 3H, *^3^J_HH_* = 5.6, C^6’^H_3_) ppm; ^13^C{^1^H}NMR (100 MHz; CDCl_3_; Me_4_Si): δ 211.77 (C^13^), 186.52, 186.19 (C^5^, C^12^), 160.73 (C^4^), 156.16, 155.49 (C^6^, C^11^), 153.22, 151.47 (C^18^, C^21^), 135.44 (C^2^), 135.11 (C^1a^), 134.16, 134.08 (C^6a^, C^11a^), 127.71 (C^17^), 120.47 (C^4a^), 119.49, 118.18 (C^1^, C^3^), 115.91 (C^22^), 112.65, 111.01 (C^19^, C^20^), 110.97, 110.85 (C^5a^, C^12a^), 100.82 (C^1’^), 76.61 (C^9^), 69.64 (C^7^), 66.43, 66.27 (C^4’^, C^5’^), 56.42 (C^15^), 55.46 (C^23^, C^24^), 51.71 (C^3’^), 45.07 (C^16^), 34.67, 32.98, 29.88 (C^8^, C^10^, C^2’^), 24.63 (C^14^), 16.95 (C^6’^) ppm; HPLC-MS (DUIS) *m/z* 678.50 ([M + H]^+^, 61%); Retention time = 5.07.

(*8S*,*10S*)-8-Acetyl-6,8,11-trihydroxy-10-([(*2R*,*4S*,*5S*,*6S*)-5-hydroxy-6-methyl-4-(((3,4,5-trimethoxybenzyl)amino)tetrahydro-*2H*-pyran-2-yl]oxy)-1-methoxy-7,8,9,10-tetrahydrotetracene-5,12-dione (**4h**). Daunorubicin derivative **4h** was obtained previously using the same method [53].
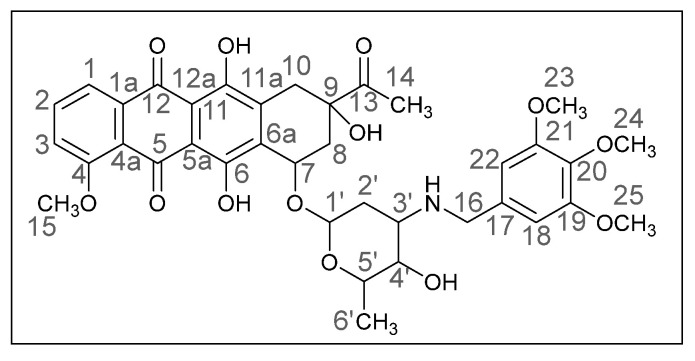


Red powder [3] (0.18 g, 63%); mp: (decomp.) > 200 °C; IR (KBr, cm^−1^): 3481brm (OH), 2969m, 2936m and 2839m (three CH), 1717m (C=O), 1617s and 1588s (both C=C), 1458s, 1447shs and 1416s (both OH), 1379m, 1352m, 1287s, 1233s and 1209s (both C–O), 1127s, 1069m, 1034s, 989vs (C=C), 820m, 793brm, 764m, 464m; ^1^H NMR (400 MHz; CDCl_3_; Me_4_Si): δ 13.90 (br. s, 2H, C^6^OH, C^11^OH), 7.95 (d, 1H, *^3^J_HH_* = 7.8, C^1^H), 7.75 (t, 1H, *^3^J_HH_* = 8.0, C^2^H), 7.37 (d, 1H, *^3^J_HH_* = 7.8, C^3^H), 6.47 (br. s, 2H, C^18^H, C^22^H), 5.50 (br. s, 1H, OH), 5.23 (br. s, 1H, C^1′^H), 4.61 (br. s, 1H, OH), 4.06 (br. s, 4H, C^15^H_3_, C^5′^H), 3.85 (br. s, 2H, C^7^H, C^4′^H), 3.77 (s, 9H, C^23^H_3_, C^24^H_3_, C^25^H_3_), 3.71 и 3.62 (d, both 1H, *^2^J_HH_* = 12.6, C^16^H_2_), 3.11 and 2.80 (d, both 1H, *^2^J_HH_* = 18.0, C^10^H_2_), 3.00–2.95 (m, 1H, C^3′^H), 2.41 (s, 3H, C^14^H_3_), 2.35 and 2.10 (dd, both 1H, *^2^J_HH_* = 12.0, *^3^J_HH_* = 4.2, C^8^H_2_), 1.84 (dt, 1H, *^2^J_HH_* = 13.2, *^3^J_HH_* = 4.0, C^2′^H_eq_), 1.74 (dd, 1H, *^2^J_HH_* = 12.9, *^3^J_HH_* = 4.9, C^2′^H_ax_), 1.37 (d, 3H, *^3^J_HH_* = 6.5, C^6′^H_3_) ppm; ^13^C{^1^H} NMR (100 MHz; CDCl_3_; Me_4_Si): δ 211.63 (C^13^), 186.67, 186.30 (C^5^, C^12^), 160.83 (C^4^), 156.22, 155.56 (C^6^, C^11^), 153.15 (C^20^), 135.60 (C^2^), 135.19 (C^1a^), 134.69 (C^19^, C^21^), 134.24 (C^6a^, C^11a^), 134.10 (C^17^), 120.54 (C^4a^), 119.62, 118.32 (C^1^, C^3^), 111.12, 110.97 (C^5a^, C^12a^), 104.99 (C^18^, C^22^), 100.84 (C^1′^), 76.66 (C^9^), 69.80, 66.88, 66.63 (C^7^, C^4′^, C^5′^), 60.55 (C^15^), 56.50 (C^24^), 55.90 (C^23^, C^25^), 52.49 (C^3′^), 50.49 (C^16^), 34.71, 33.09, 30.04 (C^8^, C^10^, C^2′^), 24.63 (C^14^), 17.00 (C^6′^) ppm; HPLC-MS (DUIS) *m/z* 708.45 ([M + H]^+^, 53%); Retention time = 5.20.

#### 3.2.1. X–Ray Diffraction Data

A saturated solution of the anthracycline derivative **4d** was prepared in a mixture of toluene–acetone = 1:2. A five-fold volume of MTBE, saturated with hydrogen chloride, was added to the red solution and filtered into a test tube without mixing. The tube was loosely sealed and kept at room temperature for 2 weeks until cubic single crystals formed.

Using Olex2 [54], the structures were solved with the ShelXT [55] structure solution program using intrinsic phasing and refined with the XL refinement package [56] using a least squares minimization against *F^2^* in anisotropic approximation for non-hydrogen atoms. Hydrogen atoms of NH and OH groups were found in the differential Fourier synthesis while the positions of other hydrogen atoms were calculated, and they all were refined in the isotropic approximation within the riding model.

#### 3.2.2. Cell Cultivation

Cell lines were provided by the Institute of Cytology, Russian Academy of Sciences (St. Petersburg, Russia). Human cell cultures A549 (ATCC^®^ CCL-185™), RD (ATCC^®^ CC-136™), HCT116 (ATCC^®^ CCL-247™) and HEK293 (ATCC^®^ CRL-1573™) were grown in DMEM (NLP PanEko, Moscow, Russia); cell culture MCF7 (ATCC^®^ HTB-22™) was grown in EMEM (NLP PanEko); and cell culture Jurkat (ATCC^®^ HIB-152™) was grown in RPMI-1640 (NLP PanEko). To growth medium, 10% fetal calf serum (HyClone^®^, Thermo Scientific, Waltham, MA, USA), 2 mmol L-glutamine (NLP PanEco), and 1% gentamicin (JSC Biochemist, Moscow, Russia) were added as antibiotics and incubated at 37 °C in an atmosphere of 5% CO_2_ and 95% air.

#### 3.2.3. Cytotoxicity

The cytotoxicity of the compounds was determined by the MTT test method [57]. Cells A549, RD, HCT116, MCF7, and HEK293 were seeded in a 96-well plate (Costar^®^) in 1 × 10^4^ cells/200 μL and cultured at 37 °C in a humid environment containing 5% CO_2_. After 24 h of incubation, solutions of different concentrations (from 100 to 0.0012 μM) were added to the cell culture, and then the cells were cultured under the same conditions for 72 h. For each concentration, the experiments were performed in triplicate. All compounds were dissolved in DMSO (PANREAC QUIMICA S.L.U). The final concentration of DMSO in the well did not exceed 0.1% and was not toxic to the cells. The control wells were added to the solvent in an amount of 0.1%. After incubation, 20 μL of a solution of 5 mg/mL of MTT [bromide 3-(4,5-dimethylthiazol-2-yl)-2,5-diphenyltetrazolium] (Sigma-Aldrich, St. Louis, MO, USA) in PBS (phosphate-buffered saline) and additionally incubated for 2 h. Then, the medium was removed, and 100 μL DMSO was added to each well to dissolve formed formazan crystals. Using a BioTek Instruments Cytation 3 Imager plate analyzer, the optical density at 536 nm was determined. The concentration value, which causes a 50% inhibition of cell population growth (IC_50_), was estimated on the basis of dose-dependent curves using GraphPad Prism 7 software.

The cytotoxicity of the compounds **4e** and **4f** was determined by the Alamar Blue test [57]. The Jurkat cells were seeded in a 96-well plates in a concentration of 7 × 10^4^ cells/200 μL. The tested compounds (10–0.0012 μM) were added into each well, and these cells were incubated under similar conditions for 72 h. The experiments were carried out in triplicate. All the substances were dissolved in DMSO. The final DMSO concentration in one well was less than or equal to 0.1% and was nontoxic for the cells. The solvent in the final concentration of 0.1% was added to the control wells. After the incubation with the compounds, resazurin (7-hydroxy-3H-phenoxazine-3-on-10-oxide sodium salt, 22 μL per one well) (Sigma-Aldrich) with a final concentration of 50 μM was added to each well. The plates were incubated for 2 h. The fluorescence of the reduced dye was determined using a Cytation3 (BioTek Instruments, Inc., Winooski, VT, USA) microplate reader with excitation at 530 nm and emission at 590 nm. The concentration that caused the 50% inhibition of the growth of the cell population (IC_50_) was determined from the dose-dependent curves using GraphPad Prism 7 software.

#### 3.2.4. Cell Cycle

Evaluation of the effect on the cell cycle was determined using flow cytometry by measuring the DNA content. The cells were seeded in a 12-well plate (0.5 × 10^6^ cells in 1000 μL), and solutions of the test compounds were added (final concentrations are shown in the Results and Discussion sections) then incubated for 24, 48, and 72 h. Cell material was prepared according to the reagent manufacturer’s instructions; cells were fixed with cold ethanol (70%) for 15 min and washed with PBS. They were then analyzed with an Attune NxT Acoustic Focusing Cytometer (Thermo Fisher Scientific, USA) using a 405 nm laser with a 440/50 bandpass filter, after reaching 50,000 events with a standard flow rate of 12.5 μL/min.

#### 3.2.5. Glycolysis

Cell glycolysis was determined using a Seahorse Bioscience XF96 Extracellular Flux Analyzer with Seahorse XF Glycolysis Stress Test Kit (Agilent Technology, Santa Clara, CA, USA). A total of 40.000 cells per well were seeded in 96-well cell culture XF microplates (Agilent Technology, Santa Clara, CA, USA) and incubated for 24 h. ECAR levels were examined. Cells were kept in XF basal medium (pH 7.4) containing 1 mM glutamine after sequential addition of glucose (10 mM), oligomycin (1 μM) and 2-DG (50 mM). The drugs were sequentially added into wells of XF microplates as indicated.

#### 3.2.6. Analysis of Hexokinase, Phosphofructokinase, and Pyruvate Kinase Activity

To study the activity of key glycolytic enzymes, a colorimetric assay was performed using commercially available kits (#MAK091, MAK072, MAK093, Sigma). The sample preparation process included lysis on ice of 1 × 10^6^ lung carcinoma A549 cells pretreated with the test compounds at a concentration of 1 μM for 90 min (the time equivalent to the glycolysis procedure) in NP-40 buffer containing 2 mM DTT and protease inhibitors, followed by clarification by centrifugation at 12,000 rpm. All subsequent procedures were carried out according to the manufacturer’s instructions. Results are presented as mean ± SEM (n = 5).

#### 3.2.7. Acute Toxicity In Vivo

The acute toxicity of compounds 4e and 4f was determined by the method of V.B. Prozorovsky in CD-1 white male mice weighing 22–24 g using the intraperitoneal route of administration [58].

#### 3.2.8. Molecular Modeling

The structure of the self-complementary DNA duplex (ACGTACGT)_2_ was obtained from the Protein Data Bank (PDB: 1AL9) [59]. The ligand structures were converted to three dimensions and preoptimized in the MMFF94 force field using Avogadro 1.2.0 software [59], and then the ligand and DNA structures (upon removal of the bound bis-daunorubicin ligand) were prepared for molecular docking using AutoDock Tools 1.5.6. [60]. Molecular docking into the intercalator binding sites was performed with AutoDock Vina 1.1.2 software [61] (grid box size 18 Å × 18 Å × 19.5 Å, exhaustiveness = 16) or (if necessary) manually using USCF Chimera 1.15 software [62]. The unmodified daunorubicin molecule was docked into one of the symmetrical binding sites with the sugar residue oriented outwards the double helix. This allowed us to stabilize the complex structure and avoid potential artifacts caused by the interaction between the ligands bound in the two sites. The compounds under study were then docked into the second binding site in the approximately correct pose. The poses with the best scoring function values and ligand positions were selected, and the complex model was built using the USCF Chimera 1.15 software.

The molecular dynamics simulations were performed using the CHARMM36/CGenFF 4.4 force field [63,64] in GROMACS 2021.2 software [65]. The initial models of the systems were built using the Ligand Reader and Modeler and Solution Builder modules of the CHARMM-GUI web service [66]. The complex was inserted into a rectangular periodic boundary box of water in the TIP3P model; the distance from the DNA molecules to the box border was no less than 10 Å. Individual randomly selected water molecules were replaced with potassium and chlorine ions to ensure the electrical neutrality of the system and a total KCl concentration of about 0.15 M. For each system, the molecular mechanics minimization (up to 5000 steps) was performed on the CPU and followed by equilibration for 125 ps with an integration timestep of 1 fs at a temperature of 300 K and a constant volume using the v-rescale thermostat on an NVIDIA GeForce RTX 3080 GPU. The production simulation was performed on the GPU with an integration timestep of 2 fs at a temperature of 300 K and a constant pressure of 1 atm using the v-rescale thermostat and a Parrinello–Rahman barostat. The hydrogen atoms’ movements were constrained using the LINCS algorithm. In addition, flat-bottom potential restraints were imposed on the distances between the terminal base pairs to prevent duplex unwinding due to the edge effects. For analysis and visualization of the results, cpptraj software [67] in the AmberTools 21 package [68] and UCSF Chimera were used.

#### 3.2.9. In Silico Drug–Likeness Evaluation

ADME descriptors were obtained using SwissADME software from the Swiss Institute of Bioinformatics [69].

## 4. Conclusions

In this paper, we present a very simple and effective method for the structural modification of daunorubicin by introducing polymethoxybenzyl substituents into its carbohydrate fragment. Using the well-established method of reductive amination, a series of new daunorubicin derivatives with high cytotoxicity were obtained. 

When studying the cytotoxicity of the synthesized compounds, we found that the modified daunorubicin analogues had a more pronounced effect on some cell lines than daunorubicin. Two synthesized compounds exceeded the cytotoxicity of daunorubicin by 100 times. Cytotoxicity is affected by the arrangement of methoxy groups in the polyalkoxybenzene substituent; compounds with a combination of ortho- and para-located methoxy groups will thus have the maximum activity. 

**Figure 10:**
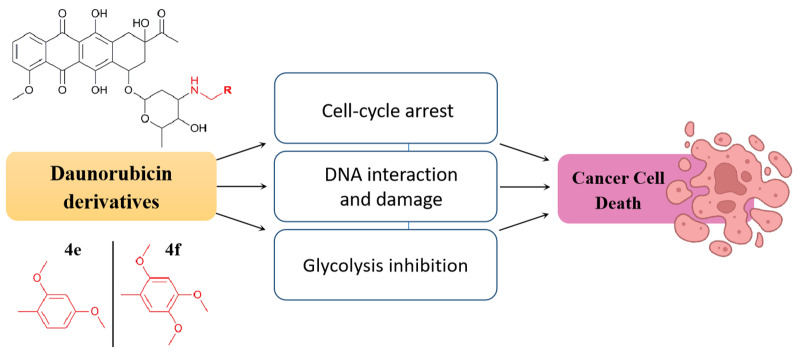
Schematic representation of the possible mechanisms of the cytotoxic action of daunorubicin derivatives.

The mechanisms of action of the substances can be considered through the influence of substances on the cell cycle. Unlike daunorubicin, which causes cell cycle arrest in the G2/M phase, the most cytotoxic compounds **4e** and **4f** caused cell accumulation in the G0/G1 phase. Moreover, the most active substances were found to inhibit glycolysis, which leads to disruption of the metabolic activity of the tumor cell and its subsequent death. Moreover, the ability of the synthesized derivatives to interact with DNA can also be considered mechanism of cytotoxicity. Using molecular modeling, it was found that only two of the most active compounds had a portion of methoxy groups oriented toward the bases in the minor groove of DNA, which probably enhanced their topoisomerase activity. Figure 10 shows a schematic representation of the summarized information on the possible mechanisms of cytotoxicity of the new daunorubicin derivatives.

Another feature of these compounds is their lower toxicity in vivo compared to daunorubicin.

The obtained results allow us to consider newly synthesized daunorubicin derivatives as a basis for the creation of potential antitumor agents with high cytotoxicity towards cancer cells and reduced systemic toxicity in vivo.

## Figures and Tables

**Table 1:** IC_50_ values of the tested compounds against the cell lines in the cited MTT assay.

	IC_50_ (μM)				
	A549	RD	HCT116	MCF7	HEK293
**DR** *	0.46 ± 0.01 *(SI < 10)*	0.30 ± 0.01 *(SI = 12.00)*	0.21 ± 0.00 *(SI = 17.14)*	1.80 ± 0.17 *(SI < 10)*	3.60 ± 0.35
**4** **a**	0.17 ± 0.00 *(SI = 25.71)*	0.31 ± 0.02 *(SI = 14.10)*	0.03 ± 0.00 *(SI > 50)*	0.13 ± 0.04 *(SI = 33.62)*	4.37 ± 0.00
**4b**	0.26 ± 0.01 *(SI < 10)*	0.45 ± 0.01 *(SI < 10)*	0.45 ± 0.00 *(SI < 10)*	0.16 ± 0.00 *(SI < 10)*	0.94 ± 0.01
**4c**	42.12 ± 5.25 *(SI < 10)*	39.25 ± 2.35 *(SI < 10)*	80.10 ± 1.02 *(SI < 10)*	79.45 ± 4.77 *(SI < 10)*	87.50 ± 6.69
**4d**	1.86 ± 0.13 *(SI < 10)*	1.43 ± 0.06 *(SI < 10)*	1.73 ± 0.02 *(SI < 10)*	1.66 ± 0.23 *(SI < 10)*	0.52 ± 0.06
**4e**	0.001 ± 0.00 *(SI > 50)*	0.08 ± 0.01 *(SI < 10)*	0.04 ± 0.00 *(SI < 10)*	0.085 ± 0.01 *(SI < 10)*	0.05 ± 0.01
**4f**	0.001 ± 0.01 *(SI > 50)*	0.05 ± 0.01 *(SI < 10)*	0.21 ± 0.01 *(SI < 10)*	0.23 ± 0.02 *(SI < 10)*	0.23 ± 0.01
**4g**	0.21 ± 0.01 *(SI = 12.62)*	0.10 ± 0.03 *(SI = 26.50)*	0.13 ± 0.05 *(SI = 20.38)*	0.57 ± 0.02 *(SI < 10)*	2.65 ± 0.14
**4h**	0.36 ± 0.00 *(SI = 32.64)*	1.29 ± 0.06 *(SI < 10)*	0.007 ± 0.00 *(SI > 50)*	0.45 ± 0.02 *(SI = 26.11)*	11.75 ± 0.30

*—daunorubicin.

**Table 2:** Parameters of glycolytic function of the A549 cell line under the action of the studied compounds.

	Glycolysis	Glycolytic Capacity	Glycolytic Reserve
**Control**	19.70 ± 0.97	53.06 ± 1.58	33.36 ± 1.73
**4** **a**	6.02 ± 0.04 ****	27.16 ± 0.46 ****	21.14 ± 0.13 ****
**4b**	15.01 ± 1.03 *	41.43 ± 1.40 ***	26.42 ± 0.62 **
**4c**	19.28 ± 0.93	52.29 ± 1.90	33.01 ± 1.10
**4d**	17.33 ± 1.45	47.50 ± 0.49 *	30.17 ± 0.98
**4e**	6.50 ± 0.11 ****	22.66 ± 0.19 ****	16.16 ± 0.23 ****
**4f**	5.58 ± 0.10 ****	20.80 ± 0.60 ****	15.22 ± 0.42 ****
**4g**	6.83 ± 0.24 ****	27.18 ± 0.43 ****	20.35 ± 0.28 ****
**4h**	15.07 ± 0.54 *	45.27 ± 0.15 **	29.20 ± 0.28 *
**DR**	14.32 ± 1.66 **	39.27 ± 1.01 ****	24.95 ± 2.84 **

The data are represented as mean ± SEM, *n* = 3. *, **, ***, ****–*p* < 0.05, *p* < 0.01, *p* < 0.001, *p* < 0.0001 by one-way ANOVA with Dunnett’s test.

**Table 3:** Drug-likeness analysis of compounds.

Cmpd.	Physicochemical Properties (Lipinski Rule of Five)					Solubility			Pharmacokinetics	
	MW	HB Donors	HB Acceptors	Rotatable Bonds	Consensus Log P	Log S (ESOL)	Log S (Ali)	Log S (SILICOS-IT)	GI Absorption	CYP Enzyme Inhibitors
**4e**	662	5	12	8	3.02	Poorly soluble			Low	No
**4f**	708	5	14	10	2.68	Poorly soluble			Low	No
**DR**	528	5	11	4	1.12	Moderately soluble			Low	No

## Data Availability

The original contributions presented in this study are included in the article/supplementary material. Further inquiries can be directed to the corresponding authors.

## Supplementary Materials

The following supporting information can be downloaded at: https://www.mdpi.com/article/10.3390/ijms26031270/s1.
