# Supplementary material for: Design of New Daunorubicin Derivatives with High Cytotoxic Potential
Source: Int J Mol Sci. 2025 Jan 31;26(3):1270. doi: 10.3390/ijms26031270 (PMC11818560; doi:10.3390/ijms26031270)
Supplement: Supplementary file 1 [file ijms-26-01270-s001.zip › ijms-3394117-supplementary.pdf]

Design of new daunorubicin derivatives with high cytotoxic potential

Aleksandra A. Kalashnikova[1,†], Altynkul B. Toibazarova[2,†], Oleg I. Artyushin[1], Lada V. Anikina[3], Anastasiya A. Globa[3], Zinaida S. Klemenkova[1], Maxim V. Andreev[1], Eugene V. Radchenko[4], Vladimir A. Palyulin[4], Yulia R. Aleksandrova[1], Marat I. Syzdykbayev[5], Nurbol O. Appazov[6,7], Vladimir N. Chubarev[8], Margarita E. Neganova[1,*] and Valery K. Brel[1,*]

1 Nesmeyanov Institute of Organoelement Compounds, Russian Academy of Sciences, Vavilova St. 28, bld. 1, Moscow, 119991, Russia
2 Laboratory of Engineering Profile,Korkyt Ata Kyzylorda University, Ayteke bi Str., 29A, Kyzylorda 120014, Kazakhstan
3 Institute of Physiologically Active Compounds of the FSBIS of the Federal Research Center for Problems of Chemical Physics and Medicinal Chemistry of the RAS, 1 Severnyi Proezd, Chernogolovka, 142432, Russia
4 Department of Chemistry, Lomonosov Moscow State University, Leninskie Gory, Moscow, 119991, Russia
5 Department Biology, Geography and Chemistry; Laboratory of Engineering Profile, Korkyt Ata Kyzylorda University, Ayteke bi Str., 29A, Kyzylorda 120014, Kazakhstan
6 Laboratory of Engineering Profile; Department of Engineering Technology, Korkyt Ata Kyzylorda University, Ayteke bi Str., 29A, Kyzylorda 120014, Kazakhstan
7 "CNEC" LLP, Dariger Ali Lane, 2, Kyzylorda 120001, Kazakhstan
8 Sechenov University, Trubetskaya St., 8-2, Moscow, 119991, Russia
* Correspondence: v_brel@mail.ru(V.B.); neganovam@ineos.ac.ru(M.N.); ; Tel.: +7-499-135-61-66, (V.B.); +7-499-135-61-66 (M.N.)
† These authors contributed equally to this work.

† - Contributed equally
* Correspondence: v_brel@mail.ru(VB); Tel.: +7-499-135-61-66, neganovam@ineos.ac.ru(ME); Tel.: +7-499-135-61-66

Supplementary materials

List of content:

1. X-ray diffraction data S2

2. Molecular modeling S3

3. Cell cycle S4

4. NMR spectra of all compounds S5-S12

5. MS spectra of all compounds S13-S34

6. IR spectra of all compounds S35-S38

X-ray diffraction data

Crystal data and structure refinement parameters are given in Table 1. CCDC 2174639 contain the supplementary crystallographic data for of **4d**.

Table 1. Crystal data and structure refinement parameters for **4d**.

Parameter	**4d**
Formula unit	$C_{40.12}H_{48.66}ClNO_{14.12}$
Formula weight	806.41
Temperature, K	100
Crystal system	Orthorhombic
Space group	$P2_12_12_1$
Z	8
a, Å	12.5828(5)
b, Å	25.1898(9)
c, Å	25.4948(10)
α, °	90
β, °	90
γ, °	90
V, $Å^3$	8080.8(5)
D_{calc} (g cm^{-3})	1.326
Linear absorption, μ (cm^{-1})	1.63
F(000)	3411
$2\Theta_{max}$, °	50
Reflections measured	110069
Independent reflections	14209
Observed reflections [$I > 2\sigma(I)$]	12122
Parameters	1128
R1	0.0453
wR2	0.1196
GOF	1.073
$\Delta\rho_{max}/\Delta\rho_{min}$ (e $Å^{-3}$)	0.671 / -0.345

Molecular modeling

The plots of the root mean square deviations (RMSD) of the DNA and ligand non-hydrogen atoms (Fig. S1) as well as the visual inspection of the trajectories confirm that the stability of the systems and the basic binding mode of the ligands are retained throughout the entire course of the production simulation (200 ns).

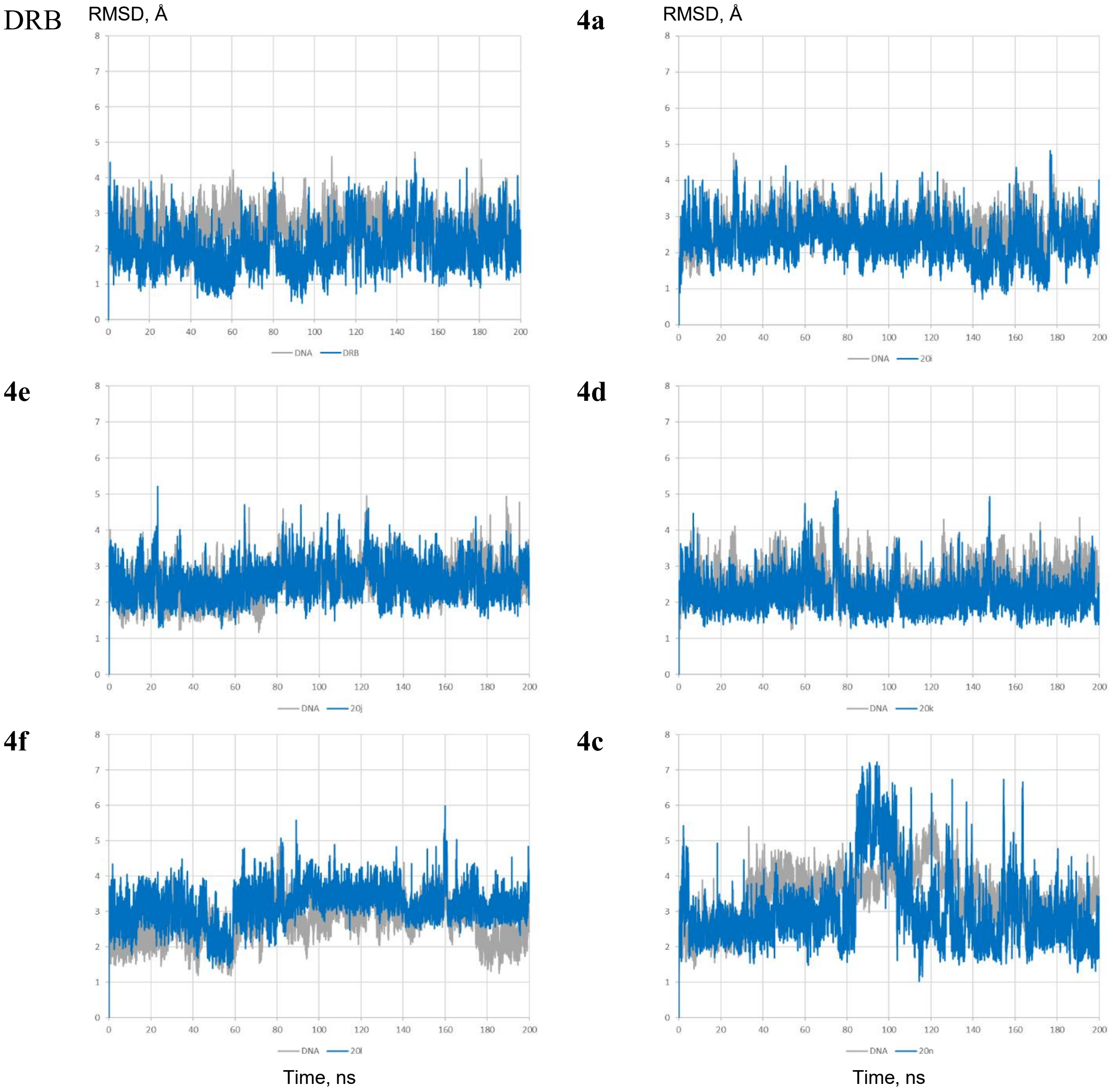

Figure S1. RMSD of the DNA and ligand non-hydrogen atoms during molecular dynamics simulation of their complexes.

Cell cycle

In contrast to daunorubicin, which induces cell cycle arrest in the G2/M phase, compounds **4e** and **4f** causes cell accumulation in the G0/G1 phase, which needs to be clarified in further studies.

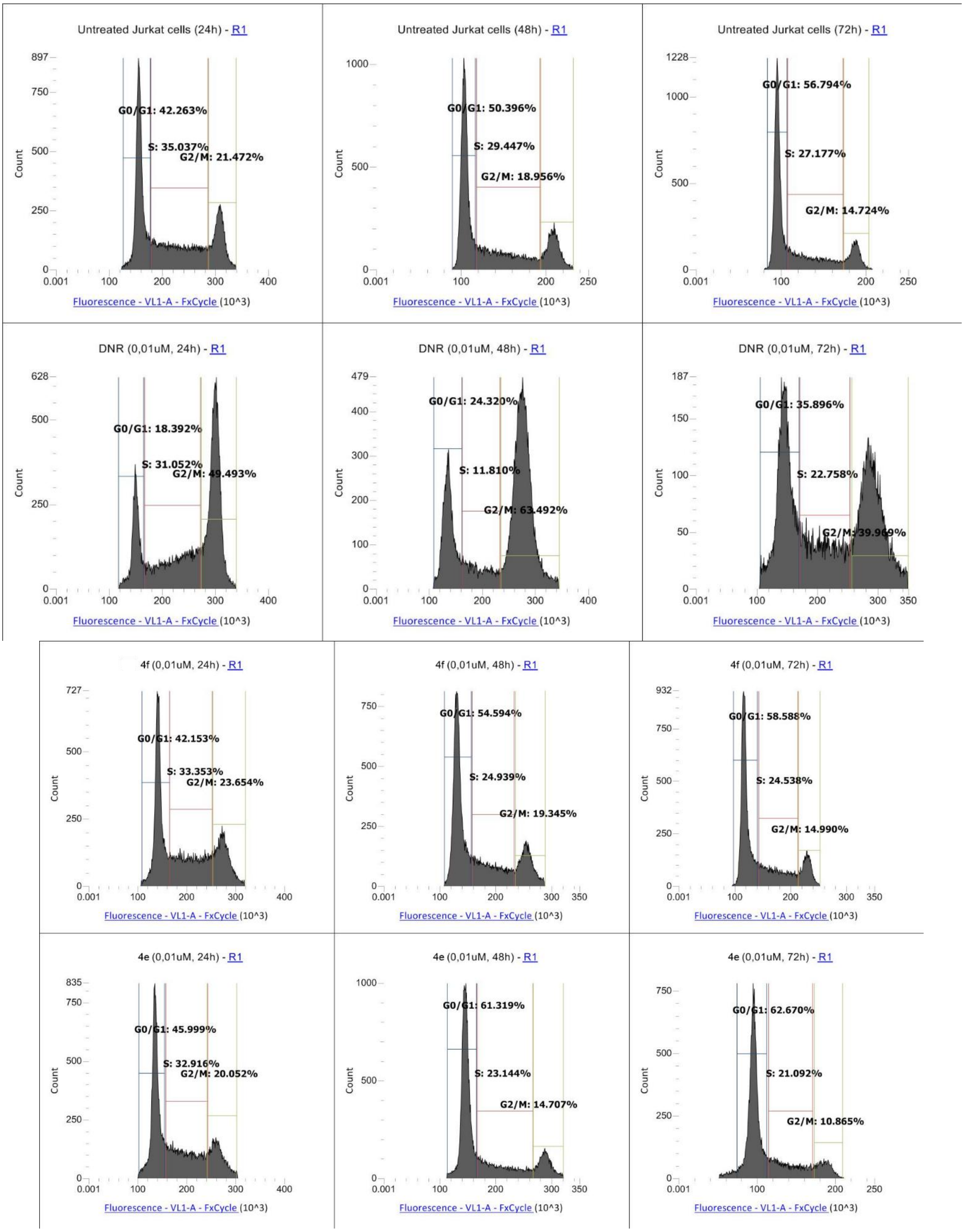

Figure S2. Histograms of the Jurkat cell line showing the distribution of DNA content after exposure to the compounds after 24, 48 and 72 hours.

NMR spectra of all compounds

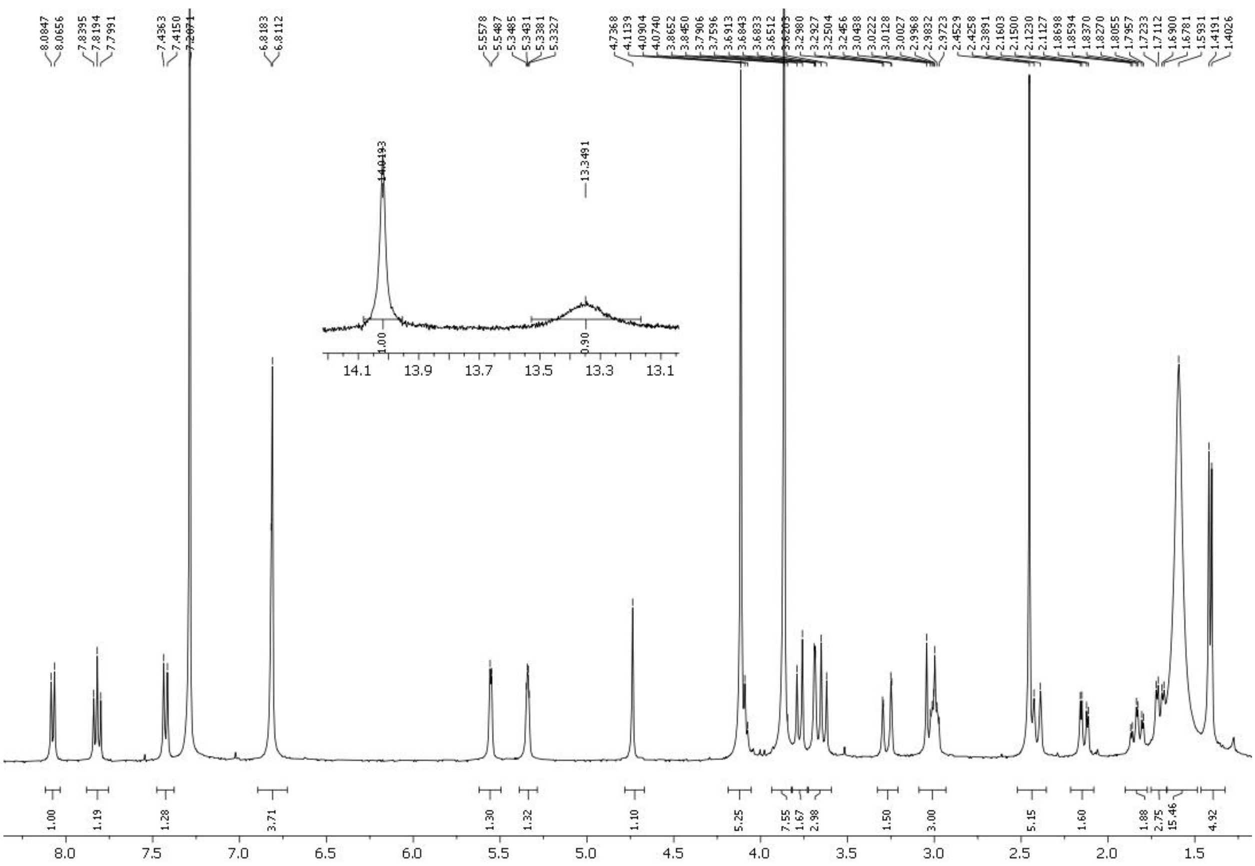

Figure S3. ^{1}H NMR spectrum of amine **4a** (400 MHz, $CDCl_3$).

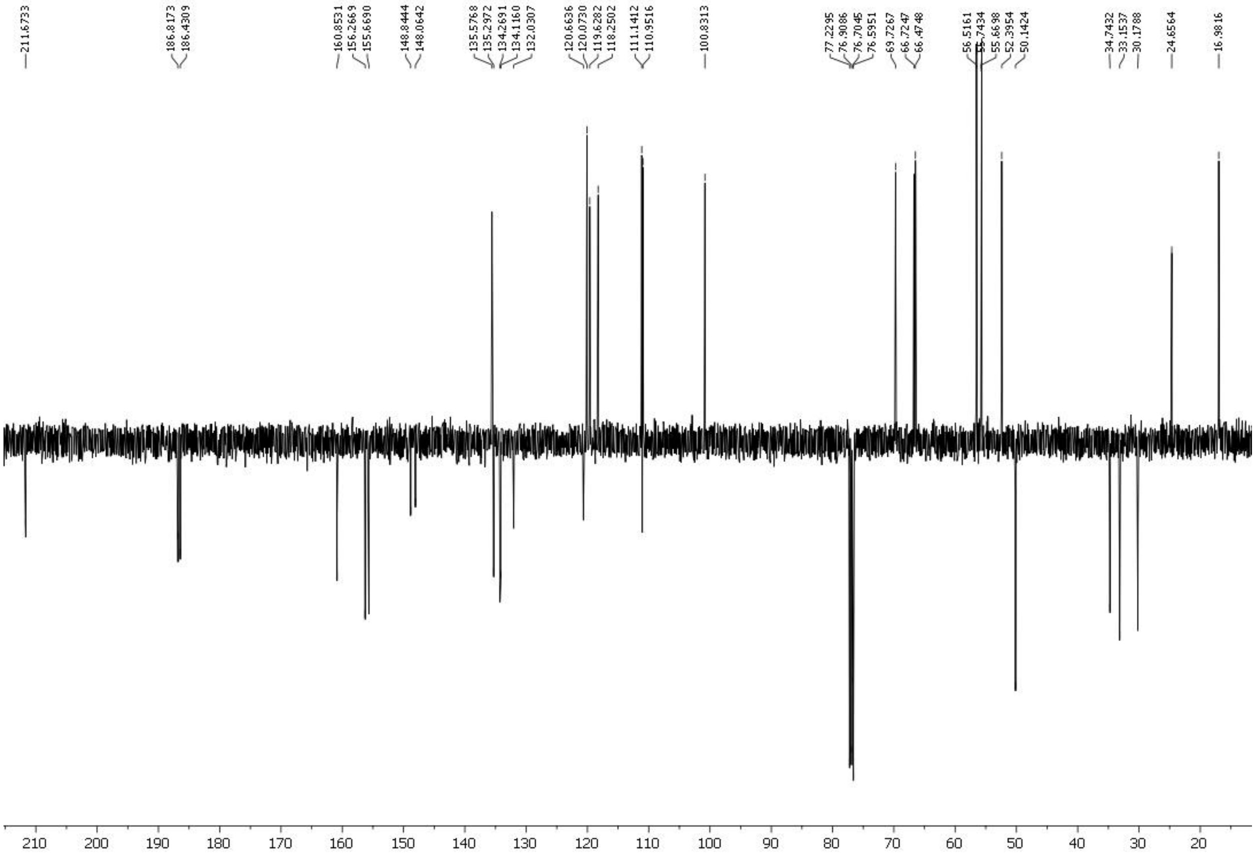

Figure S4. ^{13}C NMR spectrum of amine **4a** (100 MHz, $CDCl_3$).

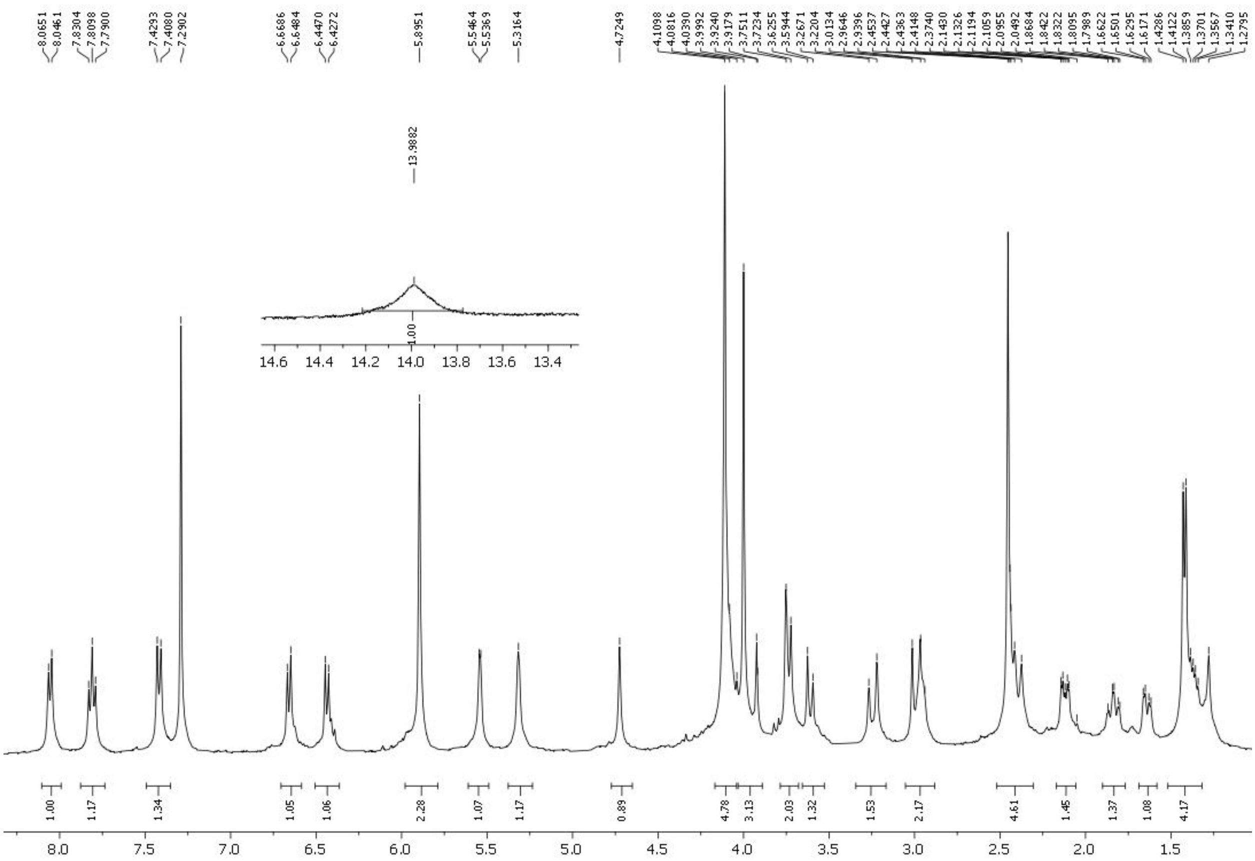

Figure S5. ^{1}H NMR spectrum of amine **4b** (400 MHz, $CDCl_3$).

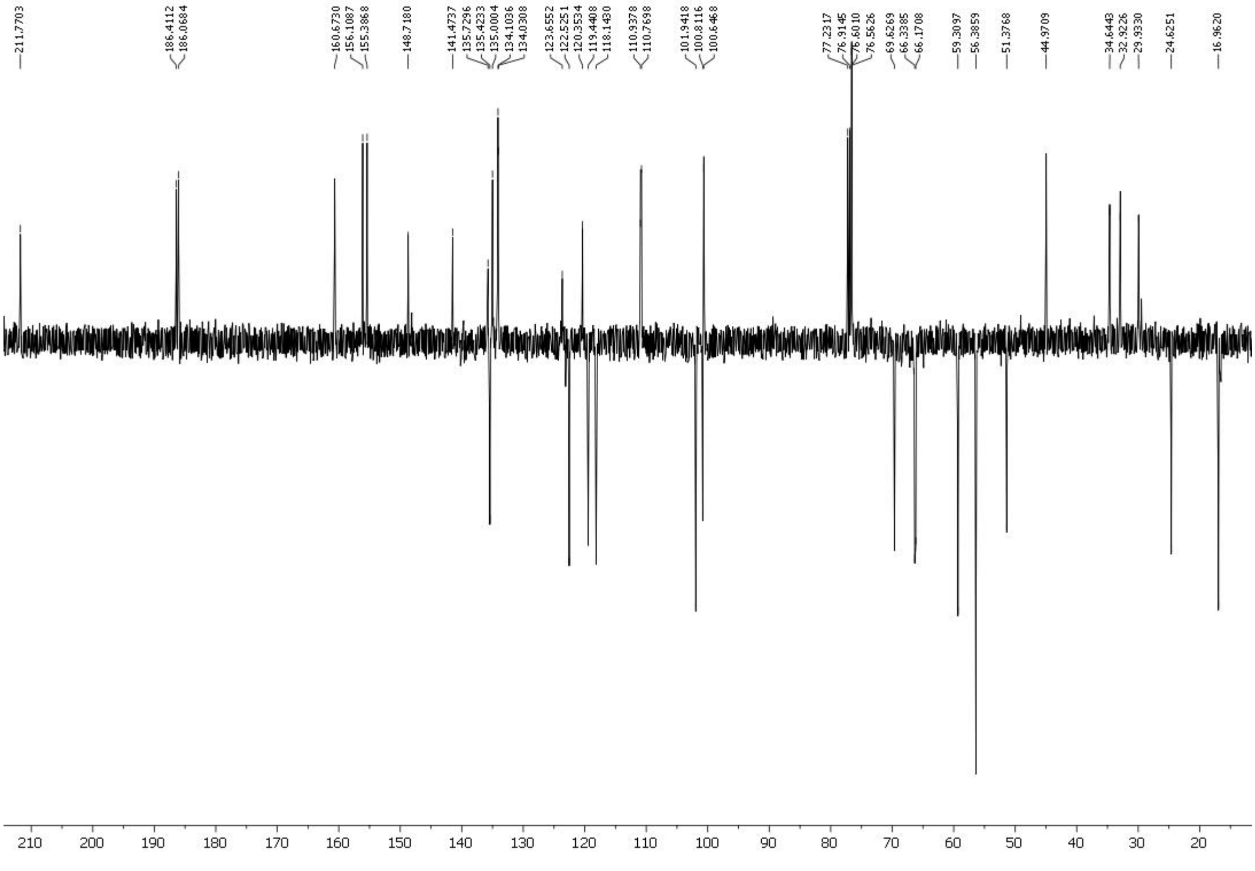

Figure S6. ^{13}C NMR spectrum of amine **4b** (100 MHz, $CDCl_3$).

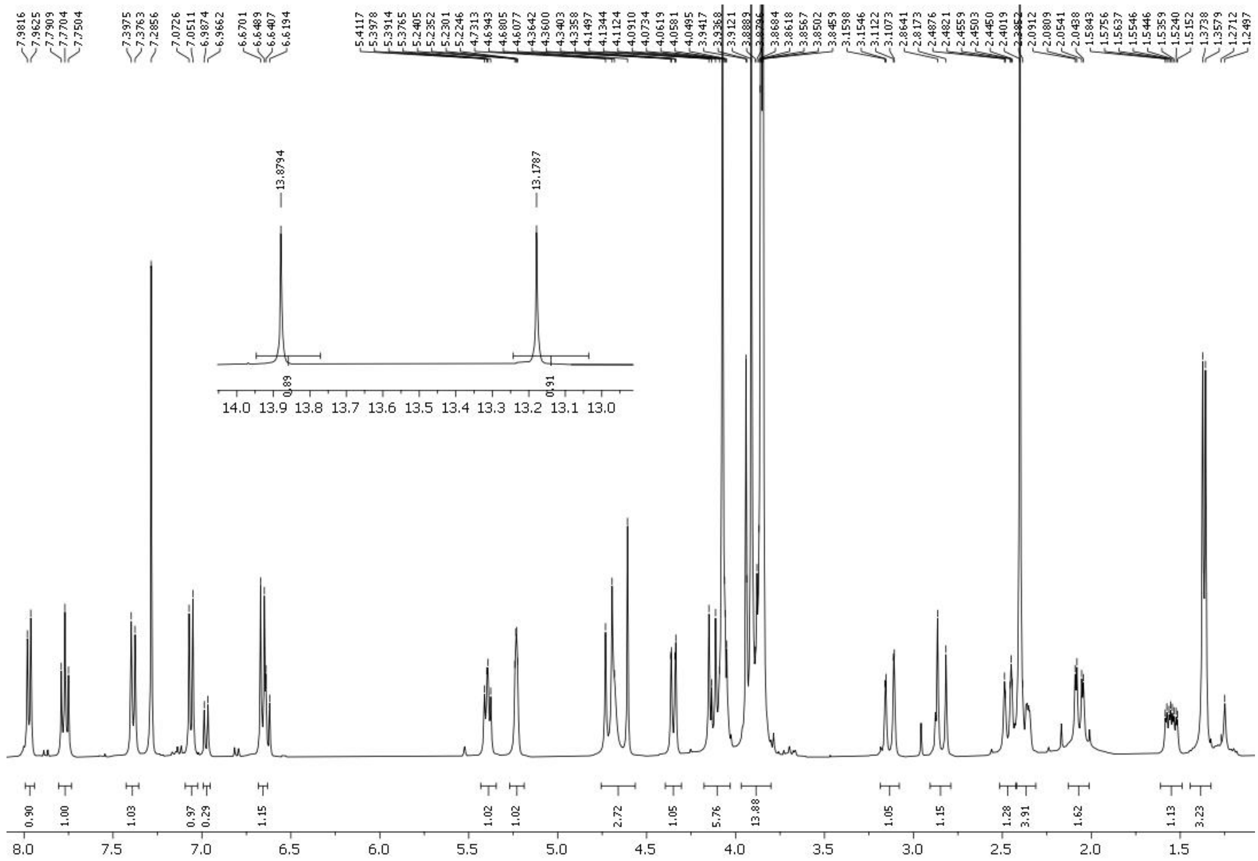

Figure S7. ^{1}H NMR spectrum of amine **4c** (400 MHz, $CDCl_3$).

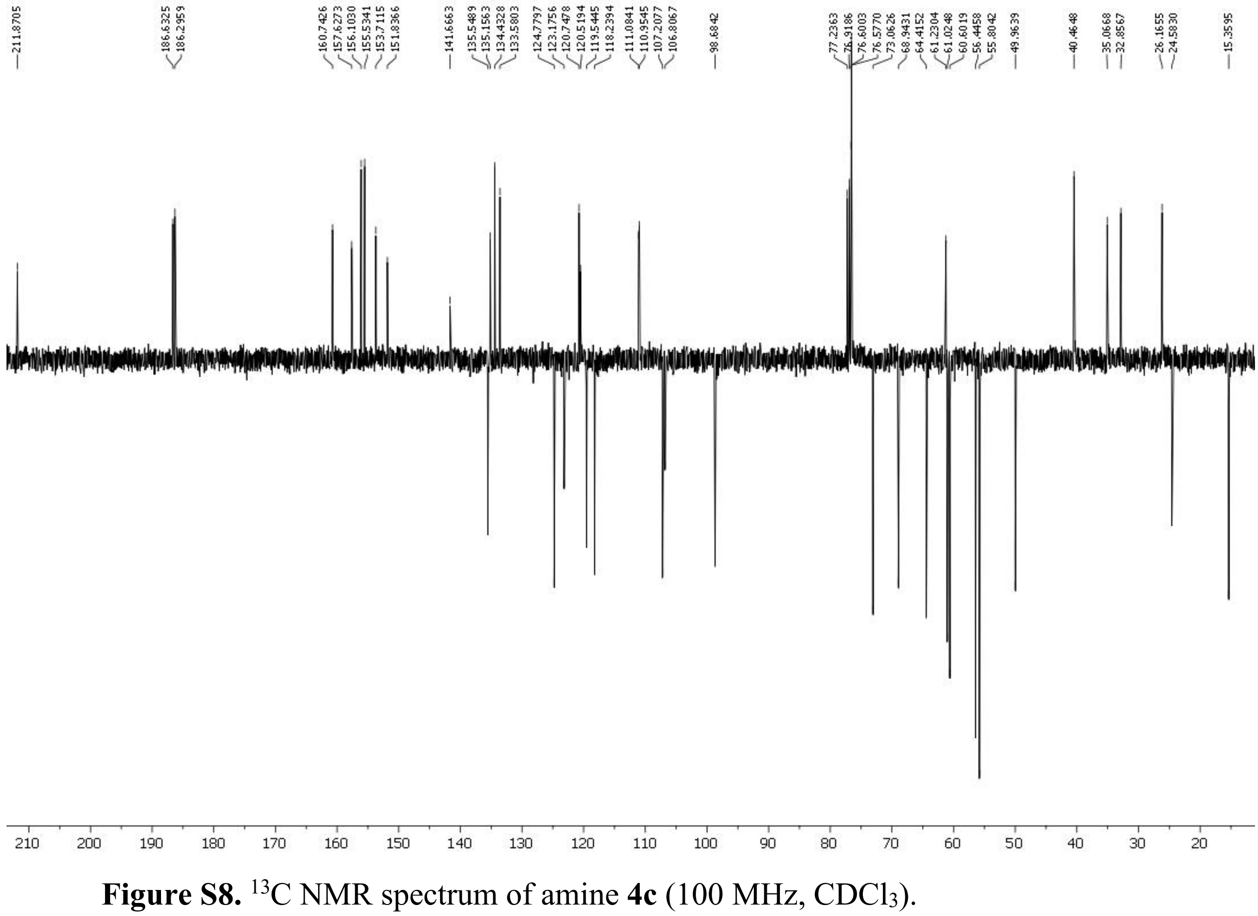

Figure S8. ^{13}C NMR spectrum of amine **4c** (100 MHz, $CDCl_3$).

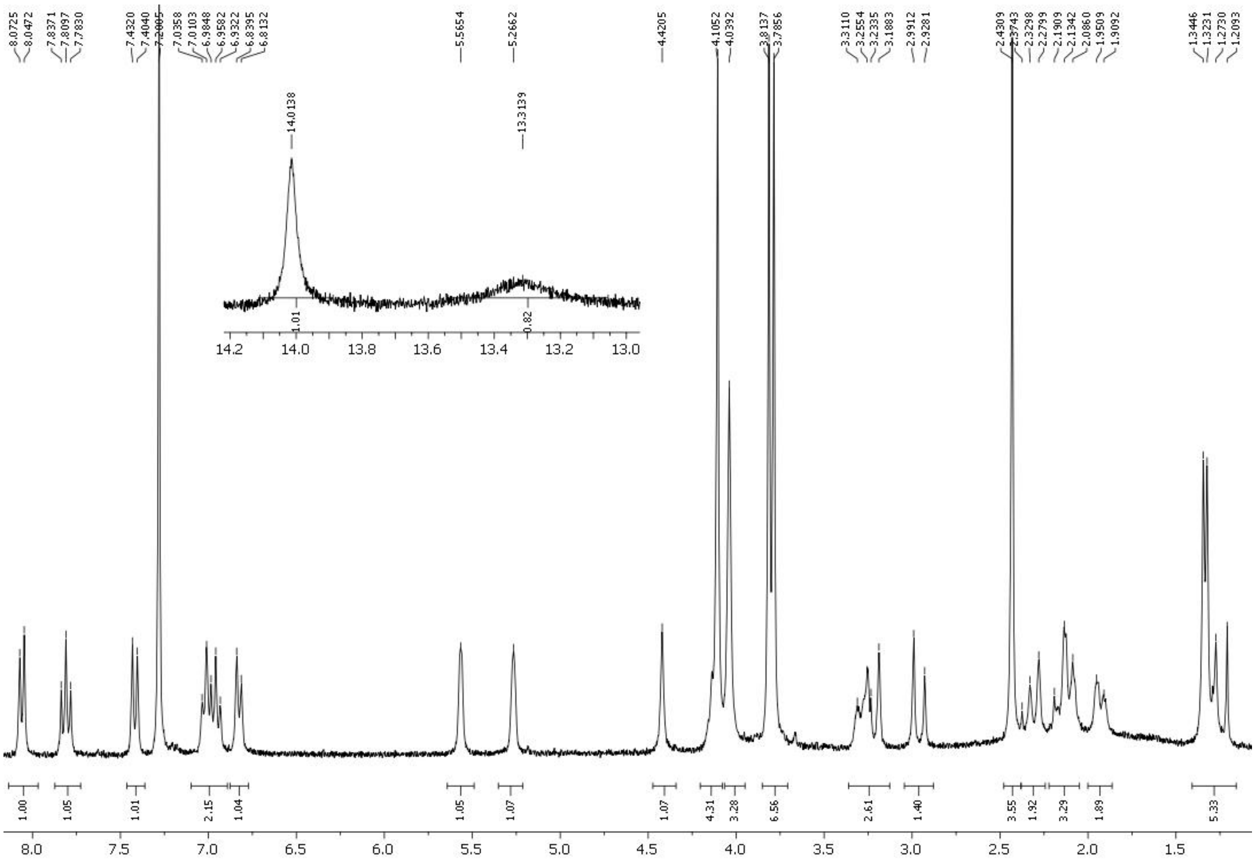

Figure S9. ^{1}H NMR spectrum of amine **4d** (300 MHz, $CDCl_3$).

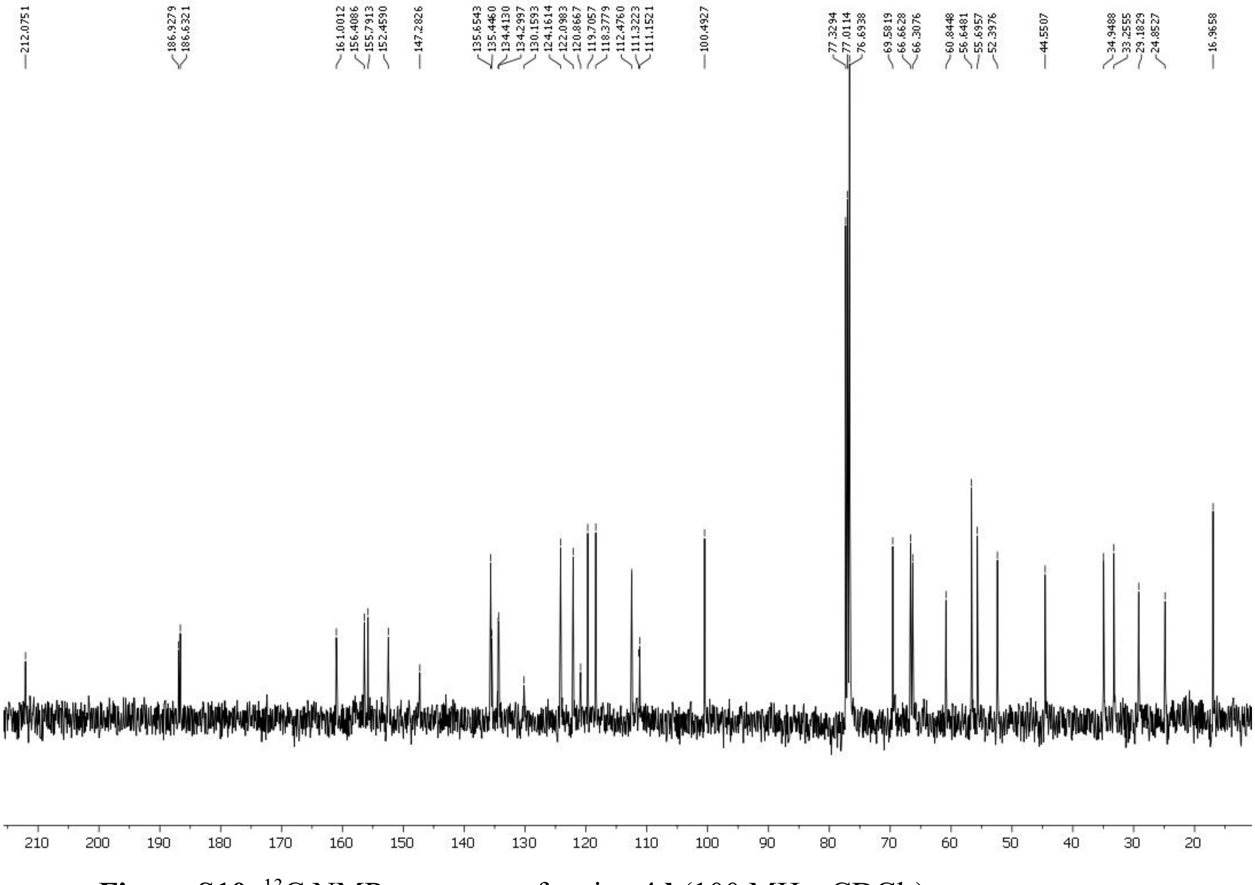

Figure S10. ^{13}C NMR spectrum of amine **4d** (100 MHz, $CDCl_3$).

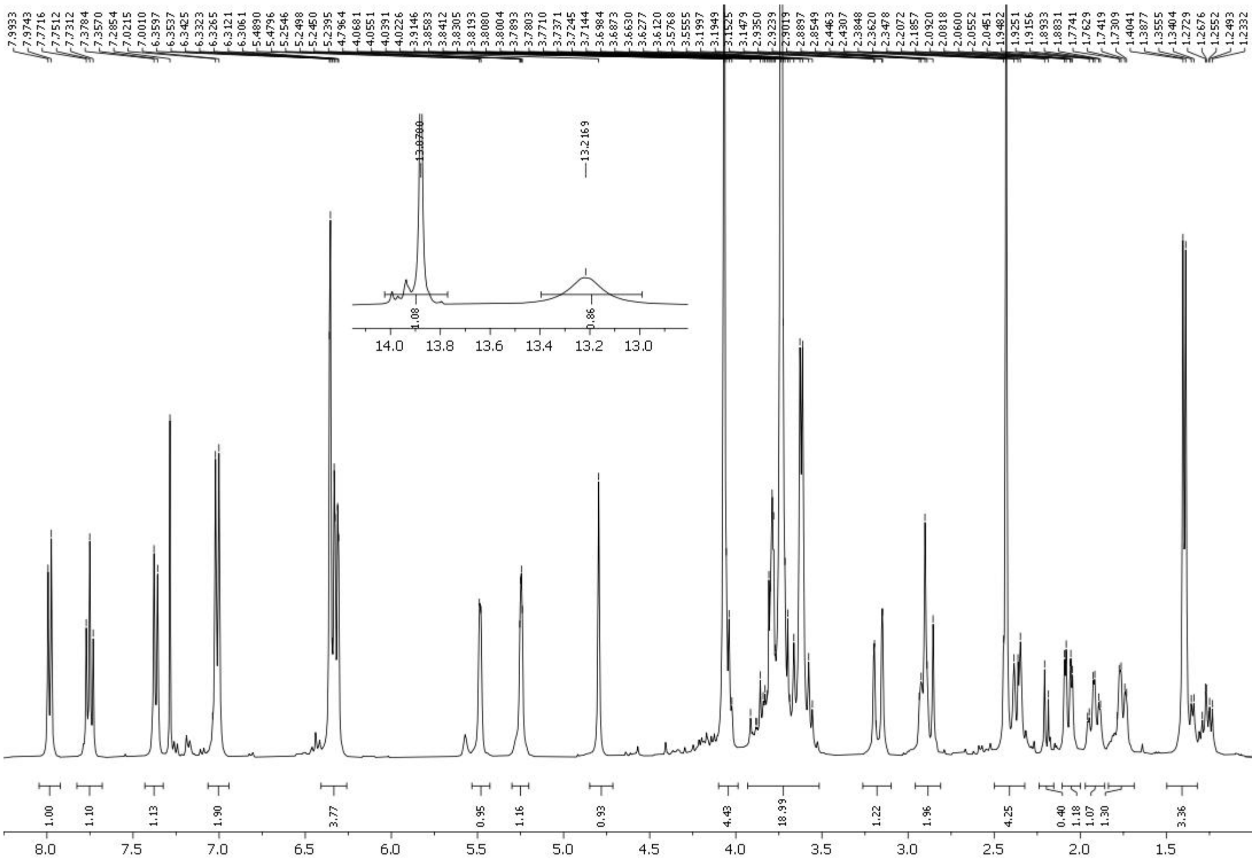

Figure S11. ^{1}H NMR spectrum of amine **4e** (400 MHz, $CDCl_3$).

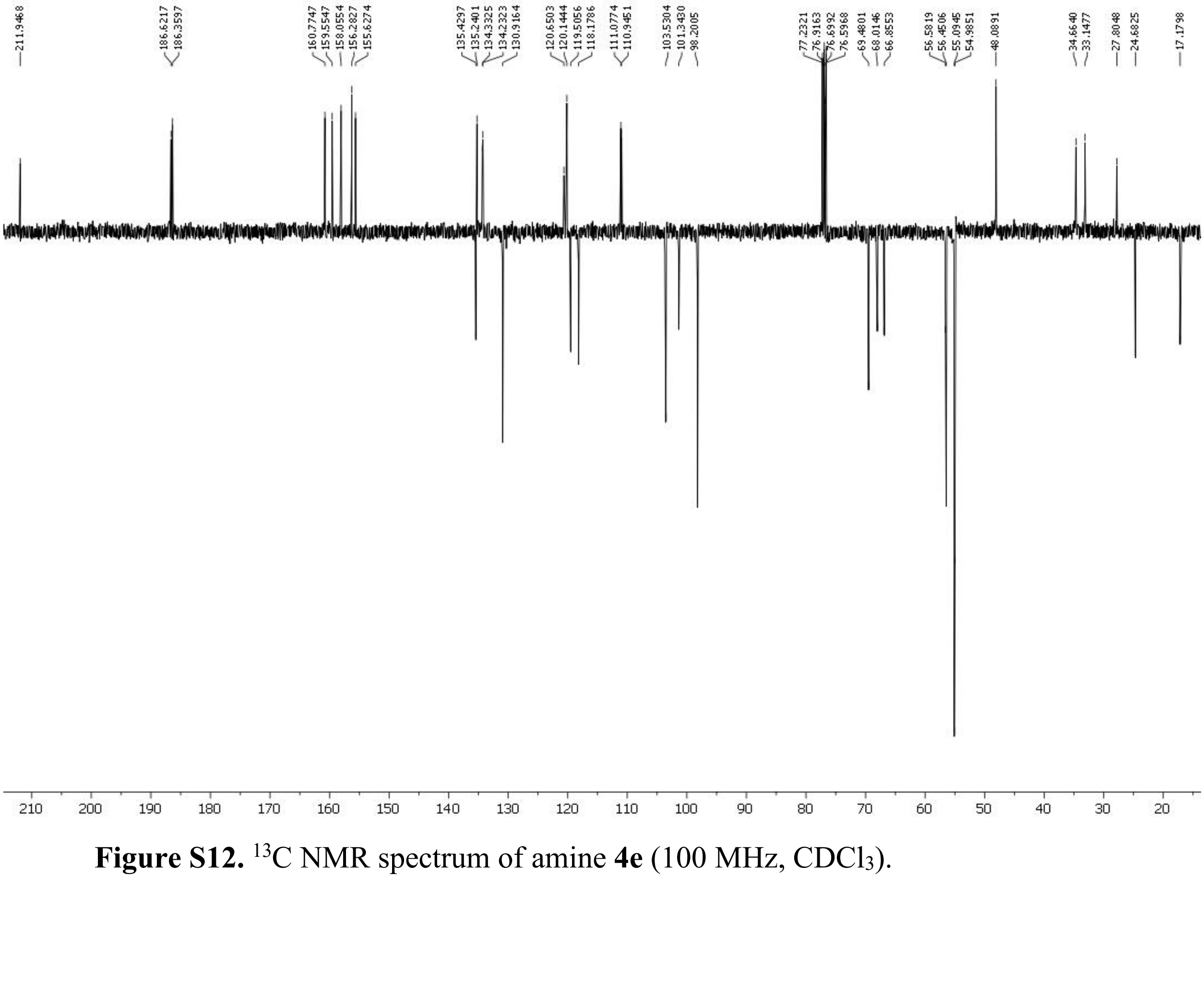

Figure S12. ^{13}C NMR spectrum of amine **4e** (100 MHz, $CDCl_3$).

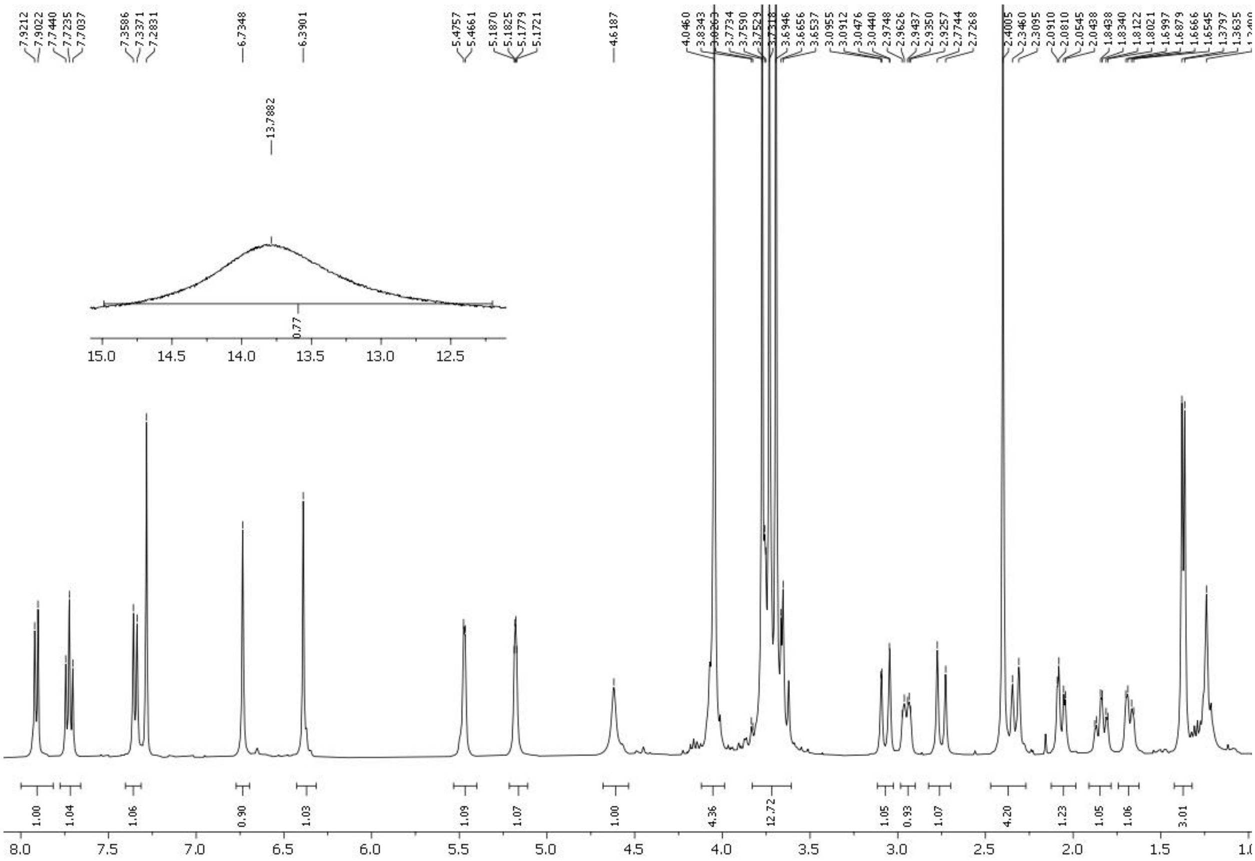

Figure S13. ^{1}H NMR spectrum of amine **4f** (400 MHz, $CDCl_3$).

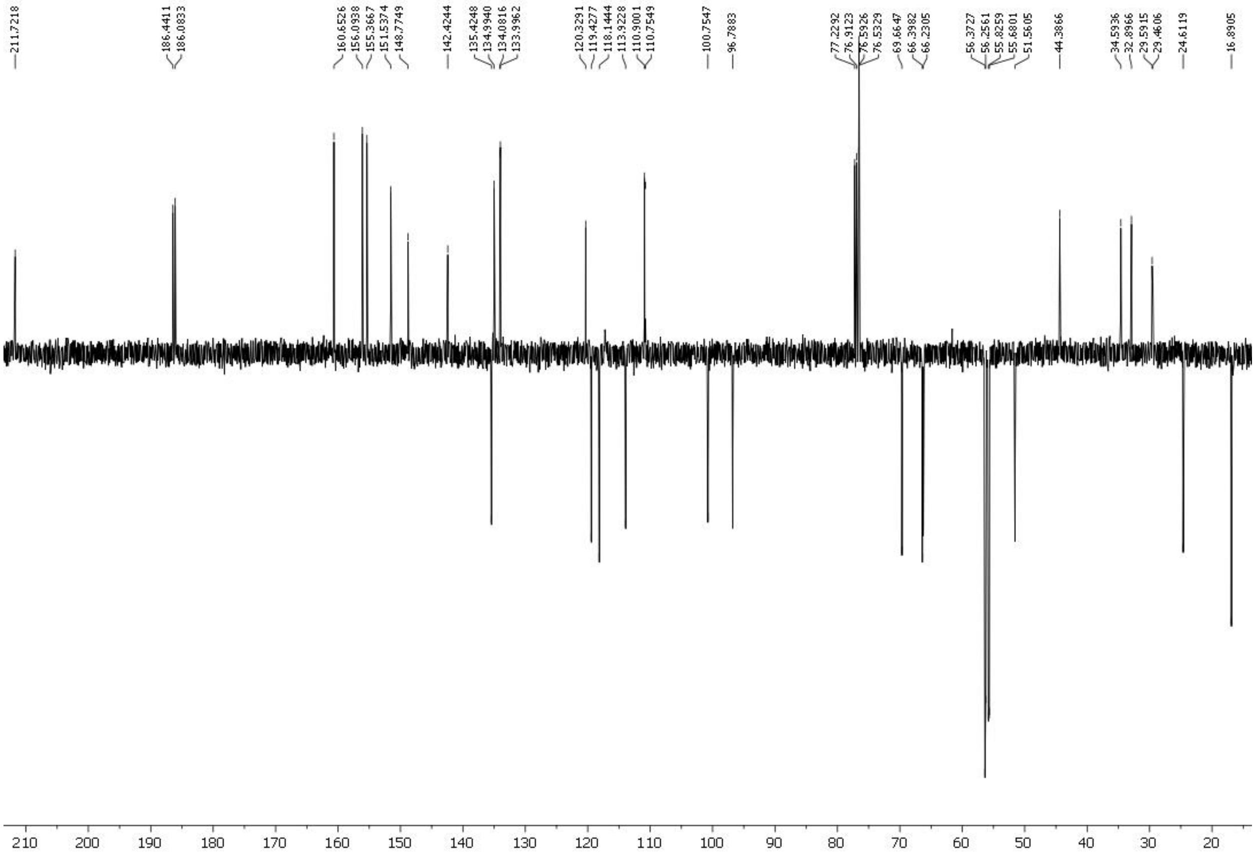

Figure S14. ^{13}C NMR spectrum of amine **4f** (100 MHz, $CDCl_3$).

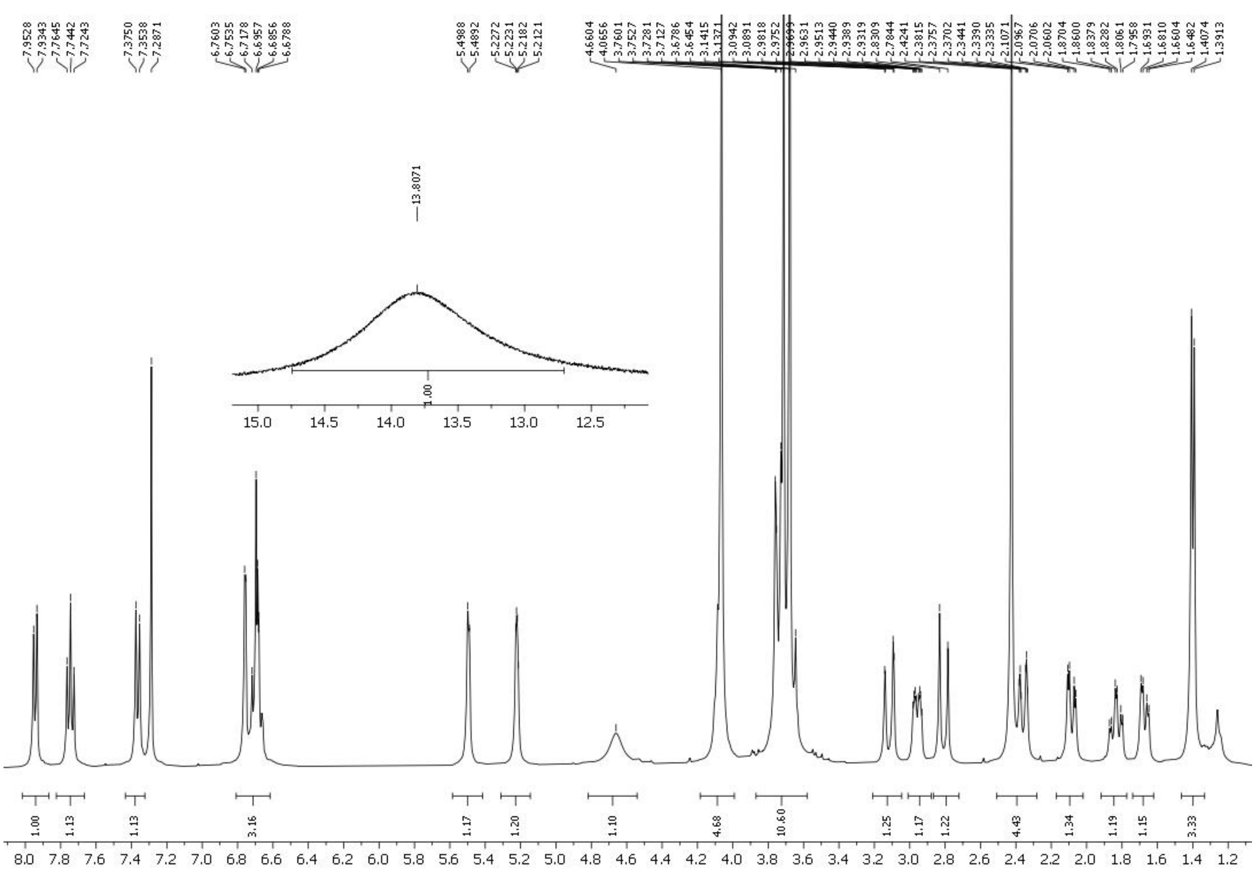

Figure S15. ^{1}H NMR spectrum of amine **4g** (400 MHz, $CDCl_3$).

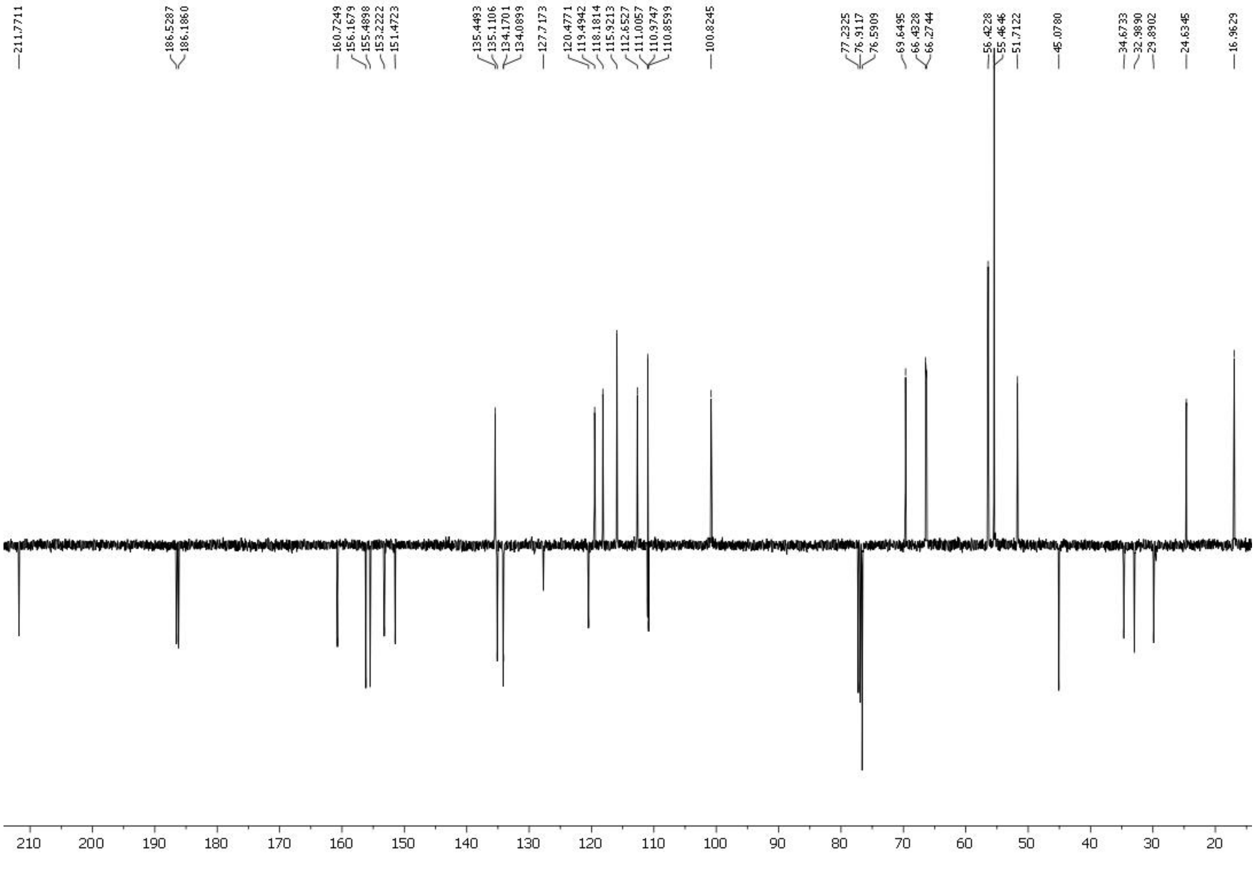

Figure S16. ^{13}C NMR spectrum of amine **4g** (100 MHz, $CDCl_3$).

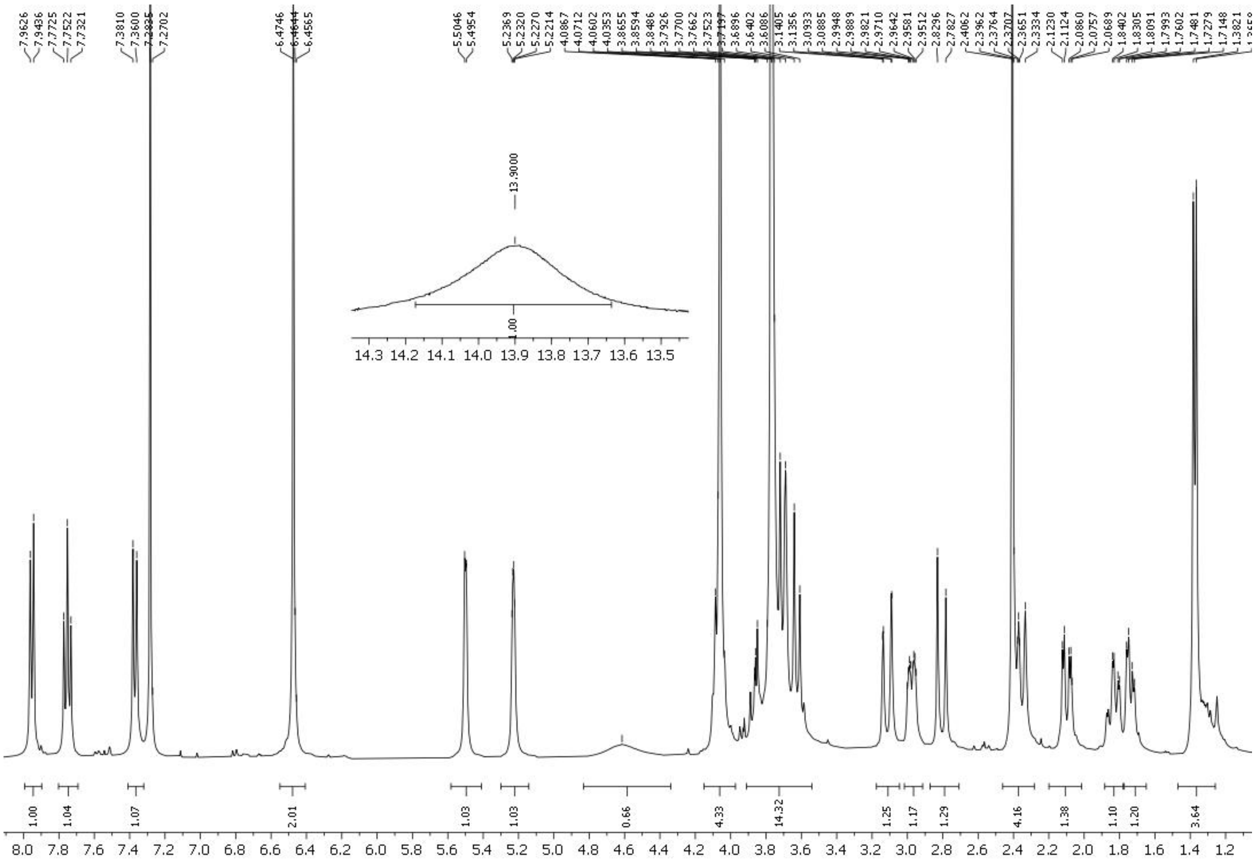

Figure S17. ^{1}H NMR spectrum of amine **4h** (400 MHz, $CDCl_3$).

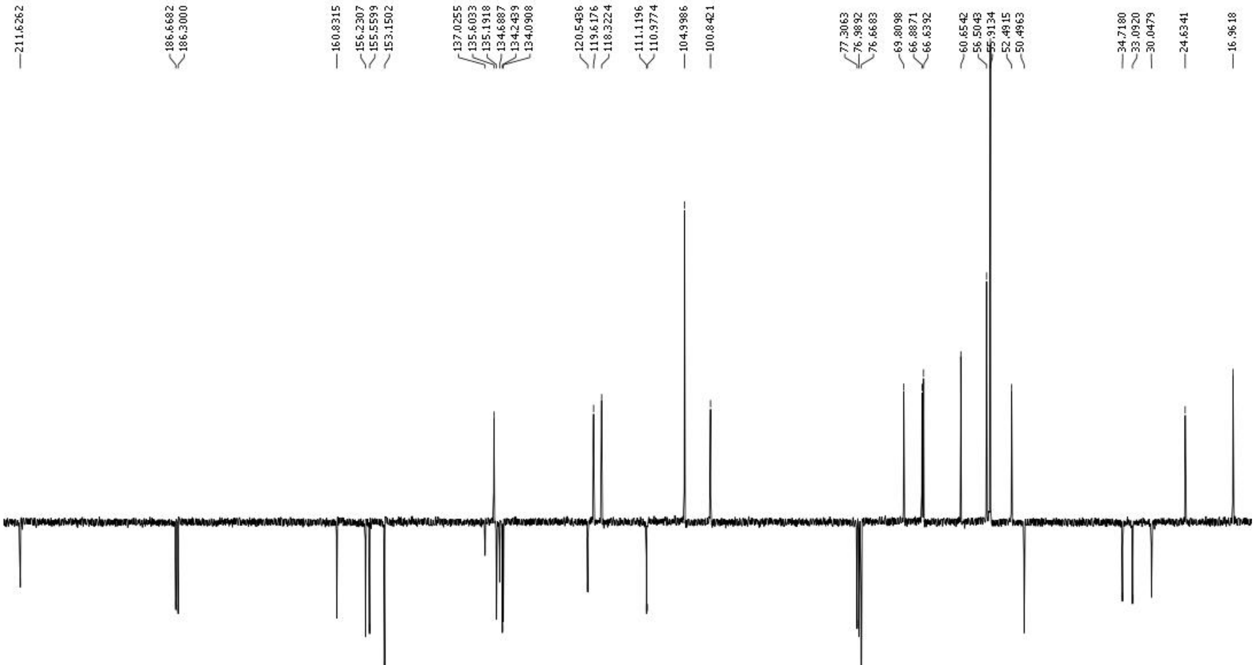

Figure S18. ^{13}C NMR spectrum of amine **4h** (100 MHz, $CDCl_3$).

MS spectra of all compounds

9/14/2023 3:22:32 PM Page 1 / 1

SHIMADZU LabSolutions **Analysis Report**

<Sample Information>

Sample Name	: esh-431		
Sample ID	: Moiseeva_AcN		
Data Filename	: esh-431_MS_no_column_phaseB-50%_DUIS_Moiseeva_AcN_001.lcd		
Method Filename	: MS_no_column_phaseB-50%_DUIS.lcm		
Batch Filename	: General_batch.lcb		
Vial #	: 1-2	Sample Type	: Unknown
Injection Volume	: 0.1 uL		
Date Acquired	: 9/14/2023 2:34:53 PM	Acquired by	: System Administrator
Date Processed	: 9/14/2023 2:37:55 PM	Processed by	: System Administrator

<Spectrum>

Line#:1 R.Time:0.508(Scan#:62)
MassPeaks:1593
RawMode:Single 0.508(62) BasePeak:678.45(3254733)
BG Mode:None Segment 1 - Event 1

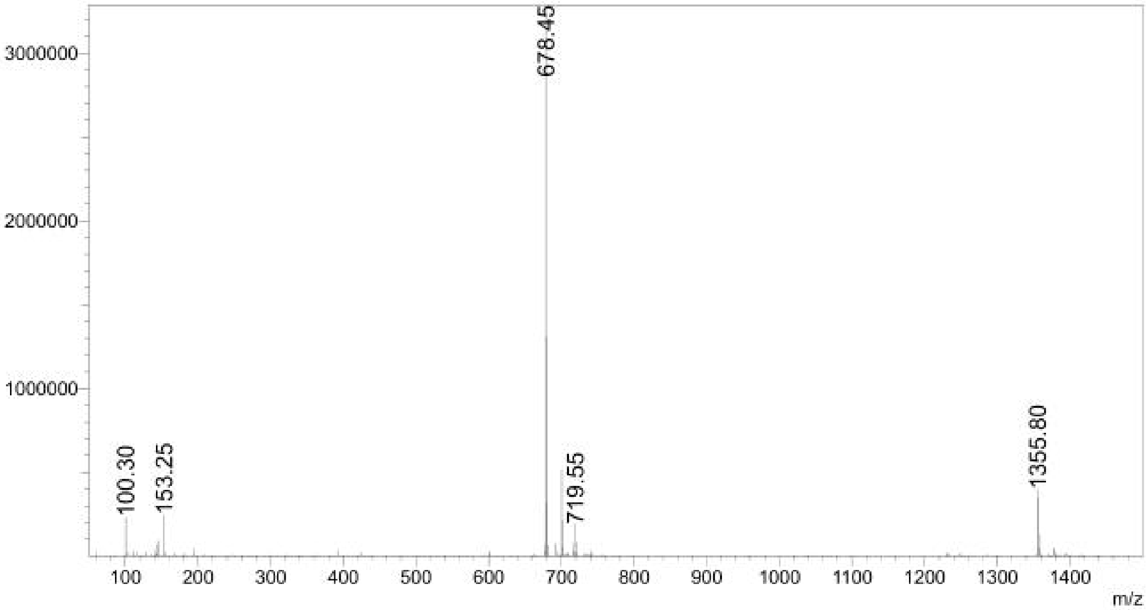

Figure S19. LC-MS spectrum of amine **4a**.

SHIMADZU LabSolutions **Analysis Report**

<Sample Information>

Sample Name	: esh-431		
Sample ID	: Moiseeva_AcN		
Data Filename	: esh-431_MS_column_phaseB-30-80_DUIS-POS-100-1500-10MIN_Moiseeva_AcN_001.l		
Method Filename	: MS_column_phaseB-30-80_DUIS-POS-100-1500-10MIN.lcm		
Batch Filename	: General_batch.lcb		
Vial #	: 1-2	Sample Type	: Unknown
Injection Volume	: 0.1 uL		
Date Acquired	: 9/14/2023 3:11:15 PM	Acquired by	: System Administrator
Date Processed	: 9/14/2023 3:21:23 PM	Processed by	: System Administrator

<Spectrum>

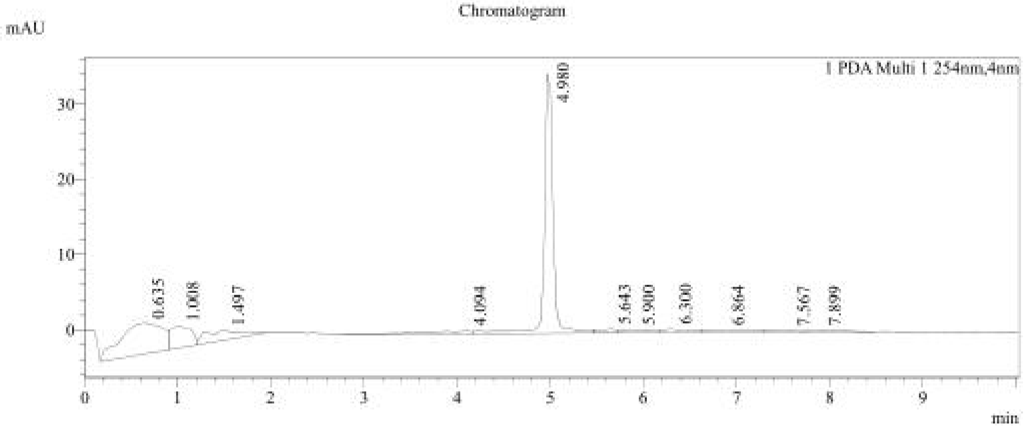

Line#:1 R.Time:5.017(Scan#:302)
MassPeaks:1549
RawMode:Single 5.017(302) BasePeak:678.50(2021758)
BG Mode:None Segment 1 - Event 1

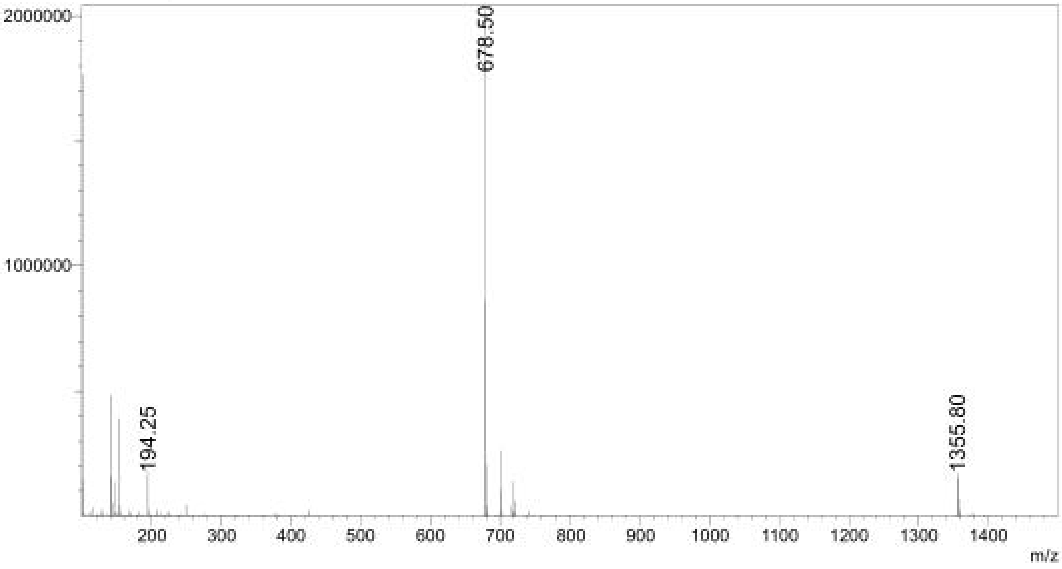

Figure S20. HPLC-MS spectrum and chromatogram of amine **4a**.

SHIMADZU LabSolutions **Analysis Report**

<Sample Information>

Sample Name	: ma-187		
Sample ID	: Moiseeva_AcN001		
Data Filename	: ma-187_MS_no_column_phaseB-80%_DUIS_Moiseeva_AcN001_001.lcd		
Method Filename	: MS_no_column_phaseB-80%_DUIS.lcm		
Batch Filename	: Khakina_batch.lcb		
Vial #	: 1-1	Sample Type	: Unknown
Injection Volume	: 0.3 uL		
Date Acquired	: 4/6/2023 2:52:34 PM	Acquired by	: System Administrator
Date Processed	: 4/6/2023 2:55:34 PM	Processed by	: System Administrator

<Spectrum>

Line#:1 R.Time:0.292(Scan#:36)
MassPeaks:1550
RawMode:Single 0.292(36) BasePeak:692.45(2321119)
BG Mode:None Segment 1 - Event 1

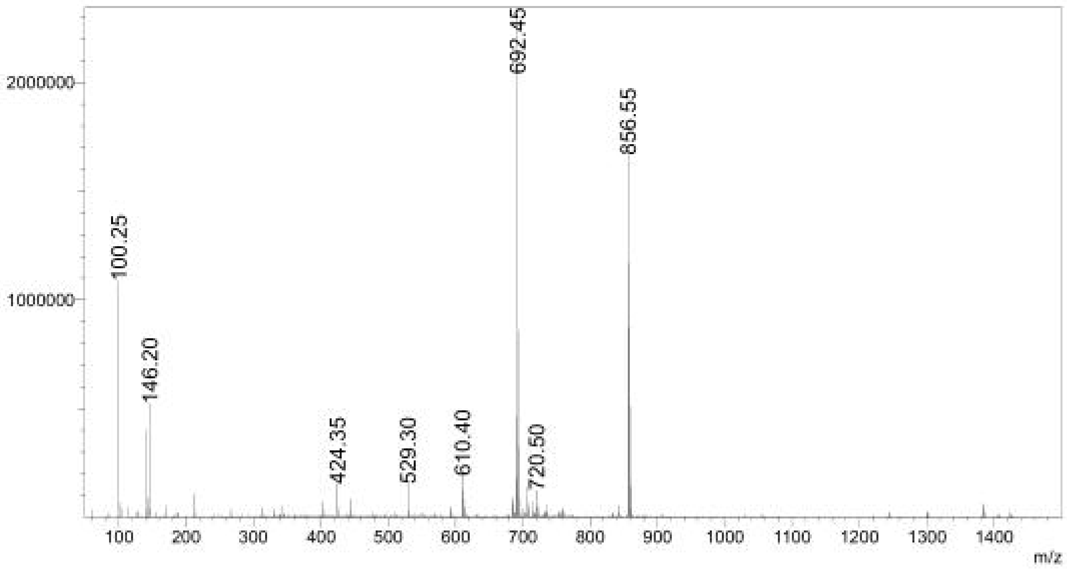

Figure S21. LC-MS spectrum of amine **4b**.

Analysis Report

SHIMADZU LabSolutions

<Sample Information>

Sample Name	: ma-187		
Sample ID	: Moiseeva_AcN001		
Data Filename	: ma-187_MS_column_phaseB_DUIS-POS-100-1500_Moiseeva_AcN001_001~1.lcd		
Method Filename	: MS_column_phaseB_DUIS-POS-100-1500.lcm		
Batch Filename	: Khakina_batch.lcb		
Vial #	: 1-1	Sample Type	: Unknown
Injection Volume	: 1 uL		
Date Acquired	: 4/6/2023 4:46:04 PM	Acquired by	: System Administrator
Date Processed	: 4/6/2023 6:00:36 PM	Processed by	: System Administrator

<Spectrum>

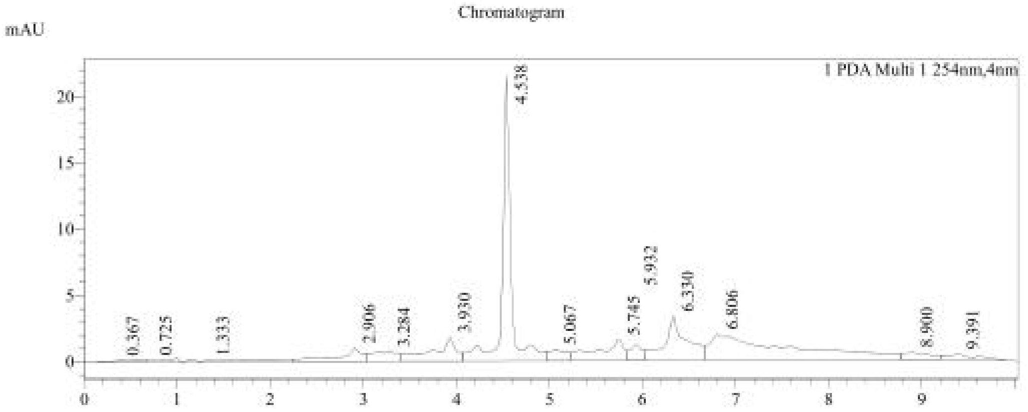

Line#:1 R.Time:4.567(Scan#:275)
MassPeaks:1548
RawMode:Single 4.567(275) BasePeak:100.15(4260389)
BG Mode:None Segment 1 - Event 1

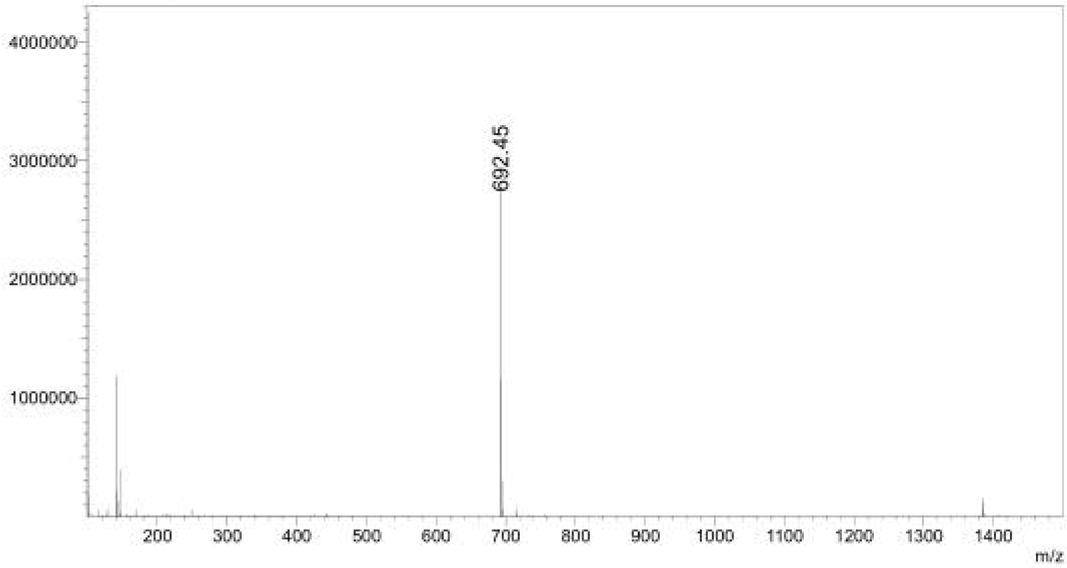

Figure S22. HPLC-MS spectrum and chromatogram of amine **4b**.

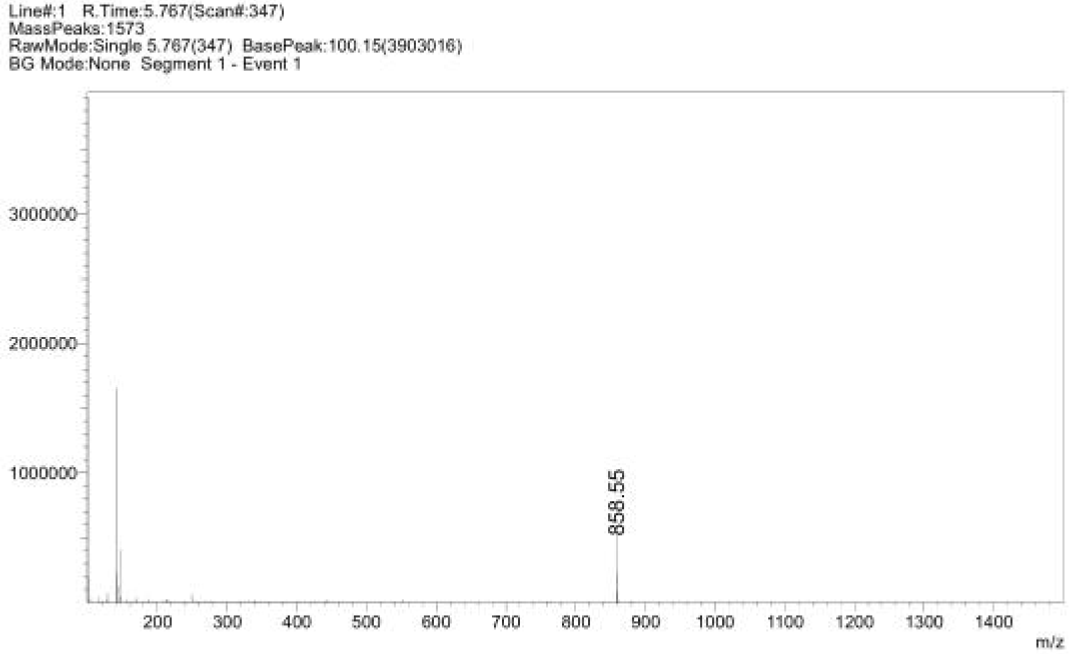

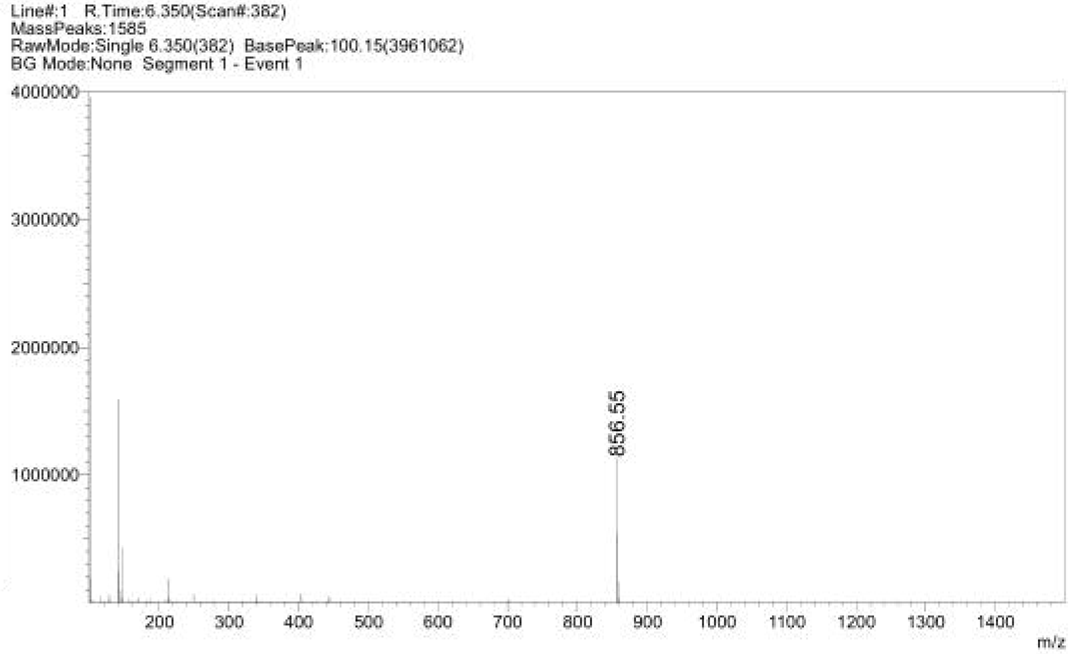

Figure S23. HPLC-MS spectra of amine **4b**.

Display Report

Analysis Info

Analysis Name	D:\Data\Kolotyrkina\2021\Moiseeva\0310016.d
Method	tune_50-1600.m
Sample Name	/BREL MA-187
Comment	C36H37NO13 mH 692.2337 calibrant added CH3CN

Acquisition Date	10.03.2021 12:28:19
Operator	BDAL@DE
Instrument / Ser#	micrOTOF 10248

Acquisition Parameter

Source Type	ESI	Ion Polarity	Positive	Set Nebulizer	1.0 Bar
Focus	Not active			Set Dry Heater	200 °C
Scan Begin	50 m/z	Set Capillary	4500 V	Set Dry Gas	4.0 l/min
Scan End	1600 m/z	Set End Plate Offset	-500 V	Set Divert Valve	Waste

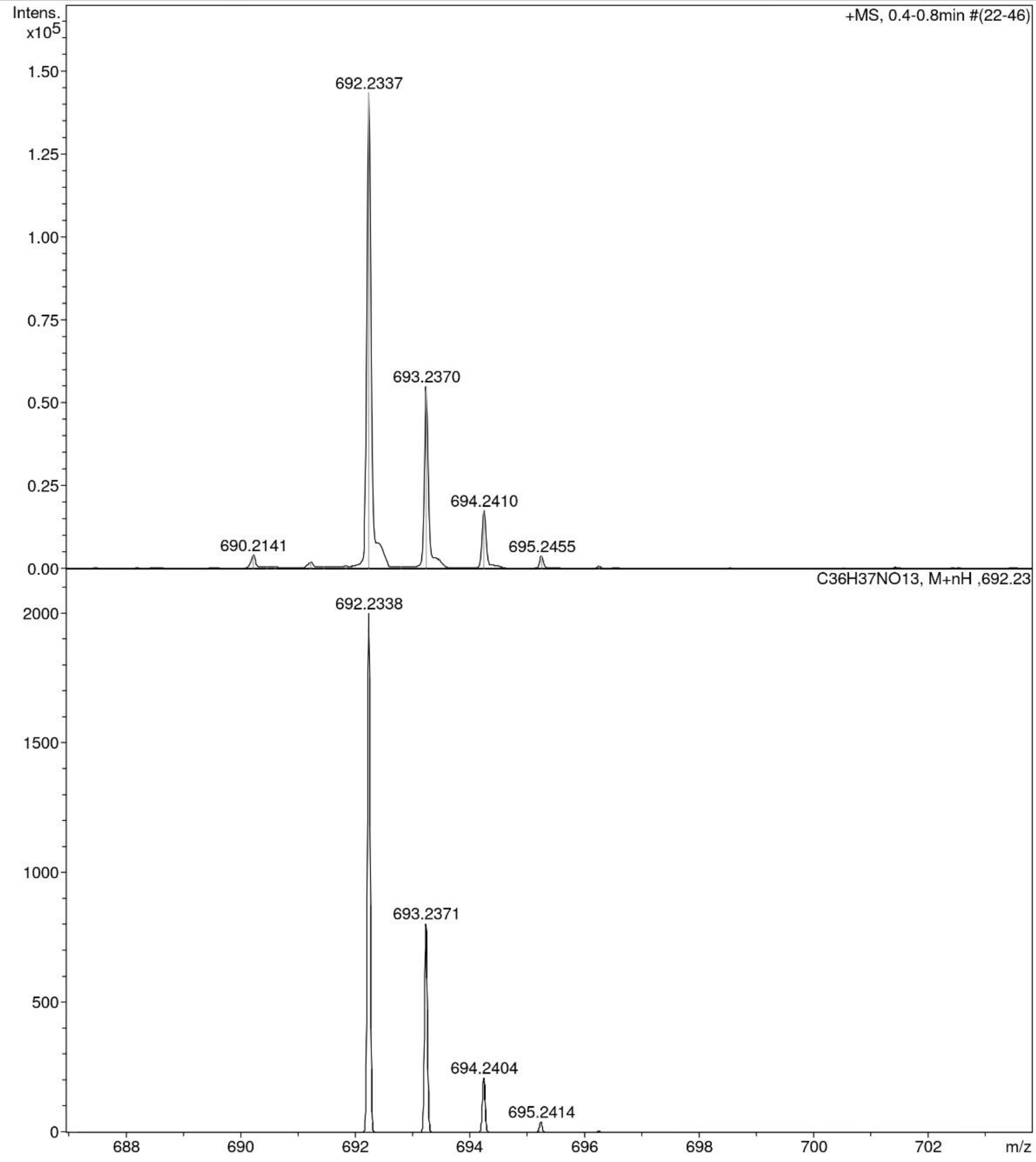

Bruker Compass DataAnalysis 4.0 printed: 10.03.2021 12:31:29 Page 1 of 1

Figure S24. HRMS spectrum of amine **4b**.

SHIMADZU LabSolutions

Analysis Report

<Sample Information>

Sample Name	: ma-263		
Sample ID	: Moiseeva_AcN002		
Data Filename	: ma-263_MS_no_column_phaseB-80%_DUIS_Moiseeva_AcN002_002.lcd		
Method Filename	: MS_no_column_phaseB-80%_DUIS.lcm		
Batch Filename	: Khakina_batch.lcb		
Vial #	: 1-2	Sample Type	: Unknown
Injection Volume	: 2 uL		
Date Acquired	: 4/6/2023 2:56:07 PM	Acquired by	: System Administrator
Date Processed	: 4/6/2023 2:59:08 PM	Processed by	: System Administrator

<Spectrum>

Line#:1 R.Time:0.308(Scan#:38)
MassPeaks:1536
RawMode:Single 0.308(38) BasePeak:886.60(1309975)
BG Mode:None Segment 1 - Event 1

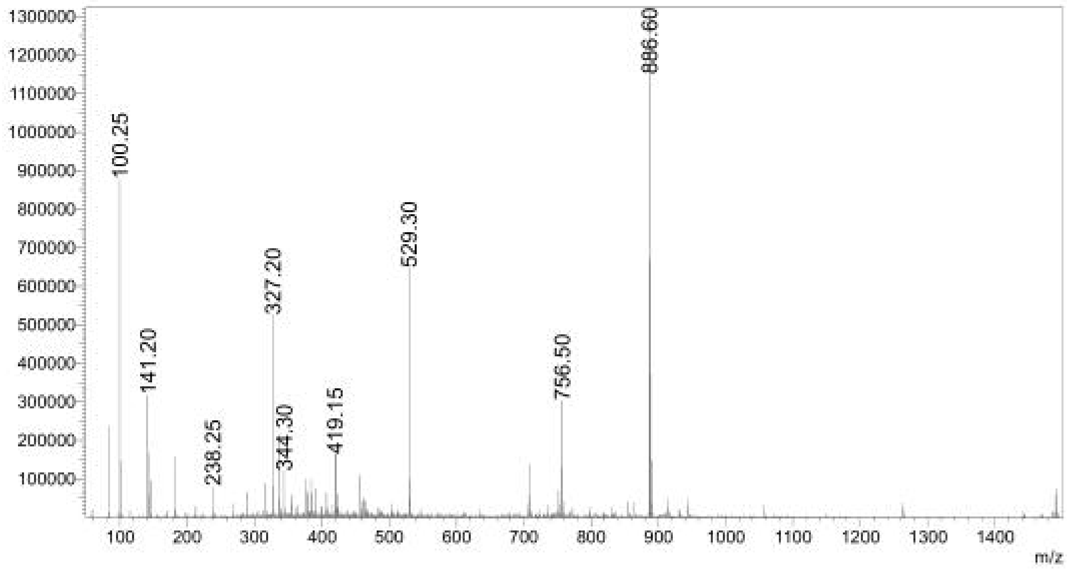

Figure S25. LC-MS spectrum of amine **4c**.

Analysis Report

<Sample Information>

Sample Name	: ma-263		
Sample ID	: Moiseeva_AcN002		
Data Filename	: ma-263_MS_column_phaseB_DUIS-POS-100-1500_Moiseeva_AcN002_002.lcd		
Method Filename	: MS_column_phaseB_DUIS-POS-100-1500.lcm		
Batch Filename	: Khakina_batch.lcb		
Vial #	: 1-2	Sample Type	: Unknown
Injection Volume	: 4 uL		
Date Acquired	: 4/6/2023 4:56:44 PM	Acquired by	: System Administrator
Date Processed	: 4/6/2023 5:06:51 PM	Processed by	: System Administrator

<Spectrum>

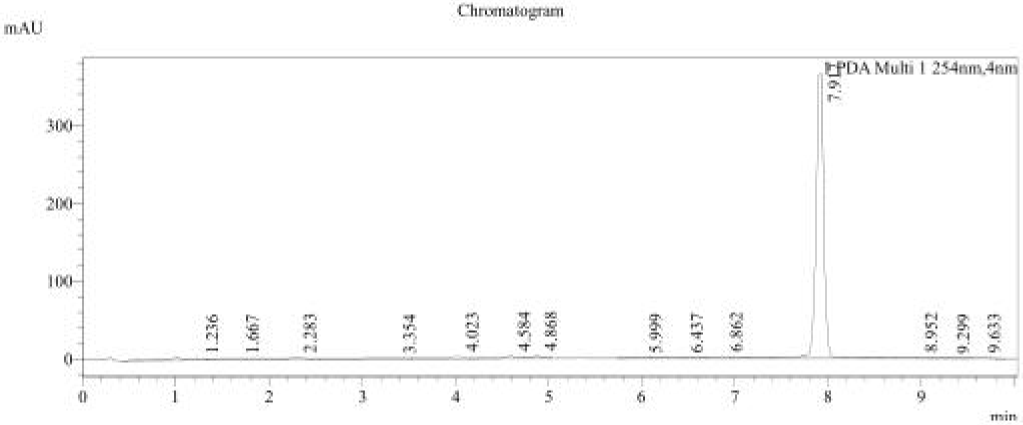

Line#:1 R.Time:4.583(Scan#:276)
MassPeaks:1598
RawMode:Single 4.583(276) BasePeak:100.15(4343648)
BG Mode:None Segment 1 - Event 1

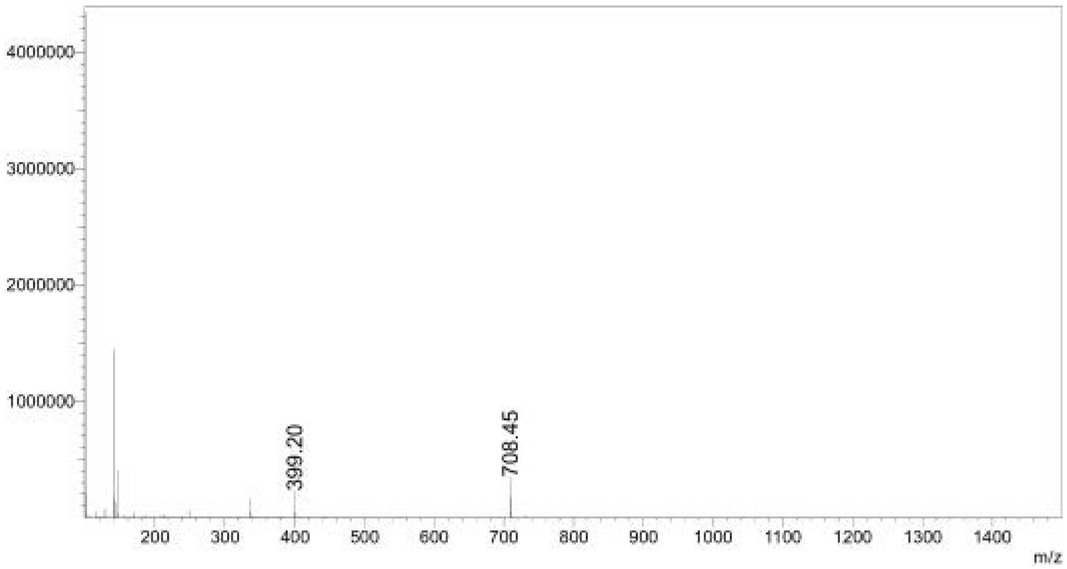

Figure S26. HPLC-MS spectrum and chromatogram of amine **4c**.

Display Report

Analysis Info

Analysis Name	D:\Data\Kolotyrkina\2021\Moiseeva\0310007.d	Acquisition Date	10.03.2021 10:59:01
Method	tune_50-1600.m	Operator	BDAL@DE
Sample Name	/BREL MA-263	Instrument / Ser#	micrOTOF 10248
Comment	C37H41NO13 mH 708.2650 calibrant added CH3CN		

Acquisition Parameter

Source Type	ESI	Ion Polarity	Positive	Set Nebulizer	1.0 Bar
Focus	Not active			Set Dry Heater	200 °C
Scan Begin	50 m/z	Set Capillary	4500 V	Set Dry Gas	4.0 l/min
Scan End	1600 m/z	Set End Plate Offset	-500 V	Set Divert Valve	Waste

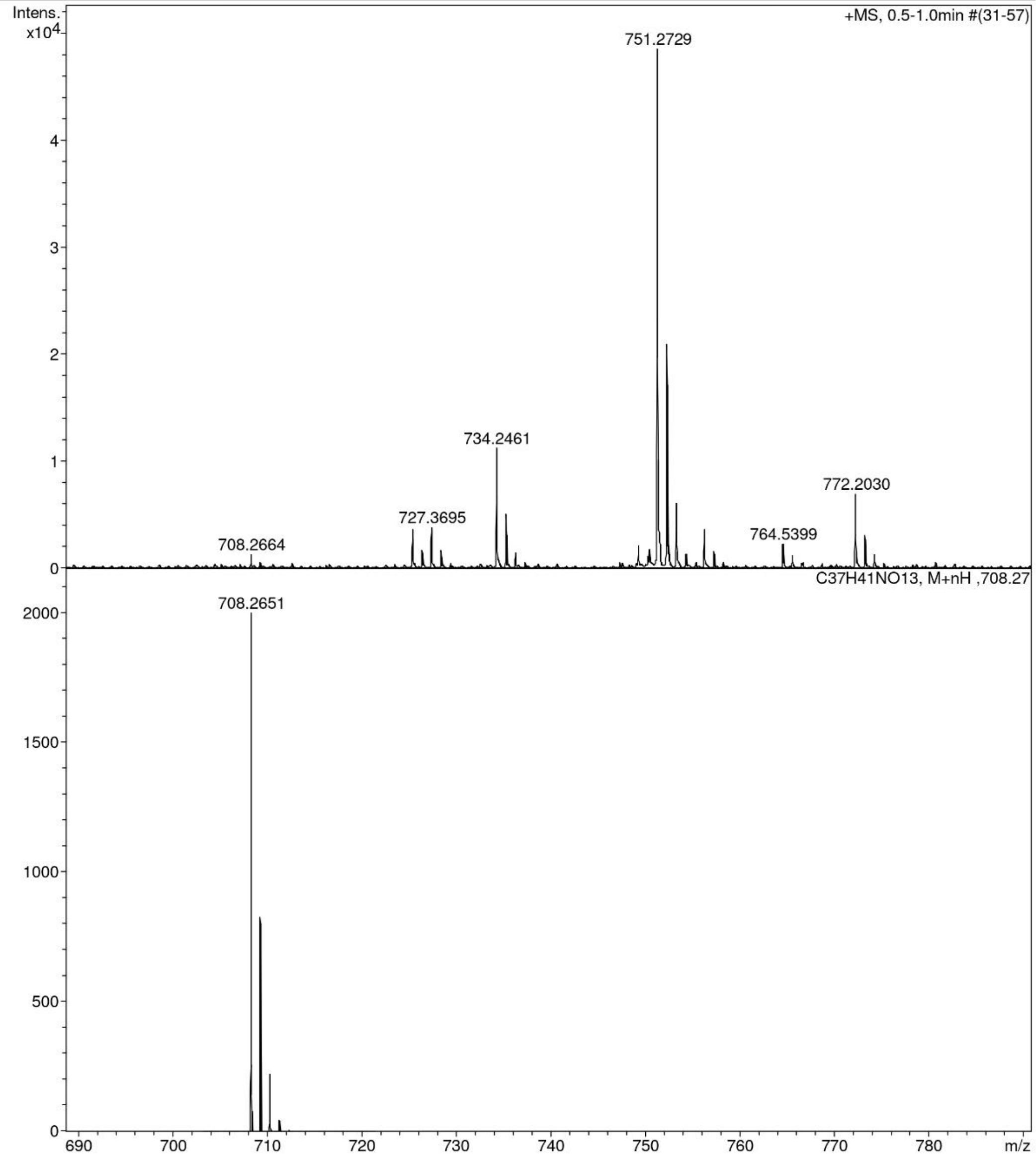

Bruker Compass DataAnalysis 4.0 printed: 10.03.2021 11:06:03 Page 1 of 1

Figure S27. HRMS spectrum of amine **4c**.

SHIMADZU LabSolutions

Analysis Report

<Sample Information>

Sample Name	: ma-265		
Sample ID	: Moiseeva_AcN003		
Data Filename	: ma-265_MS_no_column_phaseB-80%_DUIS_Moiseeva_AcN003_003.lcd		
Method Filename	: MS_no_column_phaseB-80%_DUIS.lcm		
Batch Filename	: Khakina_batch.lcb		
Vial #	: 1-3	Sample Type	: Unknown
Injection Volume	: 0.5 uL		
Date Acquired	: 4/6/2023 2:59:42 PM	Acquired by	: System Administrator
Date Processed	: 4/6/2023 3:02:43 PM	Processed by	: System Administrator

<Spectrum>

Line#:1 R.Time:0.292(Scan#:36)
MassPeaks:1573
RawMode:Single 0.292(36) BasePeak:678.50(5565696)
BG Mode:None Segment 1 - Event 1

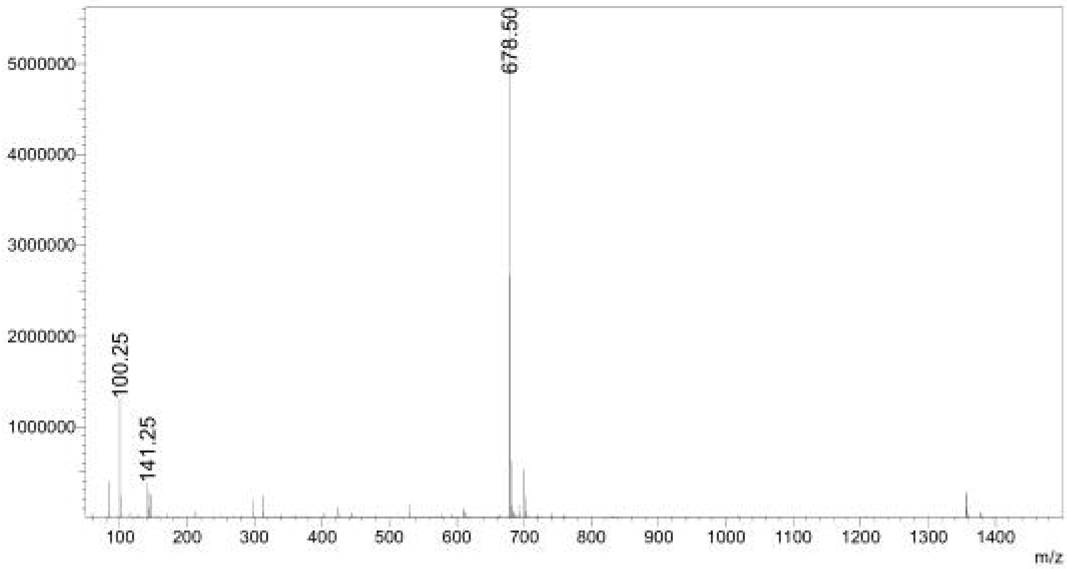

Figure S28. LC-MS spectrum of amine **4d**.

SHIMADZU LabSolutions

Analysis Report

<Sample Information>

Sample Name	: ma-265		
Sample ID	: Moiseeva_AcN003		
Data Filename	: ma-265_MS_column_phaseB_DUIS-POS-100-1500_Moiseeva_AcN003_003.lcd		
Method Filename	: MS_column_phaseB_DUIS-POS-100-1500.lcm		
Batch Filename	: Khakina_batch.lcb		
Vial #	: 1-3	Sample Type	: Unknown
Injection Volume	: 1 uL		
Date Acquired	: 4/6/2023 5:07:22 PM	Acquired by	: System Administrator
Date Processed	: 4/6/2023 6:17:21 PM	Processed by	: System Administrator

<Spectrum>

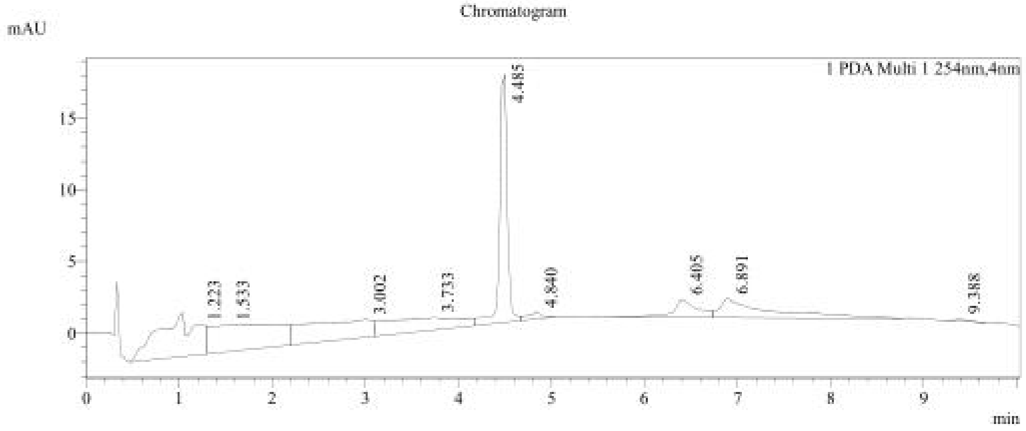

Line#:1 R.Time:4.517(Scan#:272)
MassPeaks:1560
RawMode:Single 4.517(272) BasePeak:678.45(3638510)
BG Mode:None Segment 1 - Event 1

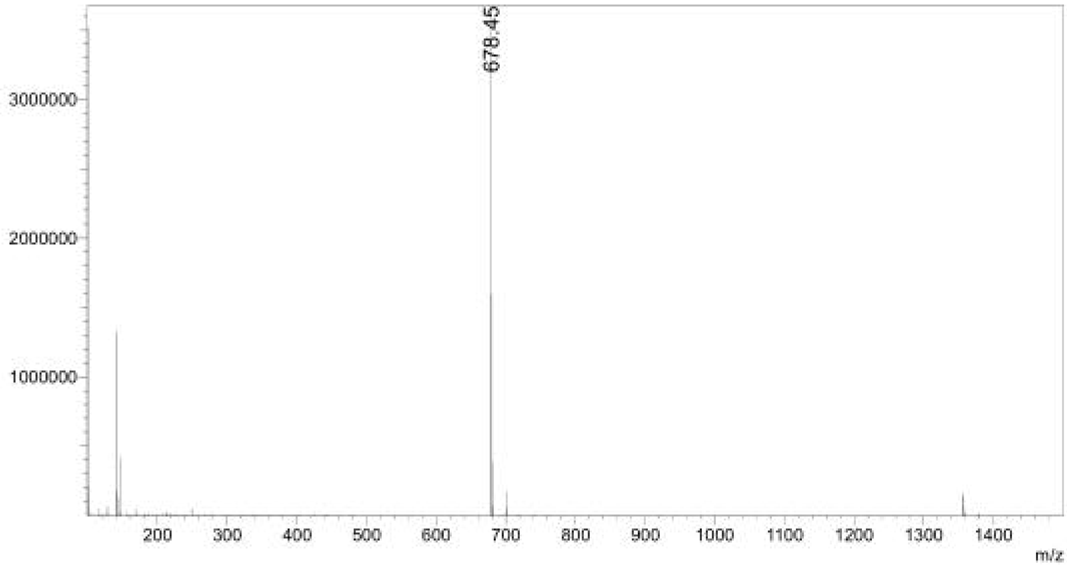

Figure S29. HPLC-MS spectrum and chromatogram of amine **4d**.

Display Report

Analysis Info

Analysis Name	D:\Data\Kolotyrkina\2021\Moiseeva\0310021.d	Acquisition Date	10.03.2021 13:00:56
Method	tune_50-1600.m	Operator	BDAL@DE
Sample Name	/BREL MA-265	Instrument / Ser#	micrOTOF 10248
Comment	C36H39NO12 mH 678.2545 calibrant added CH3CN		

Acquisition Parameter

Source Type	ESI	Ion Polarity	Positive	Set Nebulizer	1.0 Bar
Focus	Not active			Set Dry Heater	200 °C
Scan Begin	50 m/z	Set Capillary	4500 V	Set Dry Gas	4.0 l/min
Scan End	1600 m/z	Set End Plate Offset	-500 V	Set Divert Valve	Waste

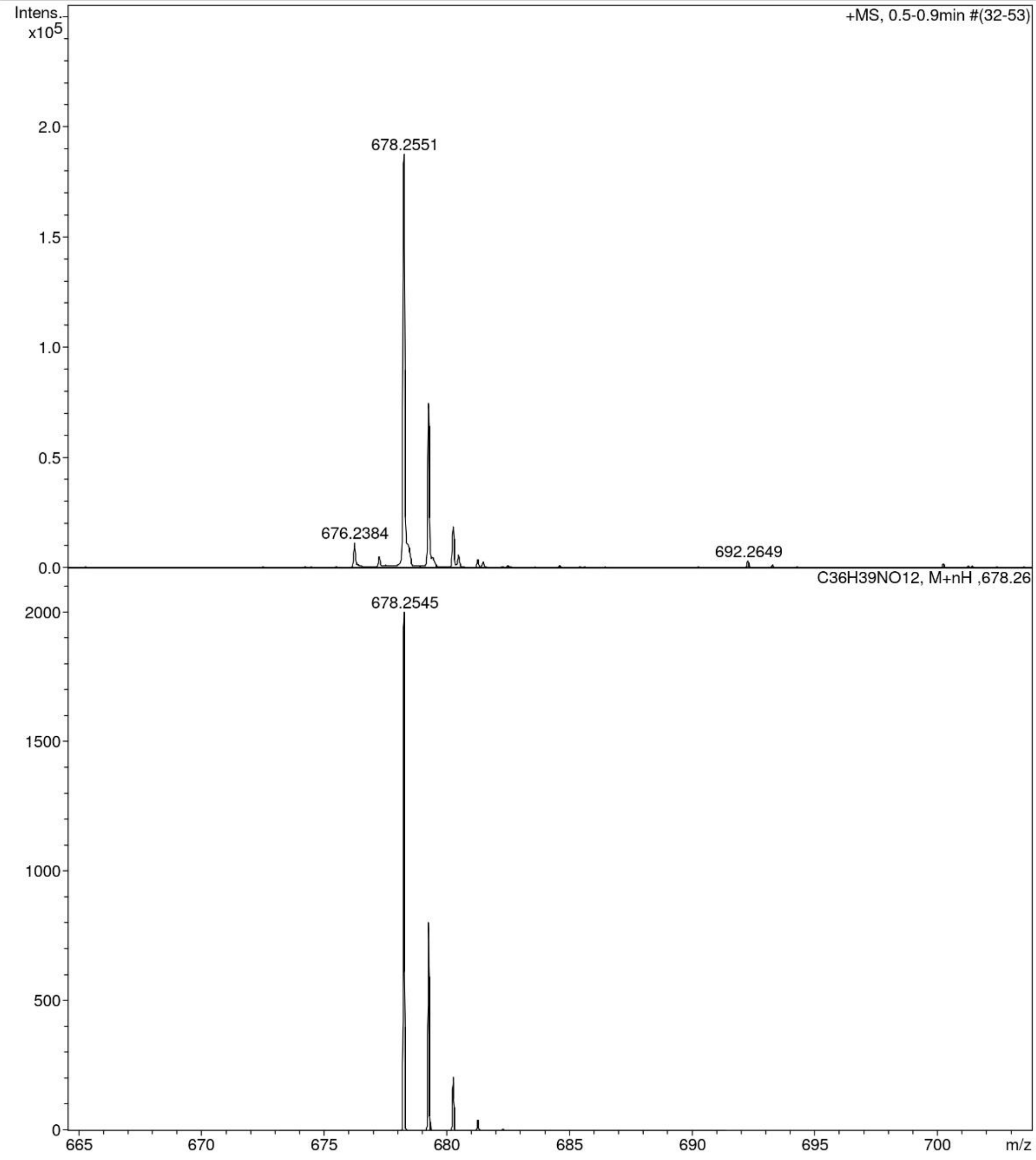

Bruker Compass DataAnalysis 4.0 printed: 10.03.2021 13:03:16 Page 1 of 1

Figure S30. HRMS spectrum of amine **4d**.

SHIMADZU LabSolutions **Analysis Report**

<Sample Information>

Sample Name : ma-266
Sample ID : Moiseeva_AcN004
Data Filename : ma-266_MS_no_column_phaseB-80%_DUIS_Moiseeva_AcN004_004.lcd
Method Filename : MS_no_column_phaseB-80%_DUIS.lcm
Batch Filename : Khakina_batch.lcb
Vial # : 1-4 Sample Type : Unknown
Injection Volume : 0.1 uL
Date Acquired : 4/6/2023 3:03:15 PM Acquired by : System Administrator
Date Processed : 4/6/2023 3:06:18 PM Processed by : System Administrator

<Spectrum>

Line#:1 R.Time:0.292(Scan#:36)
MassPeaks:1574
RawMode:Single 0.292(36) BasePeak:828.60(4896793)
BG Mode:None Segment 1 - Event 1

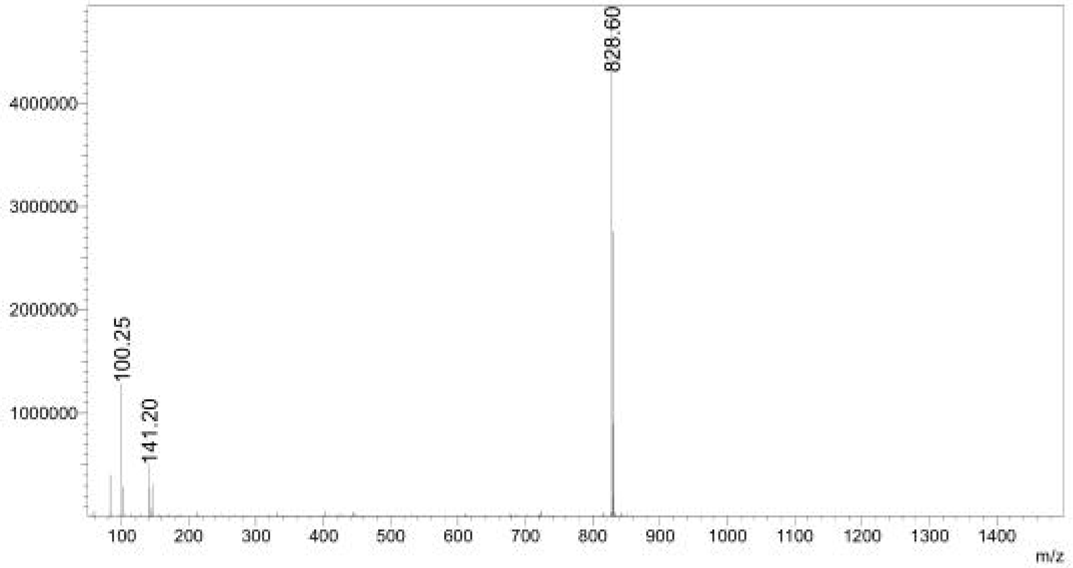

Figure S31a. LC-MS spectrum of amine **4e**. Here and below the formation of an atypical ion in the mass spectrum was observed. Instead of the usual one $[M + H]^+$ under these conditions, the ion shown in the Figure 35b was formed. A similar picture was observed in the MS spectra of **4b**, **4c**, **4f**.

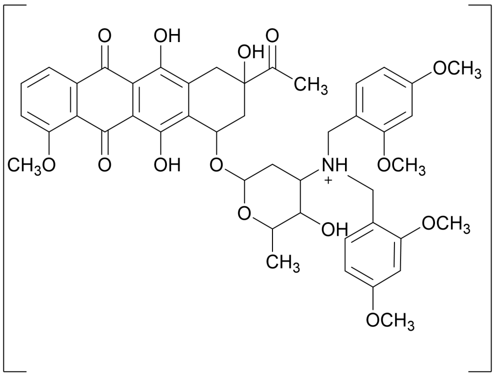

Figure S31b. The previously shown ion $[M + R + H]^+$.

SHIMADZU LabSolutions

Analysis Report

<Sample Information>

Sample Name : ma-266
Sample ID : Moiseeva_AcN004
Data Filename : ma-266_MS_column_phaseB_DUIS-POS-100-1500_Moiseeva_AcN004_003.lcd
Method Filename : MS_column_phaseB_DUIS-POS-100-1500.lcm
Batch Filename : Khakina_batch.lcb
Vial # : 1-4 Sample Type : Unknown
Injection Volume : 0.1 uL
Date Acquired : 4/6/2023 4:12:59 PM Acquired by : System Administrator
Date Processed : 4/6/2023 4:23:06 PM Processed by : System Administrator

<Spectrum>

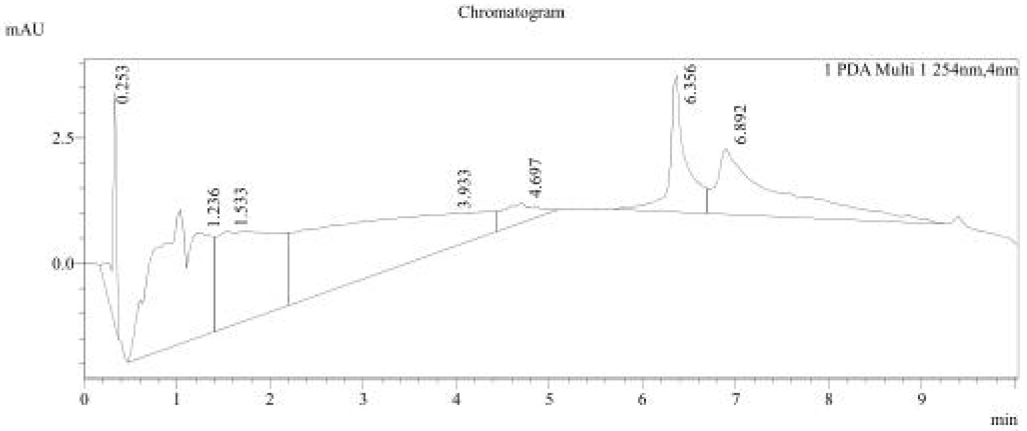

Line#:1 R.Time:6.350(Scan#:382)
MassPeaks:1597
RawMode:Single 6.350(382) BasePeak:100.15(4001076)
BG Mode:None Segment 1 - Event 1

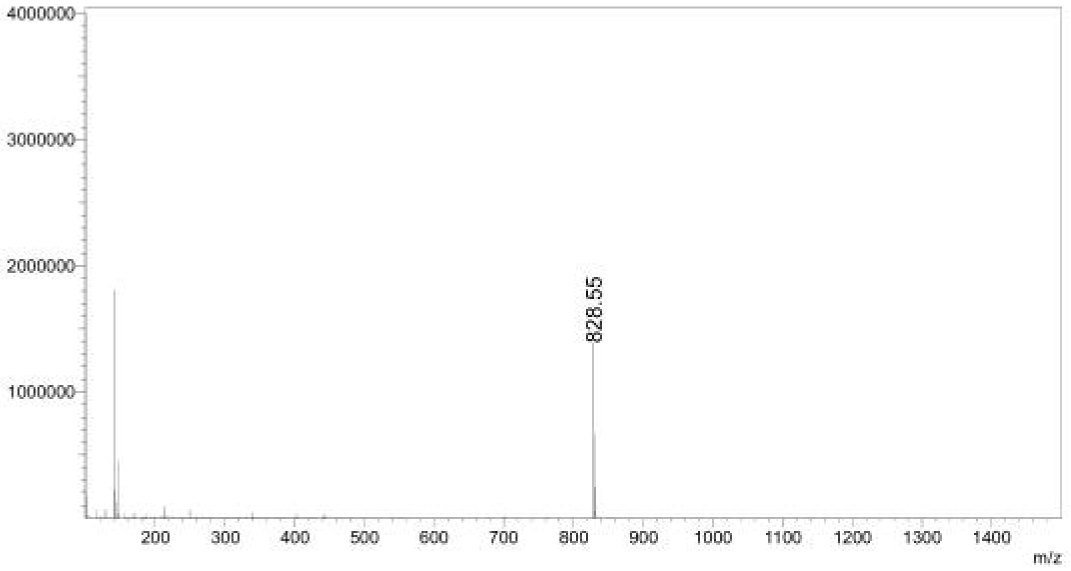

Z:¥Data¥2023¥04 April¥lab 112¥Moiseeva¥ma-266_MS_column_phaseB_DUIS-POS-100-1500_Moiseeva_AcN004_003.lcd

Figure S32. HPLC-MS spectrum and chromatogram of amine **4e**.

Display Report

Analysis Info

Analysis Name	D:\Data\Kolotyrkina\2021\Moiseeva\0310022.d	Acquisition Date	10.03.2021 13:04:38
Method	tune_50-1600.m	Operator	BDAL@DE
Sample Name	/BREL MA-266	Instrument / Ser#	micrOTOF 10248
Comment	C36H39NO12 mH 678.2545 calibrant added CH3CN		

Acquisition Parameter

Source Type	ESI	Ion Polarity	Positive	Set Nebulizer	1.0 Bar
Focus	Not active			Set Dry Heater	200 °C
Scan Begin	50 m/z	Set Capillary	4500 V	Set Dry Gas	4.0 l/min
Scan End	1600 m/z	Set End Plate Offset	-500 V	Set Divert Valve	Waste

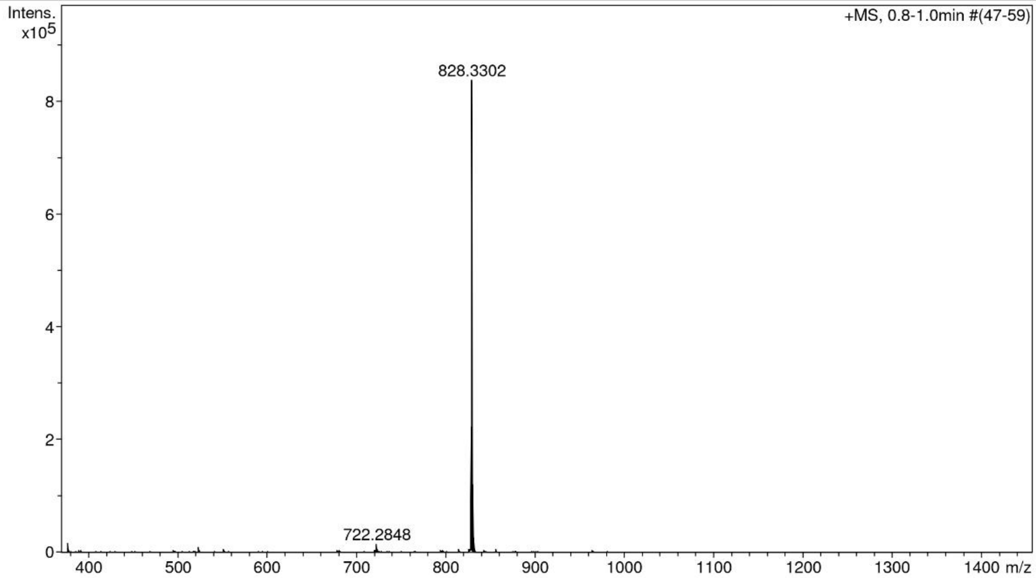

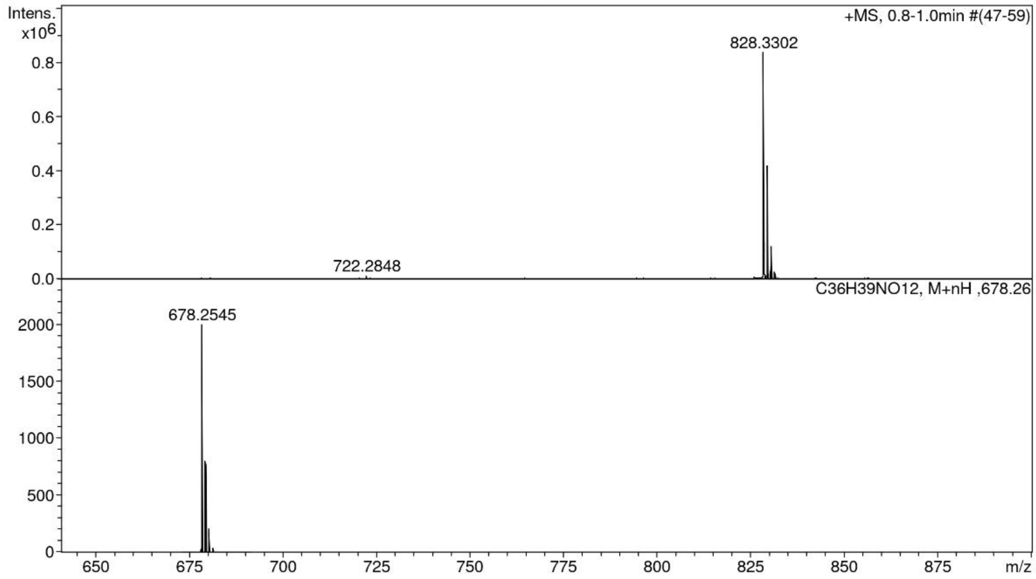

Bruker Compass DataAnalysis 4.0 printed: 10.03.2021 13:08:17 Page 1 of 1

Figure S33. HRMS spectrum of amine **4e**.

SHIMADZU LabSolutions

Analysis Report

<Sample Information>

Sample Name	: ma-262		
Sample ID	: Moiseeva_AcN006		
Data Filename	: ma-262_MS_no_column_phaseB-80%_DUIS_Moiseeva_AcN006_006.lcd		
Method Filename	: MS_no_column_phaseB-80%_DUIS.lcm		
Batch Filename	: Khakina_batch.lcb		
Vial #	: 1-6	Sample Type	: Unknown
Injection Volume	: 0.1 uL		
Date Acquired	: 4/6/2023 3:10:23 PM	Acquired by	: System Administrator
Date Processed	: 4/6/2023 3:13:26 PM	Processed by	: System Administrator

<Spectrum>

Line#:1 R.Time:0.292(Scan#:36)
MassPeaks:1572
RawMode:Single 0.292(36) BasePeak:708.50(2562896)
BG Mode:None Segment 1 - Event 1

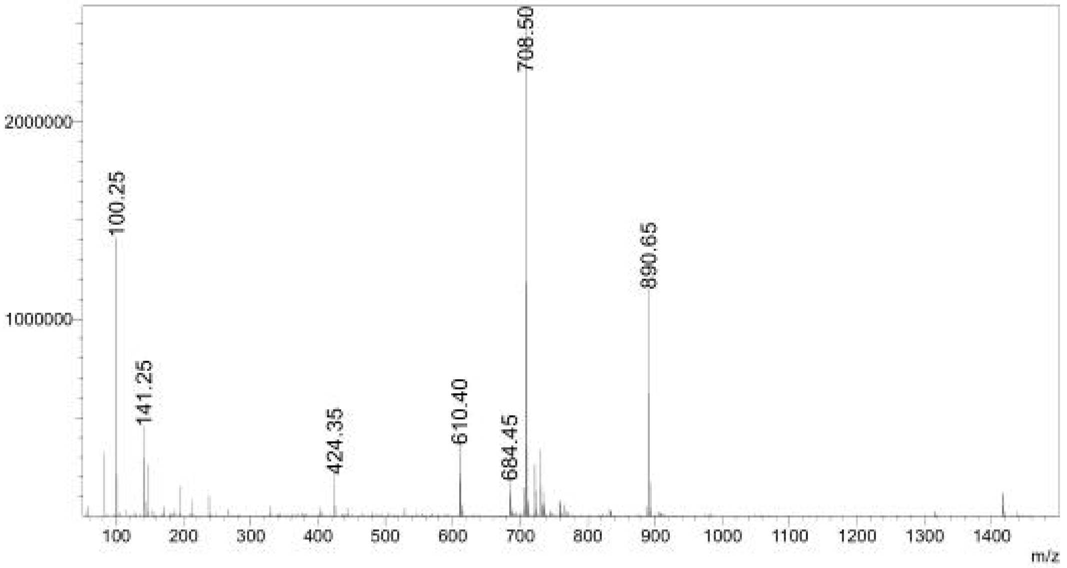

Figure S34. LC-MS spectrum of amine **4f**.

SHIMADZU LabSolutions

Analysis Report

<Sample Information>

Sample Name	: ma-262		
Sample ID	: Moiseeva_AcN006		
Data Filename	: ma-262_MS_column_phaseB_DUIS-POS-100-1500_Moiseeva_AcN006_005~1.lcd		
Method Filename	: MS_column_phaseB_DUIS-POS-100-1500.lcm		
Batch Filename	: Khakina_batch.lcb		
Vial #	: 1-6	Sample Type	: Unknown
Injection Volume	: 0.5 uL		
Date Acquired	: 4/6/2023 5:28:44 PM	Acquired by	: System Administrator
Date Processed	: 4/6/2023 6:28:45 PM	Processed by	: System Administrator

<Spectrum>

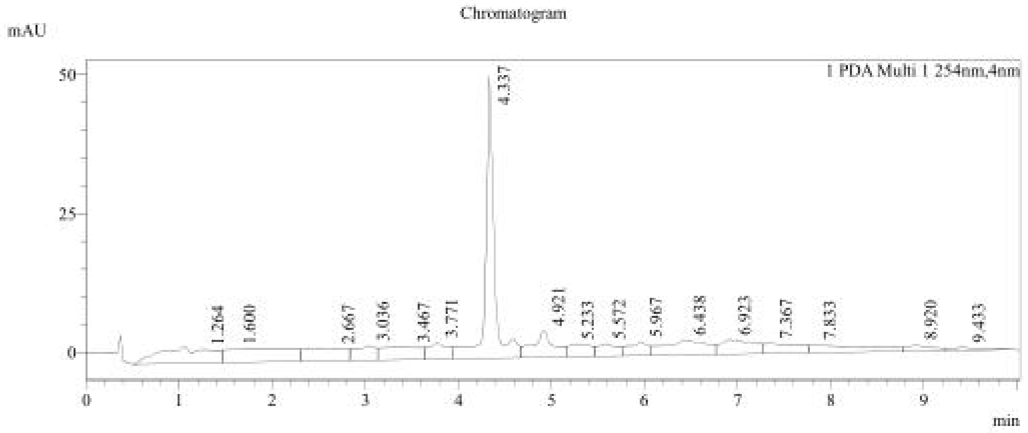

Line#:1 R.Time:4.333(Scan#:261)
MassPeaks:1565
RawMode:Single 4.333(261) BasePeak:708.45(5123334)
BG Mode:None Segment 1 - Event 1

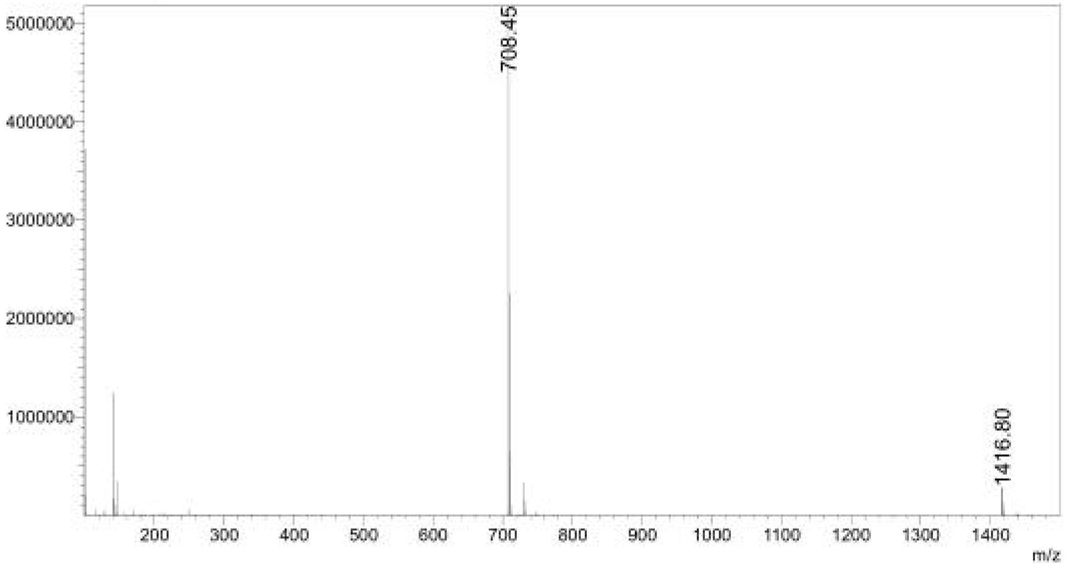

Figure S35. HPLC-MS spectrum and chromatogram of amine **4f**.

Display Report

Analysis Info

Analysis Name	D:\Data\Kolotyrkina\2021\Moiseeva\0310020.d	Acquisition Date	10.03.2021 12:55:33
Method	tune_50-1600.m	Operator	BDAL@DE
Sample Name	/BREL MA-262	Instrument / Ser#	micrOTOF 10248
Comment	C37H41NO13 mH 708.2650 calibrant added CH3CN		

Acquisition Parameter

Source Type	ESI	Ion Polarity	Positive	Set Nebulizer	1.0 Bar
Focus	Not active			Set Dry Heater	200 °C
Scan Begin	50 m/z	Set Capillary	4500 V	Set Dry Gas	4.0 l/min
Scan End	1600 m/z	Set End Plate Offset	-500 V	Set Divert Valve	Waste

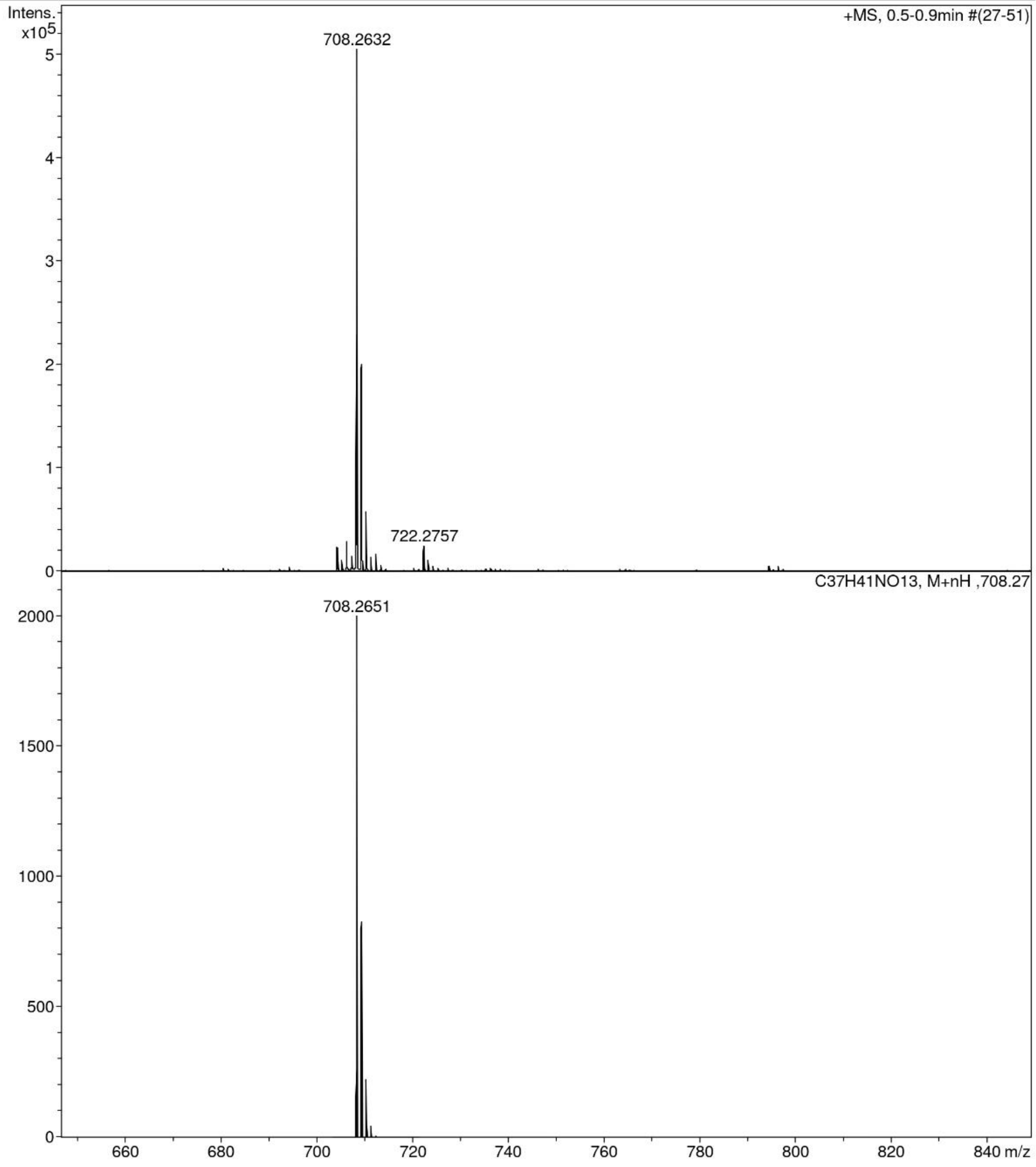

Bruker Compass DataAnalysis 4.0 printed: 10.03.2021 12:58:53 Page 1 of 1

Figure S36. HRMS spectrum of amine **4f**.

SHIMADZU LabSolutions

Analysis Report

<Sample Information>

Sample Name : gkg-1157
Sample ID : Moiseeva_AcN
Data Filename : gkg-1157_MS_no_column_phaseB-50%_DUIS_Moiseeva_AcN_002.lcd
Method Filename : MS_no_column_phaseB-50%_DUIS.lcm
Batch Filename : General_batch.lcb
Vial # : 1-3 Sample Type : Unknown
Injection Volume : 0.1 uL
Date Acquired : 9/14/2023 2:38:27 PM Acquired by : System Administrator
Date Processed : 9/14/2023 2:41:29 PM Processed by : System Administrator

<Spectrum>

Line#:1 R.Time:0.500(Scan#:61)
MassPeaks:1622
RawMode:Single 0.500(61) BasePeak:678.50(1579242)
BG Mode:None Segment 1 - Event 1

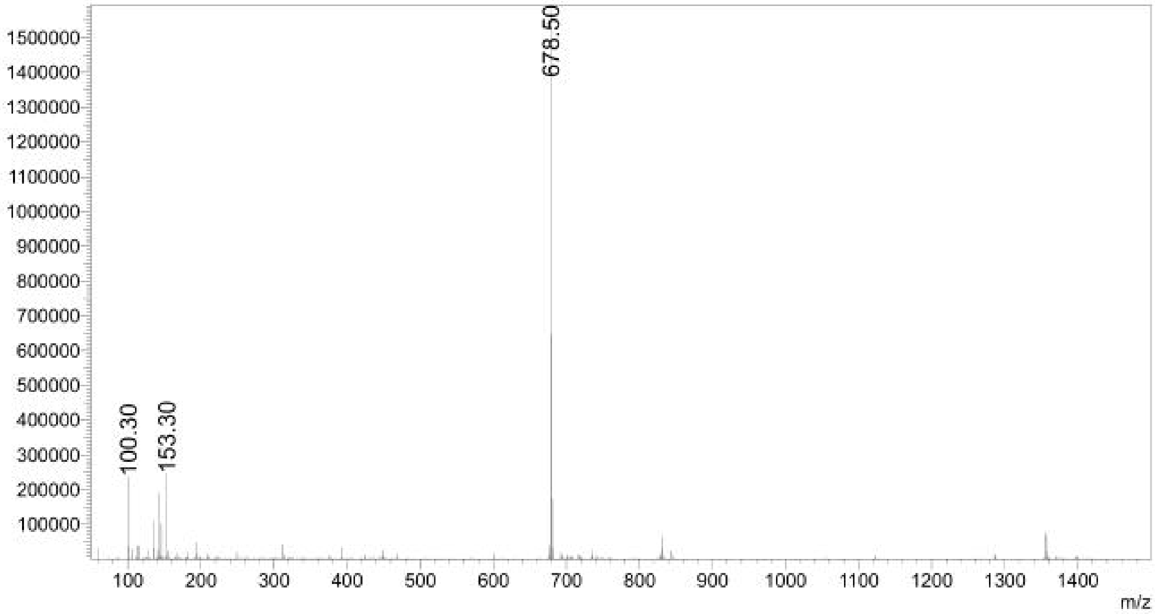

Figure S37. LC-MS spectrum of amine **4g**.

SHIMADZU LabSolutions

Analysis Report

<Sample Information>

Sample Name	: gkg-1157
Sample ID	: Moiseeva_AcN
Data Filename	: gkg-1157_MS_column_phaseB-30-80_DUIS-POS-100-1500-10MIN_Moiseeva_AcN_002
Method Filename	: MS_column_phaseB-30-80_DUIS-POS-100-1500-10MIN.lcm
Batch Filename	: General_batch.lcb
Vial #	: 1-3
Sample Type	: Unknown
Injection Volume	: 0.1 uL
Date Acquired	: 9/14/2023 3:21:55 PM
Acquired by	: System Administrator
Date Processed	: 9/14/2023 3:32:02 PM
Processed by	: System Administrator

<Spectrum>

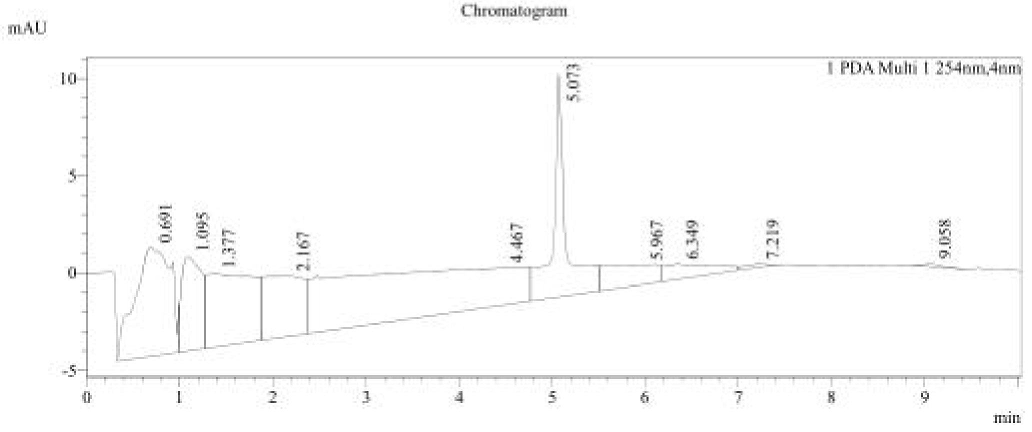

Line#:1 R.Time:5.100(Scan#:307)
MassPeaks:1589
RawMode:Single 5.100(307) BasePeak:100.20(1785313)
BG Mode:None Segment 1 - Event 1

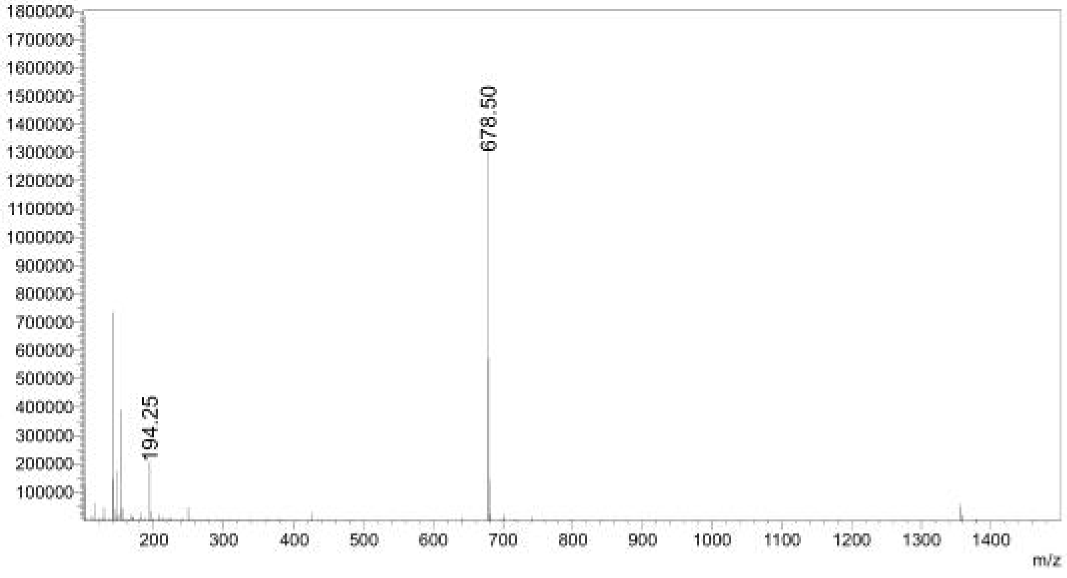

Figure S38

SHIMADZU LabSolutions **Analysis Report**

<Sample Information>

Sample Name	: esh-429
Sample ID	: Moiseeva_AcN
Data Filename	: esh-429_MS_no_column_phaseB-50%_DUIS_Moiseeva_AcN_001.lcd
Method Filename	: MS_no_column_phaseB-50%_DUIS.lcm
Batch Filename	: General_batch.lcb
Vial #	: 1-1
Sample Type	: Unknown
Injection Volume	: 0.1 uL
Date Acquired	: 9/14/2023 2:30:57 PM
Acquired by	: System Administrator
Date Processed	: 9/14/2023 2:34:00 PM
Processed by	: System Administrator

<Spectrum>

Line#:1 R.Time:0.583(Scan#:71)
MassPeaks:1589
RawMode:Single 0.583(71) BasePeak:708.50(3589228)
BG Mode:None Segment 1 - Event 1

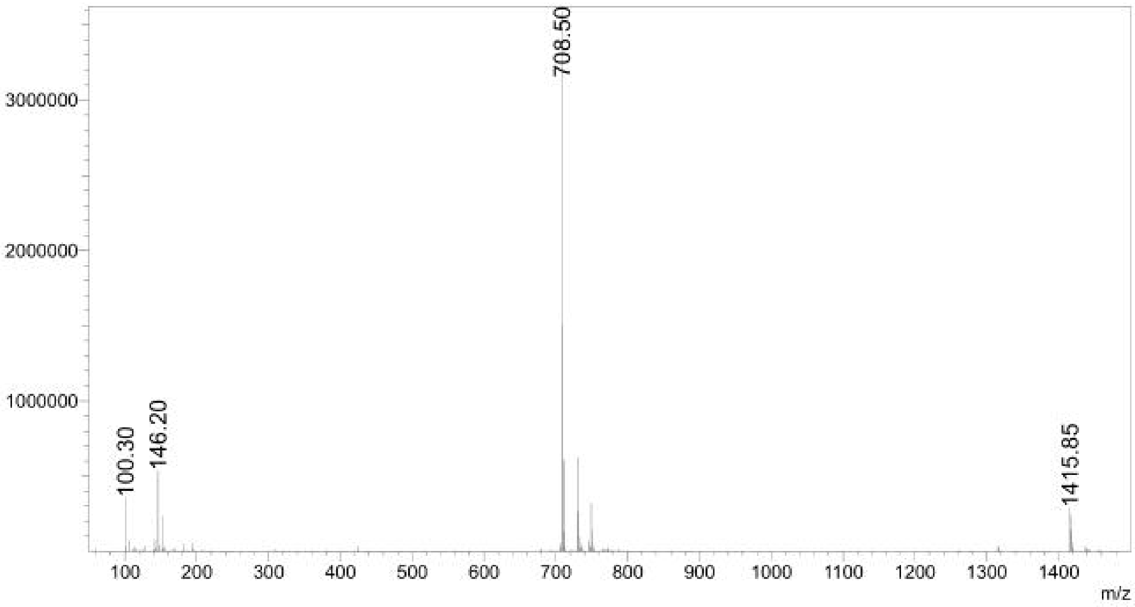

Figure S39. LC-MS spectrum of amine **4h**.

SHIMADZU LabSolutions

Analysis Report

<Sample Information>

Sample Name	: esh-429		
Sample ID	: Moiseeva_AcN		
Data Filename	: esh-429_MS_column_phaseB-30-80_DUIS-POS-100-1500-10MIN_Moiseeva_AcN_001.l		
Method Filename	: MS_column_phaseB-30-80_DUIS-POS-100-1500-10MIN.lcm		
Batch Filename	: General_batch.lcb		
Vial #	: 1-1	Sample Type	: Unknown
Injection Volume	: 0.1 uL		
Date Acquired	: 9/14/2023 3:00:17 PM	Acquired by	: System Administrator
Date Processed	: 9/14/2023 3:10:25 PM	Processed by	: System Administrator

<Spectrum>

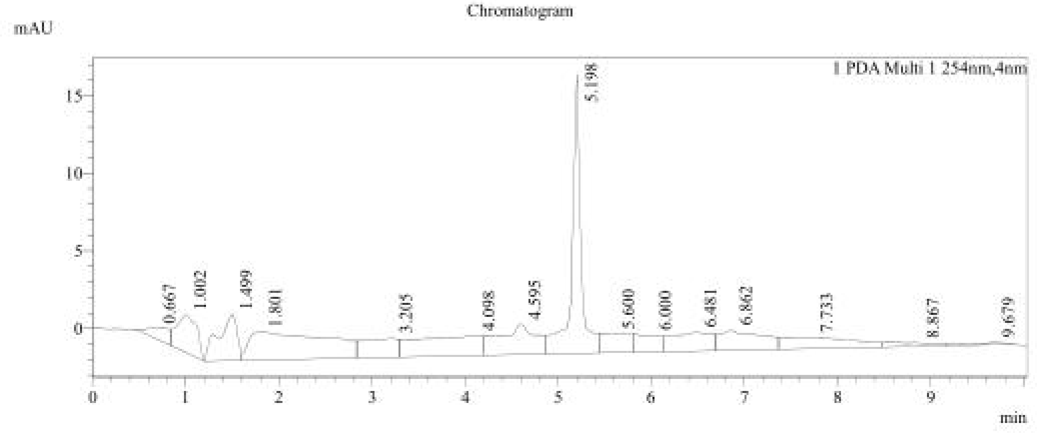

Line#:1 R.Time:5.233(Scan#:315)
MassPeaks:1529
RawMode:Single 5.233(315) BasePeak:100.20(1846483)
BG Mode:None Segment 1 - Event 1

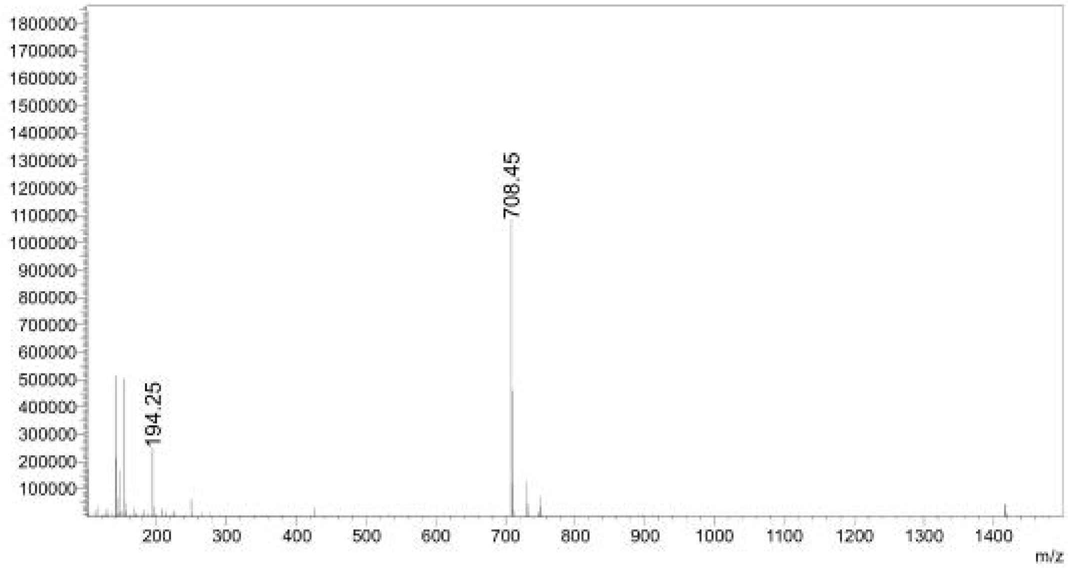

Figure S40. HPLC-MS spectrum and chromatogram of amine **4h**.

IR spectra of all compounds

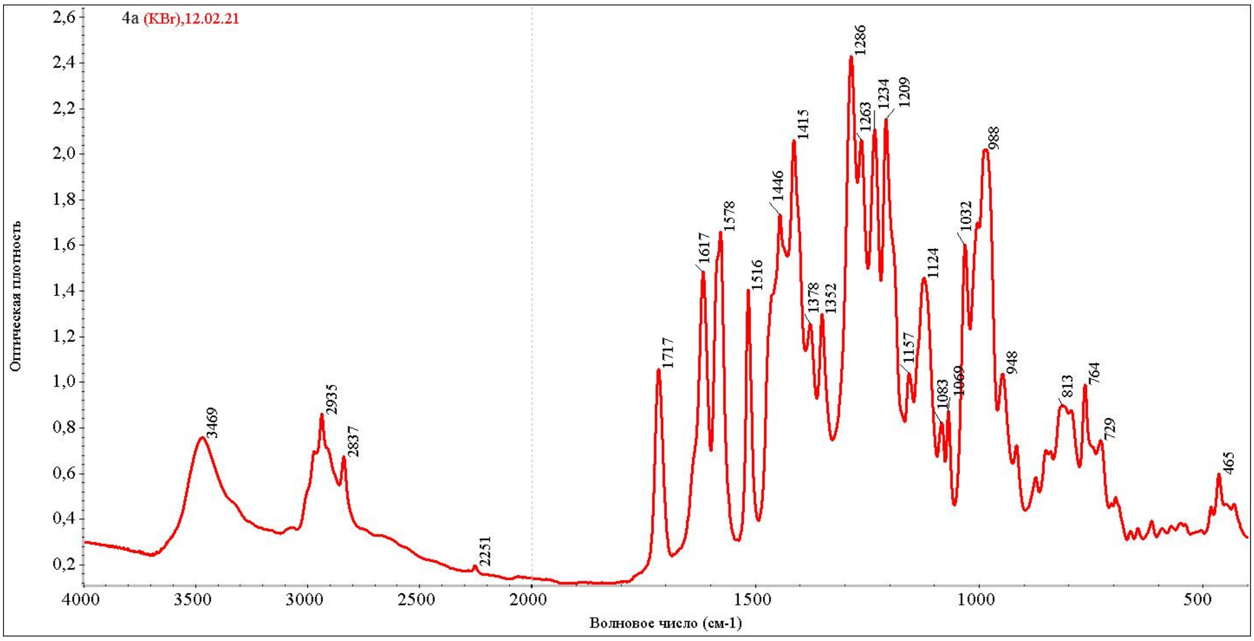

Figure S41. IR spectrum of amine **4a**.

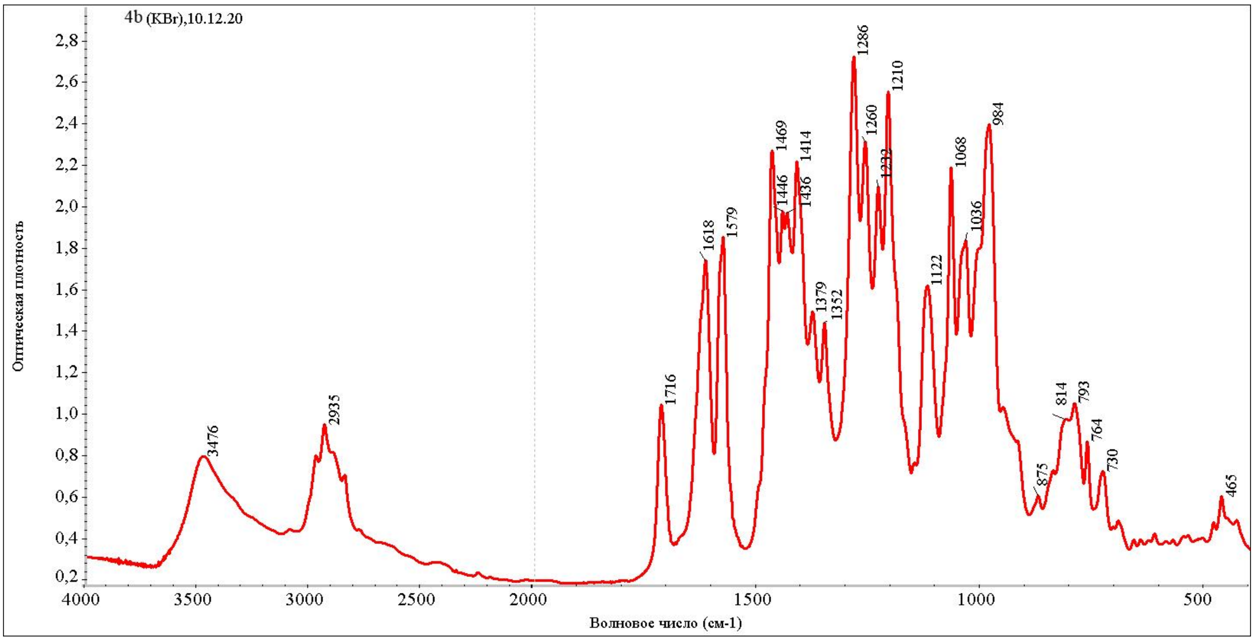

Figure S42. IR spectrum of amine **4b**.

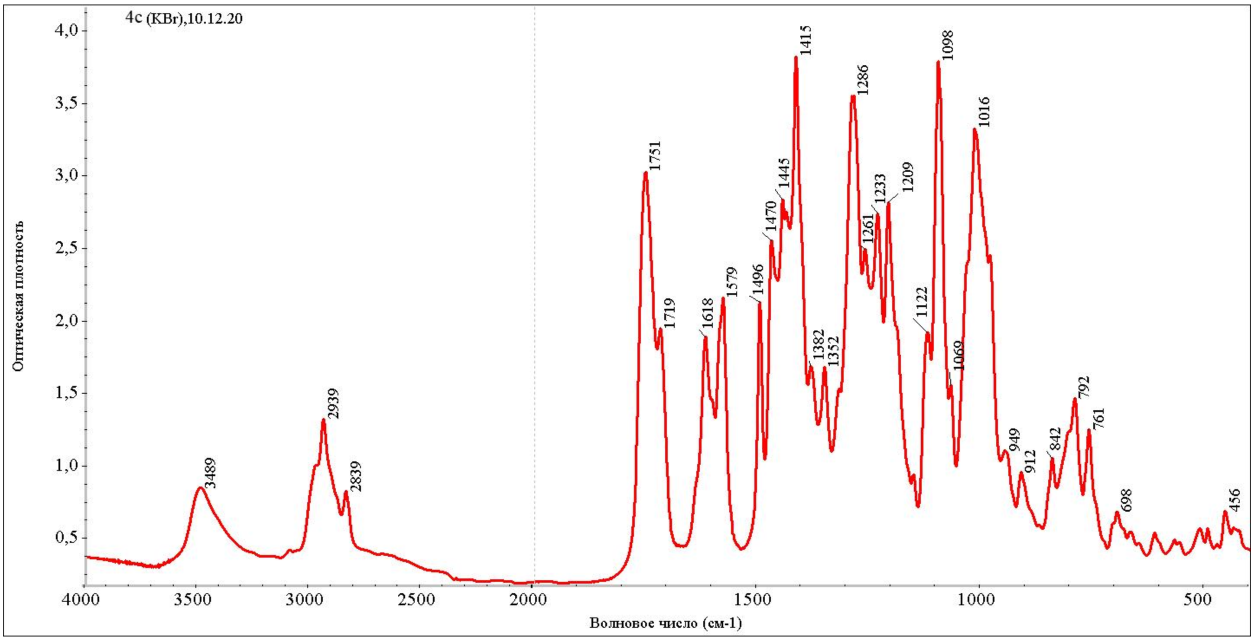

Figure S43. IR spectrum of amine **4c**.

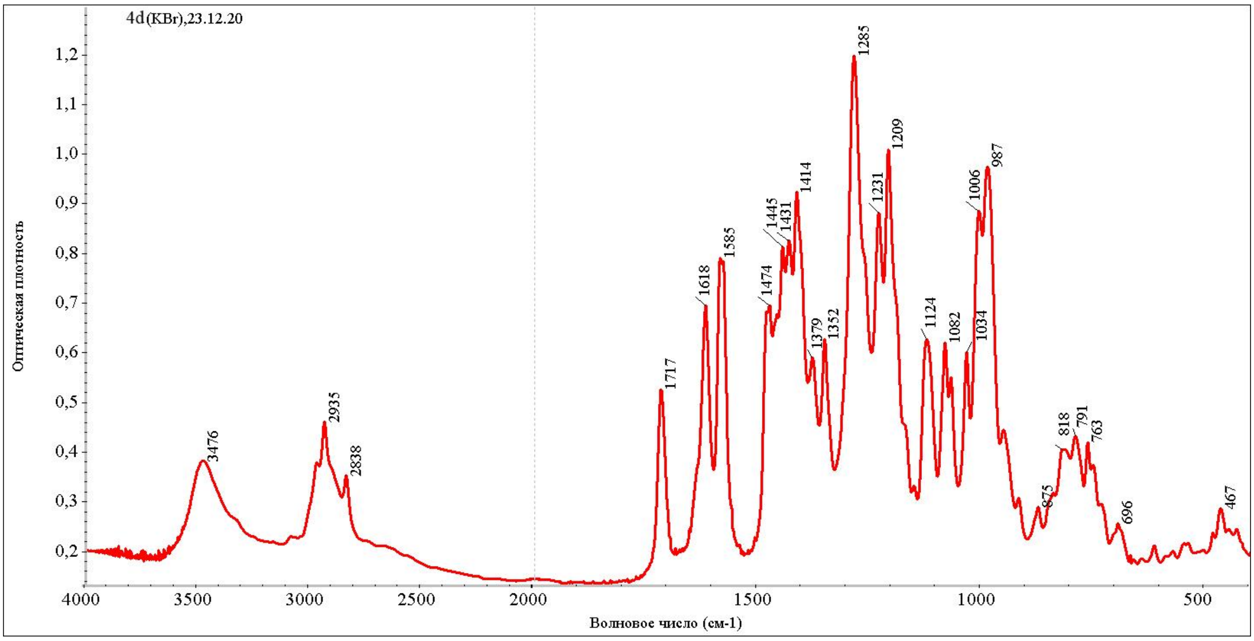

Figure S44. IR spectrum of amine **4d**.

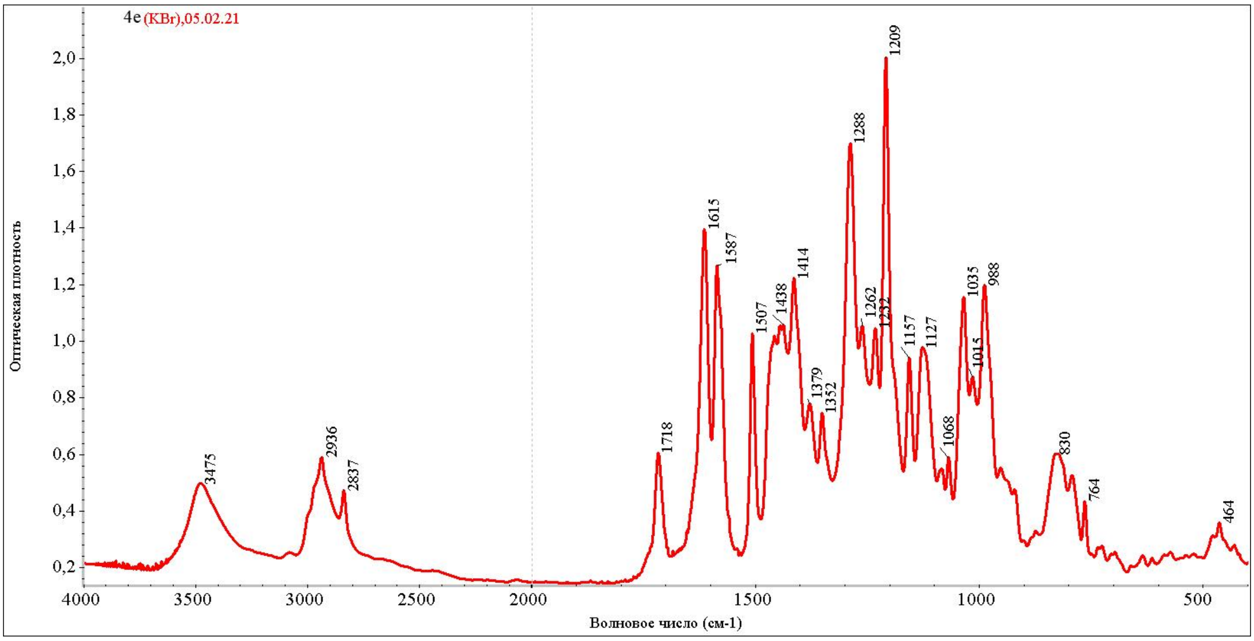

Figure S45. IR spectrum of amine **4e**.

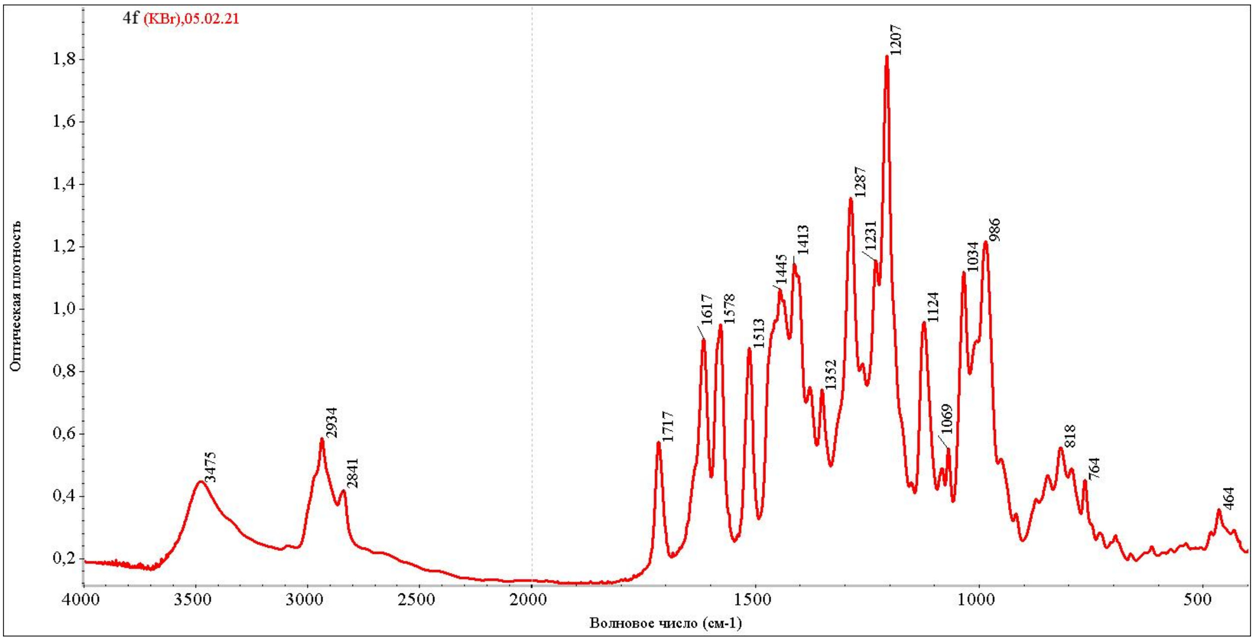

Figure S46. IR spectrum of amine **4f**.

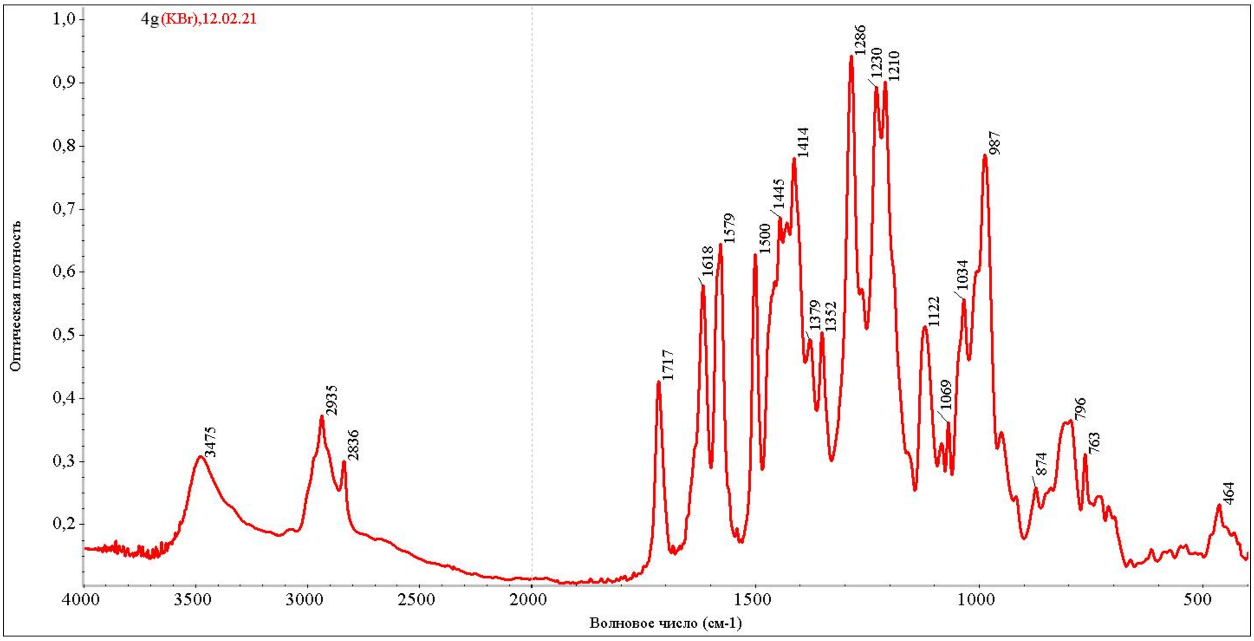

Figure S47. IR spectrum of amine **4g**.

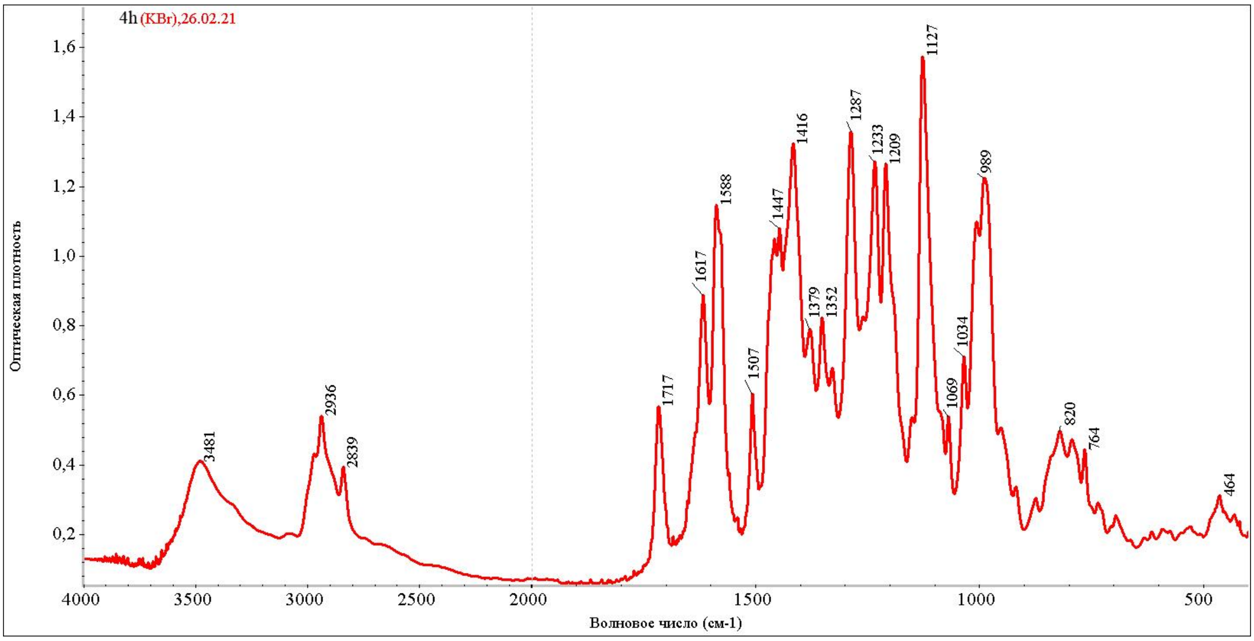

Figure S48. IR spectrum of amine **4h**.
